# Paenidepsins are a Family of Lipopeptides from *Paenibacillus*

**DOI:** 10.1021/acs.jnatprod.5c01579

**Published:** 2026-03-03

**Authors:** Daniel Torres-Püschel, Lukas Zimmer, Stefan Kehraus, Aia Ali Abdelrahman, Tanja Schneider, Anna Müller, Aurélien Carlier, Max Crüsemann

**Affiliations:** † Institute of Pharmaceutical Biology, 9374 University of Bonn, 53115 Bonn, Germany; ‡ Institute of Pharmaceutical Biology, 9173 Goethe-University Frankfurt, 60438 Frankfurt, Germany; § Institute for Pharmaceutical Microbiology, University of Bonn, University Hospital Bonn, 53115 Bonn, Germany; ∥ German Center for Infection Research (DZIF), Partner Site Bonn-Cologne, 53127 Bonn, Germany; ⊥ LIPME, Université de Toulouse, INRAE, CNRS, Castanet-Tolosan 31326, France

## Abstract

A new 12-membered cyclic lipopeptide with antifungal activity, paenidepsin A (**1**), was isolated from the common-wheat-associated bacterial strain *Paenibacillus apiarius* MW-14. Its chemical structure was elucidated using a combination of extensive tandem mass spectrometry (MS/MS) and nuclear magnetic resonance (NMR) spectroscopy experiments. Further paenidepsin analogs B–F (**2**–**6**) produced by the same strain were detected through molecular networking and structurally characterized via MS/MS analyses. Peptidogenomic analysis revealed the corresponding nonribosomal peptide synthetase (NRPS) biosynthetic gene cluster (BGC), while through genome mining, 72 homologous BGCs were identified from publicly available bacterial genomes. Comprehensive phylogenetic and comparative analyses demonstrated that paenidepsin-like BGCs constitute a new gene cluster family from *Paenibacillus*. Detailed NRPS domain-level analyses provided bioinformatic evidence for recombination-driven diversification of this BGC. Our findings illustrate the utility of integrating mass spectrometric and genomic tools to discover diverse natural products, and highlight the significant biosynthetic capacity of *Paenibacillus* species.

## Graphical abstract

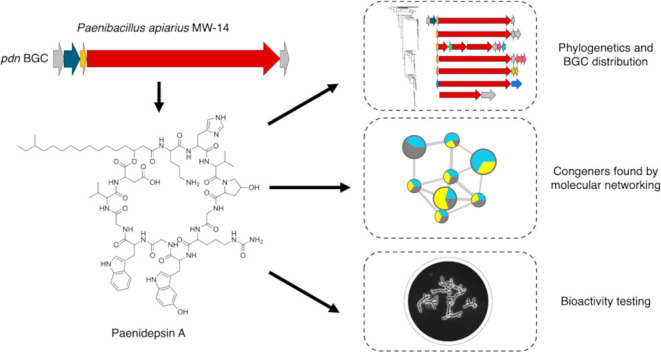

Natural products (NPs) are specialized metabolites produced by living organisms such as bacteria, fungi and plants.^1^ They are renowned for their remarkable structural diversity, having evolved under ecological pressure to mediate interactions within their environments.^2^ Historically—and continuing today—NPs have been a major source of novel drug leads.^1^ Among these, lipopeptides (LPs), a well-studied class of microbial metabolites, stand out for their structural complexity and therapeutic relevance.^3^

Lipopeptides are typically biosynthesized by modular nonribosomal peptide synthetase (NRPS) megaenzymes or by hybrid systems, including polyketide synthase modules.^4^ LPs display considerable structural diversity, which arises from variations in the length, saturation and branching of the fatty acid chain, as well as from differences in the amino acid composition of the peptide moiety. This peptide portion is predominantly cyclized, although some examples of linear LPs have been reported,^3,5–7^ further contributing to their chemical and functional diversity. This variability gives rise to a wide range of bioactivities, which includes antibacterial, antiviral, antifungal, antitumor and immunomodulatory effects.^3^ Lipopeptides from genera such as *Bacillus*, *Streptomyces*, *Pseudomonas* and *Paenibacillus* have been extensively studied for their antimicrobial potential. Clinically relevant examples include daptomycin from *Streptomyces roseosporus* ^7^ and polymyxin B from *Paenibacillus* *polymyxa*,^3,8^ underscoring the therapeutic relevance of this compound class and the bacterial genera that produce these compounds.

*Paenibacillus* is a genus of Gram-positive, facultatively anaerobic, endospore-forming bacteria found in various ecosystems, with several members being notably adapted to rhizospheric and soil niches.^9,10^ Since its original description in 1993, this genus has undergone several taxonomic revisions.^11^ Many *Paenibacillus* strains are known to produce structurally diverse secondary metabolites, including lipopeptides with antimicrobial and biocontrol properties.^12,13^ Both cyclic and linear antimicrobial LPs, with peptide chain lengths between 6 and 13 amino acids, and numerous variations in their fatty acid chains, have been isolated from *Paenibacillus* strains. Examples of cationic LPs with antibacterial activity against Gram-negative and/or -positive bacteria include polymyxins (cyclic lipodecapeptides with C8,9-unsubstituted fatty acid),^14^ octapeptins (cyclic lipooctapeptides, β-hydroxy fatty acid),^15^ paenibacterin (cyclic lipotridecapeptides, pentadecanoic acid),^16^ and pelgipeptins (cyclic lipononapeptides, β-hydroxy fatty acid).^17,18^ Noncationic LPs from *Paenibacillus* include the antifungal fusaricidins and related LiF-antibiotics (cyclic lipohexapeptides containing a guanidinylated β-hydroxy fatty acid),^19,20^ antibacterial paenilipoheptins (cyclic lipoheptapeptides, β-amino fatty acid),^21^ and anti-Gram-negative tridecaptins (linear lipotridecapeptides, β-hydroxy fatty acid).^6^ Still, much of the genus’ full biosynthetic potential remains underexplored.^22,23^

The persistent challenge of rediscovering known natural products, coupled with the pressing demand for novel molecular scaffolds, emphasizes the importance of innovative screening strategies.^24^ Genome mining has emerged as a powerful approach for uncovering new biosynthetic gene clusters (BGCs), enabling the targeted discovery of novel antibiotics.^25^ Crucially, linking chemical structures to their corresponding BGCs advances our understanding of structure–function relationships and lays the groundwork for future biosynthetic engineering.^26^ Comparative phylogenomic and recombination analyses further enrich this framework by uncovering evolutionary trajectories and diversification patterns within BGC families.^27^

In the search for novel bioactive metabolites, we isolated an antifungal compound, paenidepsin A, from the culture broth of a *Paenibacillus apiarius* strain. Paenidepsin A is a 12-membered macrocyclic lipopeptide that contains a 3-hydroxy-14-methylpalmitoyl moiety, and is produced by a 12-modular NRPS system. Through genome mining, we identified homologous paenidepsin (*pdn*) BGCs within other *Paenibacillus* strains, indicating that this lipopeptide family is moderately distributed across the genus. Comparative analysis of these clusters suggests evolutionary diversification of the *pdn* BGC and the encoded lipopeptides. These findings underscore *Paenibacillus* as a promising reservoir of structurally and functionally diverse lipopeptides for drug discovery.

## Results and Discussion

### Initial Screening and BGC Identification

In a previous study, we screened a collection of 258 plant-associated bacterial strains from the phyllosphere and rhizosphere of *Arabidopsis thaliana* for antimicrobial activity in single or cocultures.^28^ Among these, *Paenibacillus* sp. P3_m182_1 inhibited the growth of *Staphylococcus aureus* LMG 10147 in monoculture. Cultivation of this strain led to the detection of a large peptide metabolite (*M*_W_: 1472 Da), which sparked our interest (Figure S1). Despite various cultivation strategies, including variation of media and cultivation parameters, and cocultivation with *Bacillus subtilis* JH642 + sfp and *Eurotium rubrum* DSM 62631, we were unable to isolate this peptide in preparative amounts from this strain. However, the extracts from these experiments (30 in total) were later included in a comparative metabolomic analysis using global natural product social (GNPS) molecular networking (see section Characterization of paenidepsin A congeners), to investigate peptide diversity.

Consequently, we next employed a peptidogenomic workflow to link tandem mass spectrometry (MS/MS) data (Figure S1) to biosynthetic genes,^29^ identifying a candidate 12-module NRPS BGC, which we termed *pdn*, comprising two NRPS genes (Figure S2). The encoded NRPS system begins with a starter condensation (*C*_Starter_) domain and the modules 7 and 9 contain an epimerization (E) domain, suggesting the presence of d-configurated residues in the final peptide (see section Analysis of Paenidepsin Biosynthesis for a more detailed description).

In the search for alternative producers, we detected a homologous NRPS BGC in the genome of *P. apiarius* MW-14.^30^ Comparative LC–MS/MS analyses revealed that strain MW-14 produced a similar compound with *m*/*z* 831.95 ([M + 2H]^2+^) (**1**). The peptide was produced in higher amounts under multiple conditions (up to 26-fold compared to extracts of strain P3_m182_1, Figure S3), which led us to select this strain for scale-up and preparative isolation of the lipopeptides.

### Isolation and Structure Elucidation of Paenidepsin A (**1**)

We then performed a 2 L cultivation of *P. apiarius* MW-14 in tryptic soy broth (TSB) medium, and the cell pellet was extracted with *n*-butanol, since **1** was found to be mostly present in the cell pellet (Figure S3). After several chromatographic separation and purification procedures, which included flash chromatography and HPLC, 0.5 mg of paenidepsin A (**1**) were isolated. This compound is a macrocyclic lipopeptidolactone formed by 12 amino acids and a 3-hydroxy-14-methylpalmitoyl moiety (Figure 1). The *m*/*z* value of **1** was found to be 1662.8956 ([M + H]^+^), which corresponds to the molecular formula C_81_H_119_N_19_O_19_ (theoretical [M + H]^+^ *m*/*z*: 1662.8930, deviation: −2.82 ppm) (Figure S4) and is consistent with the NMR data (Figures 2, S5–S12 and Table S1).

**Figure 1:**
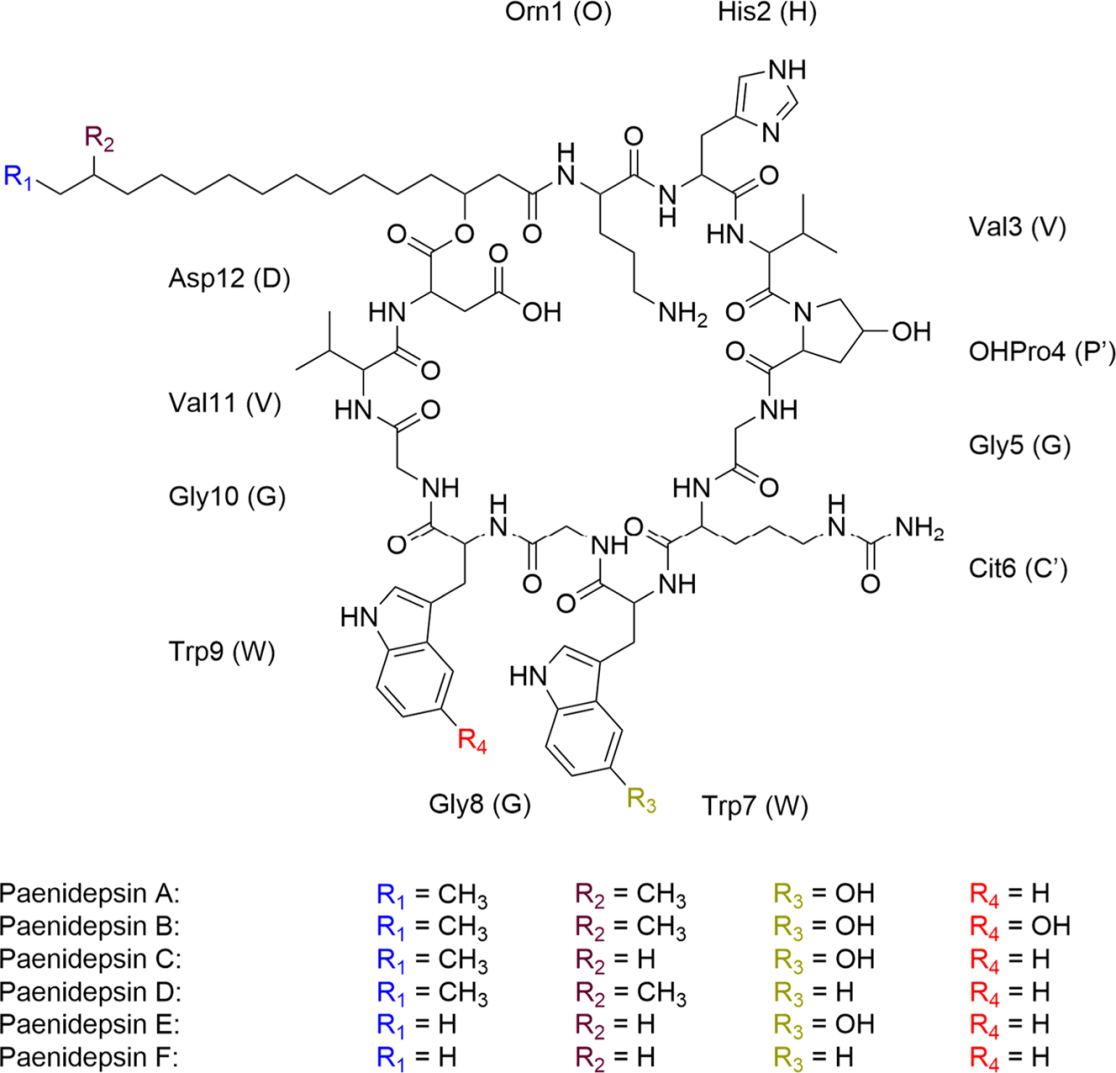
Chemical structure of paenidepsin A (**1**) and proposed structures of paenidepsin congeners B–F (**2**–**6**), based on extensive MS/MS experiments. Residues in color indicate the structural changes. Nonproteinogenic building blocks of paenidepsin are named: Orn = ornithine, OHPro = 4-hydroxyproline, Cit = citrulline.

**Figure 2:**
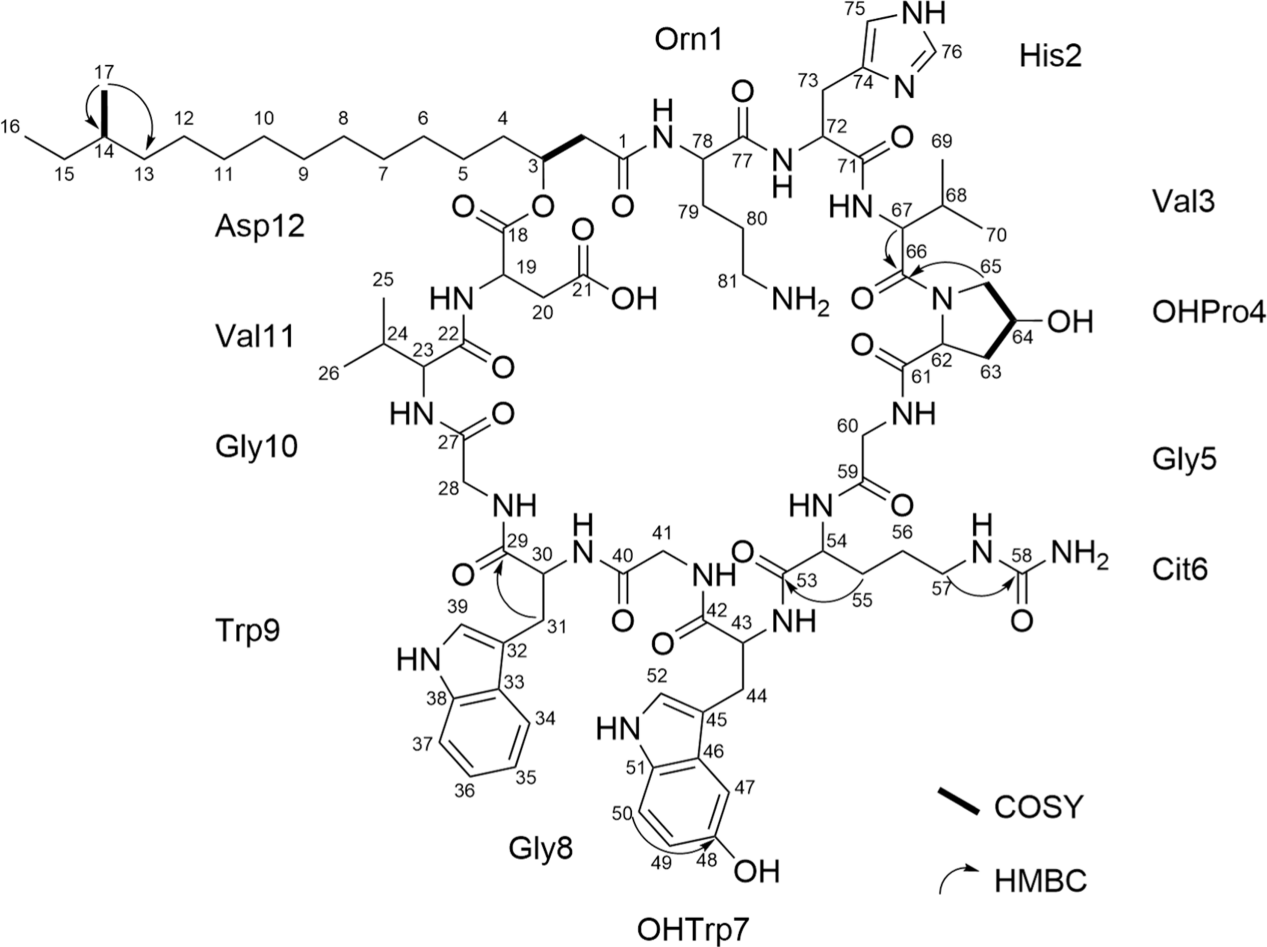
Key 2D NMR correlations supporting the sequence elucidation of paenidepsin A (**1**).

The peptidic structure of **1** was supported by the resonances for the α-amino methine proton signals (δ_H_ 4.3–5.1, δ_C_ 50.3–60.0) in the ^1^H and ^13^C spectra, respectively, indicative of peptide bonds. The structural assignments of the standard amino acids (His2, Val3, Gly5, Gly8, Trp9, Gly10, Val11 and Asp12), nonproteinogenic amino acids ornithine (Orn1) 4-hydroxyproline (OHPro4), citrulline (Cit6) and 5-hydroxytryptohan (OHTrp7), as well as the acyl component were made by analyses of the 1D (^1^H, ^13^C and DEPT) (Figures S5–S7), and 2D (COSY, HSQC, HMBC, ROESY and TOCSY) NMR data (Table S1 and Figures S8–S12). The position of Val3 is indicated through a HMBC correlation of H-67 to C-66. The position of OHPro4 was deduced by a HMBC correlation of H-65 to C-66 and the position of the hydroxy group was proven by COSY correlations from H-64 to both H_2_-63 and H_2_-65 (Figure S8). The presence and position of Cit6 was indicated by the HMBC correlations H_2_-57 to C-58 and H_2_-55 to C-53, respectively (Figure S10). The position of the hydroxy group of OHTrp7 was deduced by a strong HMBC correlation of H-50 to C-48 and the position of Trp9 by the HMBC correlation of H_2_-31 to C-29. Concerning the acyl component, the 3-hydroxybutyryl unit was clear from a COSY correlation of H_2_-2 to H-3 (Figure S8). The position of methyl group CH_3_-17 at C-14 was evident from HMBC correlations of H_3_-17 to both C-13 and C-14 (Figure S10). Additionally, a COSY correlation from H_3_-17 to H-14 was observed, further proving the position of CH_3_-17. The NMR data together with the molecular weight of compound **1** proved the acyl chain to be a 3-hydroxy-14-methylpalmitoyl moiety. The 12 amino acids and the acyl component account for all the atoms present in **1** and for 31 out of the 32 degrees of unsaturation required by the molecular formula. The remaining unsaturation is due to the cyclic nature of **1**. The amino acid sequence was unequivocally determined by integrating the interpretation of the 2D NMR spectra (Figure 2) with de novo sequencing of the MS/MS fragmentation pattern of **1** (Figure S4). The corresponding MS/MS spectrum exhibited a contiguous series of fragment ions, which include characteristic b-ions, b-ions with loss of water (*b*°), acyl chain-loss variants (*b* ^#^), and b-ions with neutral loss of isocyanic acid (b*), a fragmentation commonly associated with citrullinated peptides.^31^ These ions confirmed the observed HMBC correlations concerning the position of the amino acids along the backbone and further allowed the full sequence elucidation of **1**. This combined analysis allowed the confident assignment of the peptide sequence, and the planar structure of paenidepsin A (**1**) was consequently elucidated (Figures 1 and 2). Limitations of pure material precluded Marfey’s analysis of (**1**) to experimentally determine amino acid stereochemistry. The structures are therefore shown as planar representations. However, bioinformatic analysis of the *pdn* locus gives predictions about amino acid configurations, which are outlined below (see Analysis of Paenidepsin Biosynthesis).

Two studies from 2013 documented the isolation of the cyclic lipopeptide KB425796-A along with ten of its congeners, KB425796–B–K, from *Paenibacillus* sp. 530,603.^32,33^ Paenidepsin A (**1**) shares most structural features with these lipopeptides, notably the 12-amino acid macrolactone ring and the acyl chain length and structure of KB425796–F. However, **1** possesses a hydroxylated proline residue at C-4 (δ_C_ 70.5 ppm, δ_H_ 4.49 ppm (m), see Table S1), whereas peptides of the KB425796 series contain a proline at this position (Table S2).

### Characterization of Paenidepsin A Congeners

To investigate the chemical diversity of lipopeptides produced by *P. apiarius* MW-14, we performed a metabolomic analysis of the samples coming from our initial efforts to enhance the production of lipopeptides using MS/MS-based GNPS molecular networking.^34^ Altogether, 30 samples were analyzed, revealing a molecular network of 178 clusters and 472 singletons (Figure S13), which included a cluster corresponding to the molecular family of **1** (Figure S14). Within this family, we identified seven congeners of **1**. These compounds predominantly formed double protonated [M + 2H]^2+^ species, although we also observed singly charged ions with lower intensity.

The *m*/*z* of the paenidepsin B (**2**) proton adduct, derived from the observed double-charged species ([M + 2H]^2+^ *m*/*z*: 839.9524) was found to be 1678.8974 (theoretical: [M + H]^+^ 1678.8951; deviation: 1.37 ppm), corresponding to the molecular formula C_81_H_119_N_19_O_20_, i.e. with one additional oxygen atom compared to paenidepsin A (**1**), suggesting an additional hydroxylation. The MS/MS fragmentation pattern of **2** closely resembled that of **1**, including a delta mass corresponding to an acyl chain of the same length (Figure S15). Based on the structure of **1**, we propose that the acyl chain of **2** also derives from 3-hydroxy-14-methylhexadecanoic acid with specific shifts supporting the proposed modification as highlighted in Figure S15. The MS2 spectrum also shows that Trp9 is hydroxylated.

Analogously, we propose the tentative structures of paenidepsin C (**3**), D (**4**), E (**5**) and F (**6**) through analysis and comparison of their MS/MS spectra and exact masses (Figures S16–S19). Their corresponding *m*/*z* values, derived from the observed double-charged species [M + 2H]^2+^, deviations from calculated *m*/*z*, and molecular formulas, are **3** (1648.8852, 0.42 ppm, C_80_H_117_N_19_O_19_), **4** (1646.9051, −0.18 ppm, C_81_H_119_N_19_O_18_), **5** (1634.8714, 1.53 ppm, C_79_H_115_N_19_O_19_), and **6** (1618.8718, −1.36 ppm, C_79_H_115_N_19_O_18_). These compounds differ in the hydroxylation pattern of Trp7 and Trp9 and in the fatty acid moiety. Similar to paenidepsin A and B, paenidepsin D features a 3-hydroxy-14-methylhexadecanoyl moiety (Figure S17), paenidepsins E and F a 3-hydroxy-pentadecanoyl moiety (Figures S18 and S19) and paenidepsin C a 3-hydroxy-hexadecanoyl acyl chain (Figure S16), also based on analogies to previously reported structures from *Paenibacillus* sp. 530,603.^33^ These features have been found in other bacterial lipopeptides, containing these or structurally related acyl chain moieties as well.^33,35–43^ One additional node, labeled congener G, showed insufficient MS2 quality; consequently, no structural assignment or MS2 spectrum is reported. All proposed structures are based on educated interpretations of fragmentation patterns and accurate mass data (Figures S15–S19).

With the acquired knowledge from elucidating and proposing the structures of the paenidepsin series, we also propose the structure of compound **7** (derived [M + H]^+^ *m*/*z* value from observed [M + 2H]^2+^ *m*/*z*: 1472.8608, deviation from calculated [M + H]^+^ *m*/*z* for C_72_H_113_N_17_O_16_: −1.02 ppm). This compound was produced by *Paenibacillus* sp. P3_m182_1 as shown in Figure S1, correlating with the predicted adenylation (A) domain specificities of the corresponding *pdn* BGC (Figure S2, see below). Briefly, compound **7** differs from the compounds produced by *P. apiarius* MW-14 in four residues: at position four, Pro replaces OHPro, at position six, Thr substitutes for citrulline, at position seven, Leu is incorporated instead of Trp/OHTrp, and the C-terminal residue is Asn rather than Asp. The precise structural confirmation of paenidepsin congeners **2**–**7** and assessment of their antifungal activities will require further preparative-scale isolation and spectroscopic characterization.

### Biological Activity of Paenidepsin A

To assess the antibacterial and antifungal activity of **1**, we determined minimal inhibitory and minimal effective concentrations (MIC/MEC) against a small panel of bacterial and fungal pathogens (Table S3). Paenidepsin A did not exhibit antibacterial activity against *Pseudomonas aeruginosa* PAO1, *S. aureus* HG001, nor against clinical *Candida albicans* isolates and *Aspergillus fumigatus* DSM819 with MICs >64 μg/mL. This observation prompted us to re-evaluate the activities of the paenidepsin producer strains *Paenibacillus* sp. P3_m182_1 and *P. apiarius* MW14 against the *S. aureus* isolates ATCC 29213 and HG001 (Figure S20). In comparison, *P. apiarius* MW14 shows highly reduced activity, implying that the initially observed antistaphylococcal activity by *Paenibacillus* sp. P3_m182_1 cannot be attributed to the paenidepsins, since its main paenidepsin (**7**) is produced in much lower amounts than paenidepsin A (**1**) under these conditions. While paenidepsin A had no antifungal activity against *C. albicans* on its own, a MIC of 8 μg/mL was determined when *C. albicans* I-11301 was treated in combination with 0.05 μg/mL micafungin (Table S3). Nonetheless, *A. fumigatus* displayed pronounced morphological aberrations after treatment with paenidepsin A, including irregular, bulbous swellings along the hyphal elements beginning at 2 μg/mL (=MEC) (Figure 3B).

**Figure 3:**
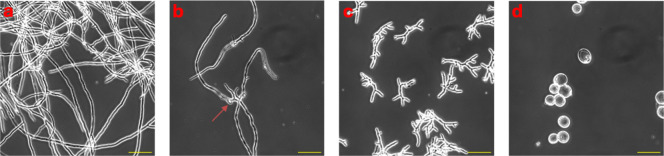
Morphological changes in *A. fumigatus* hyphal structures after treatment with paenidepsin A (**1**) alone or in combination with micafungin. While **1** alone induced irregular, bulbous swellings along the hyphal elements, treatment in combination with micafungin (0.015625 μg/mL) resulted in spherical, blastospore-like structures and filamentous architecture was largely abolished. Light microscopy images of (A) control cells, and cells treated with (B) paenidepsin A 2 μg/mL, (C) micafungin 0.015625 μg/mL and (D) paenidepsin A 32 μg/mL + micafungin 0.01562 μg/mL after 17 h incubation time. Scale bar 50 μm.

Hyphal metabolic activity is highest at the tip, consistent with the apical growth of filamentous fungi. Echinocandins such as micafungin inhibit the 1,3-β-d-glucan synthase at the hyphal tips, interfering with cell wall biosynthesis, leading to short, branched and aberrant hyphal stumps (Figure 3C).^44^ Complementary hyphal damage was observed when *A. fumigatus* was treated with paenidepsin A in combination with micafungin (0.015625 μg/mL), which could be attributed to a complementary effect. Filamentous morphology was almost completely absent and replaced by spherical, blastospore-like structures (Figure 3D). The noted changes remained unaltered after prolonged treatment for 48 h with increasing concentrations of paenidepsin A (Figure S21). Similar effects were observed for KB425796-A and congeners produced by *Paenibacillus* sp. 530,603.^33^

### Analysis of Paenidepsin Biosynthesis

The *pdn* BGC of *P. apiarius* MW-14 encodes one giant NRPS megaenzyme with 12 modules, where the predicted amino acids incorporated by the A domains align with the elucidated peptide sequence of **1**. The first module contains a *C*_Starter_ domain, predicted to catalyze the acylation of the initiating amino acid ornithine with 3-hydroxy-14-methylhexadecanoic acid, 3-hydroxy-pentadecanoic acid, or 3-hydroxy-hexadecanoic acid, depending on which congener is being biosynthesized (Figure 4). Bioinformatic predictions by antiSMASH 8.0 ^45^ also indicate the presence of catalytically active epimerization (E) domains in both modules 7 and 9. This suggests the incorporation of d-configured tryptophan residues at these positions in compounds **1**–**6**, while all other amino acid building blocks in the peptides are predicted to be l-configured (Figure 1). The final thioesterase (TE) domain is proposed to catalyze macrocyclization via ester bond formation between the hydroxyl group of the fatty acid and the carbonyl group of the terminal aspartic acid residue. The A domain specificity is dictated by the Stachelhaus code, which has been used for the structure prediction of NRPS-produced peptides of microbial origin. Recently, a new machine-learning based bioinformatic tool, PARAS, was reported to have improved prediction ability.^46^ PARAS was able to accurately predict the incorporation of histidine instead of tyrosine, as predicted by antiSMASH, and it could predict the incorporation of tryptophan by modules seven and nine (Table S4). PARAS was also utilized to analyze and compare the A domain signatures of the modules four and six to similar A domains reported in the literature, which incorporate hydroxyproline and citrulline, respectively. In the case of module four, A domains known to incorporate OHPro typically show high bioinformatic prediction scores (Table S5), whereas module four of *pdn* BGC shows a low prediction score for proline, suggesting that its A domain may inherently accept hydroxyproline, albeit with a noncanonical recognition pattern poorly captured by current bioinformatic tools. For module six, incorporation of ornithine is falsely predicted (Table S4), whereas, in the literature, there are only few examples of A domains known to incorporate citrulline, with varying prediction scores (Table S6). This shows that despite PARAS being a promising tool, its prediction capabilities for nonproteinogenic amino acids still lack reliability and accuracy. In the scope of this ambiguity and the lack of adenylation assay data in the literature, experimental confirmation is required to conclusively identify the actual substrate(s) for both modules.

**Figure 4:**
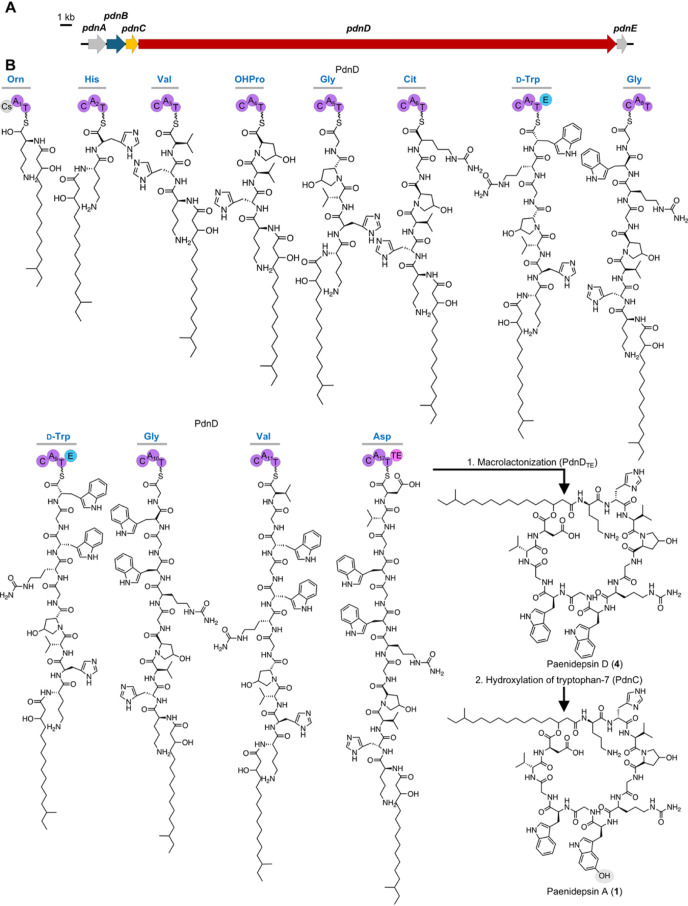
(A) Organization of the *pdn* BGC from *P. apiarius* MW-14 (gray = regulator proteins, blue = transporter protein, yellow = modifying enzyme, red = NRPS). (B) Proposed biosynthetic pathway of **1**. First, the *C*_Starter_ domain of NRPS PdnG catalyzes the N-acylation of ornithine with 3-hydroxy-14-methylhexadecanoic acid, then, PdnD continues the elongation to form a 12-membered linear peptide chain which undergoes macrocyclization catalyzed by PdnD_TE_ to release **4**. Then, PdnC hydroxylates Trp7 to yield the final product **1**. OHPro = 4-hydroxyproline. Domains are abbreviated as C = condensation, A = adenylation, T = thiolation, E = epimerization, TE = thioesterase. Domains are colored according to their type or subtype.

The *pdn* BGC also harbors auxiliary genes, encoding an AraC family type regulator *pdnA* and an ABC transporter ATP-binding protein *pdnB* (Table S7). *pdnC* encodes a homologue of monodechloroaminopyrrolnitrin synthase (PrnB), a flavin-dependent enzyme classically involved in tryptophan metabolism and pyrrolnitrin biosynthesis,^47–50^ which is part of the heme-dependent dioxygenase superfamily.^47^ In ∼50% of all detected *pdn*-like BGCs (Figure S22), this gene is located directly upstream of the NRPS core gene. Given that paenidepsin A and some of its congeners contain one or two hydroxylated Trp residues, we predict this PrnB homologue to play a role in indole hydroxylation. Nonetheless, its function remains speculative, as the enzyme does not clade phylogenetically with Luz15, a recently characterized tryptophan 5-hydroxylase from *Actinomadura luzonensis* DSM 43766 that regioselectively hydroxylates free Trp at C-5 (Figure S23).^51^ This functional uncertainty warrants future biochemical characterization of PdnC. Furthermore, the *pdn* BGC includes *pdnE*, predicted to encode a chitosanase, previously associated with antifungal properties.^52^ Both *pdnC* and *pdnE* are found together in about 50% of all complete *pdn* BGCs, which may reflect functional modularity or evolutionary adaptation. Notably, while paenidepsin A contains 4-hydroxyproline, no gene encoding a proline 4-hydroxylase was identified within or near the *pdn* BGC. This suggests that proline hydroxylation is catalyzed by an enzyme encoded elsewhere in the genome, a situation commonly observed for certain translational or tailoring modifications.^27^ The availability of the nonproteinogenic building block citrulline could be explained as an intermediate of arginine biosynthesis.^53^

The *pdn* BGC from *Paenibacillus*sp. P3_m182_1 was reassembled *in silico* from multiple contigs, and its continuity was experimentally supported by a PCR-based gap closure strategy, utilizing primers designed for this purpose (Table S8 and Figure S24). The *pdn* BGC encodes two NRPS proteins, comprising a total of 12 modules, the first NRPS (PdnD1) having seven modules and the second NRPS (PdnD2) five modules (Figure S2). The predicted amino acids incorporated by the A domain align with the de novo sequencing of compound **7** (Figure S1). Analogous to the *pdn* BGC of *P. apiarius* MW-14, this NRPS system also contains E domains in the modules 7 and 9, which are predicted to incorporate a d-configured leucine and tryptophan, respectively. Another difference to *P. apiarius* MW-14’s BGC is the production of a peptide with proline instead of hydroxyproline, with a threonine residue instead of citrulline and asparagine as last amino acid instead of aspartic acid (Figure S1). The isolation of compound **7** in sufficient amounts for NMR experiments and Marfey’s analysis^54^ are required to confirm the presence of leucine and not isoleucine in the seventh position, as well as to analyze its stereochemistry (Figure S2).

### Diversity of the *pdn* BGC Family

We then performed a more comprehensive bioinformatic analysis of the *pdn* BGC to investigate the distribution of this lipopeptide gene cluster family. Initially, we constructed a phylogenetic tree of the fatty acid incorporating *C*_Starter_ domains of NRPS BGCs from the genus *Paenibacillus* (Figure S25). This tree contained three major clades formed by the *C*_Starter_ sequences of the known antibiotics polymyxins, fusaricidins and tridecaptins (Figure 5A).^7^ Additionally, there were multiple clades not associated with any previously characterized lipopeptides, highlighting the continued potential of *Paenibacillus* as a useful source of novel bioactive lipopeptides.^23^ The paenidepsin *C*_Starter_ sequences formed a distinct, previously uncharacterized clade that did not cluster within a larger group of sequences primarily associated with antibiotic biosynthesis, and thus consistent with the antifungal activity (Figure 5B).

**Figure 5:**
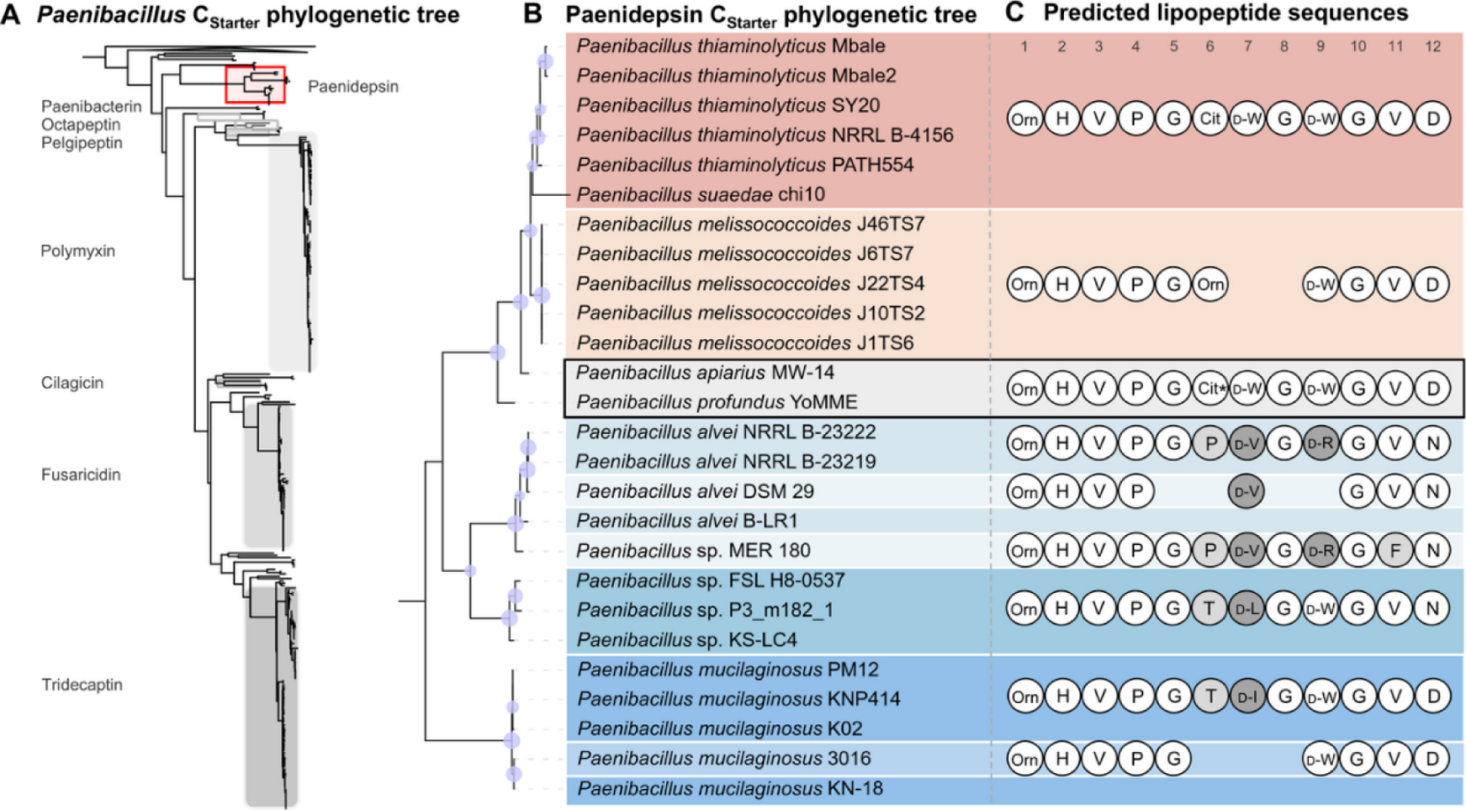
Genome mining for homologous *pdn* BGCs uncovers the putative structural diversity of paenidepsins. (A) Maximum-likelihood tree of *C*_Starter_ domain amino acid sequences from NRPS gene clusters within the genus *Paenibacillus*. The paenidepsin clade is highlighted in red and known lipopeptides clades are shown in gray. (B) Maximum-likelihood tree of the *C*_Starter_ domains of each complete *pdn* BGC. Bootstrap support above 60% is visualized by node sizes. Tree according to scale. (C) Putative peptide sequences of the pdn BGC for representative members of each clade are shown, with variant residues highlighted in gray.

Subsequently, we performed a more targeted genome mining with cblaster,^55^ which yielded 71 homologous gene clusters, all within the genus *Paenibacillus*, 25 of which were complete (Table S10 and Figure S22). Each identified BGC was further analyzed using antiSMASH,^56^ followed by adenylation domain substrate specificity prediction with PARAS^46^ to obtain the putative peptide sequences of the encoded lipopeptides. Although most of the predicted peptide sequences exhibit high similarity, variations in the number of biosynthetic modules result in differences in peptide length (8 to 12 amino acids) among the identified gene clusters (Figure 5C).

Interestingly, the N- and C-terminal residues of the predicted paenidepsin peptide sequences are almost identical, while there is more variation in the residues of the central region (Figure S26). We noticed that the d-Trp–Gly amino acid sequence is repeated at position 7–8 and 9–10 in most predicted peptide sequences, sometimes with variations at position 7. Therefore, we speculated that this repetitive feature may have arisen by duplication of the corresponding NRPS modules, a well-known driver in the evolution of NRPS biosynthetic genes.^57,58^ A nucleotide alignment of the modules M7-8 and M9-10 of the *pdn* BGC from *P. apiarius* MW-14 exhibited more than 90% identity (Figure S27). The high degree of sequence identity of these modules was also detected in all other *pdn* BGCs carrying this feature, leading us to hypothesize that all putative peptide sequences with 12 amino acids appear to result from the duplication of these two modules.

To explain the variation at position 7 of the peptide (Figure 5C), we performed a sliding window analysis to identify regions of high sequence divergence, possibly originating from recombination, another major driver in the diversification of NRPS compound families. Indeed, we observed high sequence divergence in the A domains of module 7 from different *pdn* gene clusters (Figure S28A). Similar patterns were also observed for residues at position 6 and 9 (Figure S28B,C). The A core domain is a known recombination hotspot, and its diversification has been correlated with the diversification of NRPS compound families.^57,59^ Accordingly, variation at position 7 of the peptide likely stems from the initial duplication of the modules for d-Trp and Gly, followed by recombination events, that led to different substrate specificities of module 7 (Figure 6).

**Figure 6:**
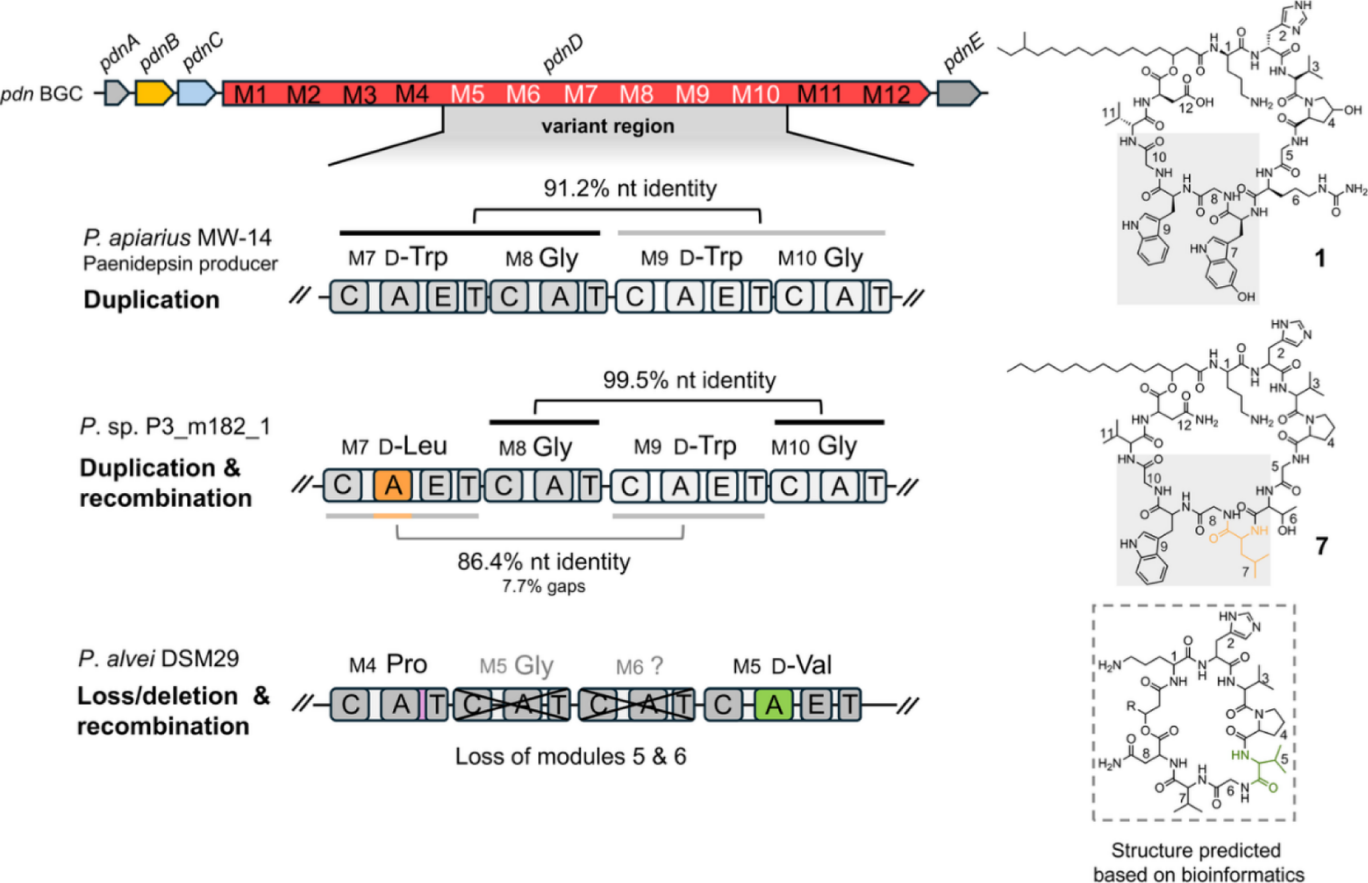
Potential events in the evolutionary diversification of the *pdn* BGC that led to the detected and predicted structural diversity of the paenidepsins from *P. apiarius* MW-14, *P.* sp. P3_m182_1 and *P. alvei* DSM 29. Domain-level comparisons of modules from the variable region of the *pdn* BGC and the putative evolutionary processes involved are shown and linked to the structures on the right.

Another observation was the reduced number of NRPS modules in some *pdn* BGCs, resulting in shorter predicted peptide sequences. The absence of some of these modules is presumably the result of loss or deletion of the respective modules, which we aimed to detect by performing a dot plot analysis. The corresponding plots clearly indicated sequence deletion of module 5 and 6 from an ancestral pdn BGC with 10 amino acids after the A-T linker sequence of module 4 (Figures 6 and S29). The loss of whole modules or domains has recently been recognized to be another driver of gene cluster diversification.^58^ Collectively, these observations suggest that the central region of the paenidepsins is subject to evolutionary diversification, while the terminal regions of the lipopeptide may serve a more essential functional role, and are hence more conserved.

The paenidepsins (compounds **1**–**7** and KB425796 A-K lipopeptides^32,33^ ) lack high positive charge, and anti-Gram-negative activity, which are typical for several well-known *Paenibacillus* cyclic LP families.^17,60^ Their structural framework also differs from known noncationic LPs from *Paenibacillus* such as fusaricidins,^20^ paenilipoheptins,^21^ and tridecaptins^6^ in ring size, fatty acid and amino acid sequence. Furthermore, to our knowledge, no structurally similar cyclic LPs have been isolated from other bacterial genera,^3,17^ supported by searches in relevant databases (NORINE,^61^ CAS SciFinder,^62^ The Natural Products Atlas^63^). In addition, analysis of the nonfragmented *pdn* BGCs (Figure S21) using antiSMASH 8.0^45^ did not show significant homology to any characterized BGCs of known LPs with respect to length and nature of predicted amino acid sequence, leading to the conclusion that **1**–**7** and the KB425796 LPs cannot be classified into the current known lipopeptide families. Taken together, our analyses strongly support the placement of **1**–**7** and KB425796 A-K as a new lipopeptide family, for which we propose the name paenidepsins. This family comprises cyclic lipododecapeptides produced by *Paenibacillus* with a largely conserved sequence (see Table S2 and Figure S25) and a β-hydroxy fatty acid with a chain length of 15–16 carbon atoms, forming an intramolecular ester bond with the terminal Asp/Asn.

## Conclusions

We isolated a new cyclic lipododecapeptide with antifungal activity from *P. apiarius* MW-14. Through literature and genome mining, we were able to identify and describe a previously unreported family of nonribosomal lipopeptides and their associated BGCs in *Paenibacillus* spp., and to propose a biosynthetic model for this compound family. This discovery not only enhances our understanding of the chemical and genetic diversity within the *Paenibacillus* genus, but also holds promise for agricultural and pharmaceutical applications.

Various strains of the genus *Paenibacillus* that contain the paenidepsin BGC have been recognized as beneficial across diverse agricultural settings or demonstrate strong associations with plant systems, which suggests that the peptides may play an important ecological role in these environments.^13,64^ Taken together, our findings set the stage for both biosynthetic investigations and the rational exploration of paenidepsin-like compounds for antifungal applications in medicine and agriculture.

## Experimental Section

### General Experimental Procedures

NMR spectra were recorded on a Bruker Ascend 600 NMR spectrometer operating at 600 MHz (^1^H) and 150 MHz (^13^C) or on a Bruker Avance III ID Ascend 700 MHz spectrometer operating at 700 MHz (^1^H) and 175 MHz (^13^C) using MeOD as solvent from Deutero GmbH (99.96% D). NMR spectra were processed using Bruker Topspin version 1.3 software and MestReNova 14.2.1. Spectra were referenced to residual solvent signals with resonances at δ_H/C_ 3.31/49.0 for MeOD. High-resolution LC–MS/MS data were recorded on an Agilent 6550 iFunnel Q-TOF mass spectrometer with a Dual AJS ESI source coupled to an Agilent 1100 Series HPLC system. The separation was carried out on an Atlantis T3 C18 5 μm 4.6 × 50 mm column (Waters) at 20 °C. MS data were acquired over a range from 100 to 3000 *m*/*z* in positive mode. Auto MS/MS fragmentation was performed with rising collision energy (28–115 keV) over a gradient from *m*/*z* 500 to 2000, with a frequency of 6 Hz for all ions above a threshold of 100. HPLC begins with 90% of H_2_O containing 0.1% formic acid. The gradient starts after 2 min to 100% acetonitrile (0.1% formic acid) in 12 min. A 20 μL amount of a 0.5 mg/mL sample solution (MeOH) was injected at a flow of 0.6 mL/min. Data Analysis was performed using Agilent MassHunter Workstation Qualitative Analysis version 10.0 (Build: 10.0.10305.0).

For extract screening and relative quantification, additional LC–UV/MS measurements were performed using a Waters 2695 separation module coupled to a Waters 996 photodiode array (PDA) detector and a Waters QDa mass detector equipped with an electrospray ionization (ESI) source. Chromatographic separations were achieved on a Waters XBridge Shield RP18 column (100 × 2.1 mm, 3.5 μm particle size) maintained at 25 °C. The mobile phase consisted of solvent A (acetonitrile/water 5:95, v/v, containing 5 mM ammonium acetate and 40 μL acetic acid per liter) and solvent B (acetonitrile/water 95:5, v/v, with the same additives). The gradient elution program was as follows: 80% A to 0% A over 20 min, followed by a 10 min hold at 100% B, at a flow rate of 0.3 mL/min. UV detection was performed at 220 and 280 nm. Mass spectrometric data were acquired in positive ion mode over an *m*/*z* range of 140–1250.

### Organism Collection and Identification

*Bacillus subtilis* JH642 + *sfp* was provided by Huimin Zhao (University of Illinois Urbana–Champaign, IL, US). *E. rubrum* DSMZ 62631 was purchased from the German Collection of Microorganisms and Cell Cultures (DSMZ, Germany). *P. apiarius* MW-14 was isolated from the rhizosphere of *Triticum aestivum* in the Isfahan Province, Iran (NCBI BioSample accession: SAMN17833047).

### Coculture Growth Conditions

Small-scale cultivations for HRMS extract generation were performed in TSB medium. TSB medium was prepared using 5 g/L NaCl, 2.5 g/L K_2_HPO_4_, 2.5 g/L glucose, 17 g/L casein peptone (pancreatic) and 3 g/L soya peptone (papain digest). For the precultures, *Bacillus subtilis* JH642 + *sfp* was grown on Mueller–Hinton agar, while *E. rubrum* DSMZ 62631 was cultivated on a specialized agar medium. Mueller–Hinton agar was prepared using 2 g/L beef extract, 17.5 g/L casein hydrolysate, 1.5 g/L starch and 17 g/L agar. *Eurotium*’s specialized agar medium was prepared using 400 g/L saccharose, 20 g/L malt extract, 5 g/L yeast extract and 20 g/L agar.

For each condition, 100 × 500 mL of TSB medium were inoculated with either *P. apiarius* MW-14 or *Paenibacillus* sp. P3_m182_1 for both monoculture and coculture experiments.^28^

Precultures of *Paenibacillus* spp. were grown for 1 day at 30 °C and 200 rpm, while *Bacillus subtilis* JH642 + *sfp* was grown on Mueller–Hinton agar for 2 days at 30 °C, and *E. rubrum* DSMZ 62631 was grown on a specialized agar for 7 days at 25 °C. The inoculation of *B. subtilis* was performed using an aqueous suspension, while *E. rubrum* was inoculated using an aqueous solution containing 0.05% Tween 80.

Cocultures were inoculated in a 1:1 ratio, with *B. subtilis* or *E. rubrum* introduced after 4 days of *Paenibacillus* spp. growth, which was considered “day 0”. Cocultures with *E. rubrum* were incubated without shaking, following a previously described method.^65^ Samples were extracted with *n*-butanol at 1 h, 4, 7, 10, and 14 days postinoculation. The crude extracts were afterward prepared for HPLC-MS/MS analysis by dilution to a concentration of 0.5 mg/mL with methanol.

### Cultivation of *P. apiarius* MW-14 and Isolation of Paenidepsin A

For the large-scale cultivation, *P. apiarius* MW-14 was grown in 2 L of TSB. TSB medium was prepared using 5 g/L NaCl, 2.5 g/L K_2_HPO_4_, 2.5 g/L glucose, 17 g/L casein peptone (pancreatic) and 3 g/L soya peptone (papain digest). The bacterial pellet was separated from the supernatant by centrifugation (3000 rpm, 15 min) and extracted 3× with *n*-butanol by shaking to yield approximately 2 g of crude extract.

Each purification step was accompanied by MS analysis of the fractions. The crude material was fractionated on a Reveleris C18 flash column (40 g, 40 μm). A stepwise gradient solvent system of decreasing polarity at a flow rate of 40 mL/min was used starting with 80/20 H_2_O/MeOH for 6.7 min, then changing to 60/40 H_2_O/MeOH within 0.4 min and held again for 4.4 min. The gradient was changed then within 0.4 min to 40/60 H_2_O/MeOH and held for 4.4 min, then within 0.4 min to 30/70 H_2_O/MeOH, held for 4.4 min, then within 0.4 min to 20/80 H_2_O/MeOH and held for 4.4 min, then within 0.4 min to 15/85 H_2_O/MeOH and then within 0.4 min to 10/90 H_2_O/MeOH. Finally, the gradient was changed within 0.4 min to 100% MeOH and held for additional 21.7 min. According to the measured evaporative light scattering detector and UV signals, a paenidepsin-A-containing fraction was collected at 30 min. Next purification was done by HPLC with a semipreparative Phenomenex Luna C18(2) column (250 × 10 mm, 5 μm) using a stepwise gradient with a H_2_O and acetonitrile (MeCN) solution both containing 0.1% TFA starting with 60/40 H_2_O/MeCN for 15 min, then changed to 50/50 H_2_O/MeCN and held for 15 min, then to 40/60 H_2_O/MeCN for 15 min. The gradient was finally changed to 100% MeCN and held for additional 15 min (flow rate 3.0 mL/min). The paenidepsin-A-containing fraction was collected at 32 min. Final purification was done by HPLC with a Phenomenex Kinetex C18 column (250 × 4.6 mm, 5 μm) using an isocratic elution with 55/45 H_2_O/MeCN (flow rate 0.7 mL/min). Pure paenidepsin A was isolated as a white powder (Paenidepsin A: *t*_R_: 23,4 min, 0.5 mg).

### Paenidepsin A (**1**)

White, solid; ^1^H and ^13^C NMR (MeOD-*d*_4_) Table S1; (+)-HR-ESI-MS *m*/*z* 1662.8956 [M + H]^+^ ([M + H]^+^ calcd for C_81_H_119_N_19_O_19_, 1662.9003).

### Bioinformatic Analysis

The genome sequence of *P. apiarius* MW-14 was obtained from the NCBI BioSample (SAMN17833047)^30^ and that of *Paenibacillus* sp. strain P3_m182_1 was sequenced at Eurofins Genomics (Ebersberg, Germany). Both genomes were analyzed using antiSMASH 8.0 to identify and characterize the BGCs, particularly those encoding NRPSs.^45^

To identify homologous BGCs, BLAST searches were performed using NRPS protein sequences, and cblaster was employed with the full amino acid set of the *pdn* BGC.^55^ Identified hits were analyzed for completeness with antiSMASH 7.0,^56^ and A domain substrate specificities were predicted using PARAS to obtain putative peptide sequences of all homologous pdn BGCs.^46^ Homologous *pdn*-like BGCs were compared and visualized using clinker.^55^

For comparative analysis, protein sequences encoded within the gene clusters were aligned with their closest homologues from NCBI or UniProtKB/Swiss-Prot using UniProt’s pairwise sequence alignment tool (https://www.uniprot.org/align). The percentage of sequence similarity and identity was calculated using the SMS2 Sequence Identity and Similarity Tool (https://www.bioinformatics.org/sms2/ident_sim.html).^66^ Dot plot analysis of selected *pdn* BGCs was performed using VectorBuilder’s Sequence Plot Tool (https://en.vectorbuilder.com/tool/sequence-dot-plot.html).

Variations in the predicted peptide sequences were subsequently related to nucleotide sequence divergence in the genes that encode the NRPS modules as described by Baunach et al.^59^ For this, nucleotide sequence pairs of different modules were aligned in MEGA11 ^67^ using the MUSCLE alignment algorithm and then analyzed using a sliding window analysis in DnaSP6 ^68^ to obtain π values, that represent the average number of nucleotide differences per site between two sequences. The sliding window had a width of 300 nt and a step size of 150 nt. Sequence similarity and putative recombination breakpoints were visualized by mapping π values against window midpoints.

For the phylogenetic analysis of starter condensation domain (*C*_Starter_) amino acid sequences, all publicly available genomes of the genus *Paenibacillus* (312) with the assembly level complete were downloaded on 28.02.2025. All genomes were subsequently analyzed with antiSMASH v7.0.^56^ For the construction of the *C*_Starter_ phylogenetic tree, all *C*_Starter_ amino acid sequences from NRPS and NRPS-like cluster were extracted from each antiSMASH output file using a custom python script (Figure S30). Briefly, this script loads the antiSMASH output file in GenBank format and searches for NRPS and NRPS-like gene clusters that contain *C*_Starter_ domains. *C*_Starter_ amino acid sequences were extracted into a separate fasta file if the respective BGC encodes more than 5 A domains. The final data set consisted of 419 *C*_Starter_ amino acid sequences, that were used to build a multiple sequence alignment using the MUSCLE alignment algorithm in MEGA11 after manual curation.^67^ This alignment was used to create a maximum-likelihood tree in MEGA11 that was visualized in iTOL.^69^

For phylogenetic analysis of PdnC and related enzymes such as those from the heme-dependent aromatic oxygenase (HDAO) superfamily and other Trp-modifying enzymes, selected amino acid sequences thereof were retrieved from GenBank and antiSMASH outputs. Sequences were aligned using MUSCLE in MEGA11 ^67^ manually curated, and a neighbor-joining phylogenetic tree was constructed with 1000 bootstrap replicates. The resulting tree was visualized using iTOL.^69^

### Classic Molecular Networking

LCMS files were exported to centroid format (mzML format) using the MSConvert tool of the software ProteoWizard (version 3.0.22,257-7de06d7).^70^ A molecular network was created using the online workflow (https://ccms-ucsd.github.io/GNPSDocumentation/) on the GNPS Web site (http://gnps.ucsd.edu).^34^ The data was filtered by removing all MS/MS fragment ions within ±17 Da of the precursor *m*/*z*. MS/MS spectra were window filtered by choosing only the top 6 fragment ions in the ±50 Da window throughout the spectrum. The precursor ion mass tolerance was set to 0.02 Da and a MS/MS fragment ion tolerance of 0.02 Da. A network was then created where edges were filtered to have a cosine score above 0.7 and more than 6 matched peaks. Further, edges between two nodes were kept in the network if and only if each of the nodes appeared in each other’s respective top 10 most similar nodes. Finally, the maximum size of a molecular family was set to 100, and the lowest scoring edges were removed from molecular families until the molecular family size was below this threshold. The spectra in the network were then searched against GNPS’ spectral libraries and suspect libraries.^71^ The library spectra were filtered in the same manner as the input data. Dereplication was performed with the DEREPLICATOR and DEREPLICATOR+. All matches kept between network spectra and library spectra were required to have a score above 0.7 and at least 6 matched peaks. Results were visualized in Cytoscape 3.10.2 (https://cytoscape.org/) using the solid style layout. Nodes represent parent masses, while the origin of nodes is presented as a pie chart (blue: produced by *P. apiarius* MW-14, yellow: produced by *P. apiarius* MW-14 cocultured with *B. subtilis* JH642 + *sfp*, gray: produced by *P. apiarius* MW-14 cocultured with *E. rubrum* DSMZ 62631, red: produced by *Paenibacillus* sp. P3_m182_1, green: produced by *Paenibacillus* sp. P3_m182_1 cocultured with *B. subtilis* JH642 + *sfp*, orange: produced by *Paenibacillus* sp. P3_m182_1 cocultured with *E. rubrum* DSMZ 62631), the size of the node is proportional to the number of spectra, and edge thickness corresponds to cosine score.

### Bioactivity Assays

The antifungal activity of paenidepsin A and reference compounds fluconazole (Thermo Fisher Scientific Inc., Darmstadt, Germany), amphotericin B and micafungin (Cayman Chemicals, Michigan, USA) was evaluated against *C. albicans* strains I-11301, I-11134,^72^ and *A. fumigatus* DSM819 (DSMZ, Braunschweig, Germany). To determine the broth dilution minimum inhibitory concentrations (MICs) and minimum effective concentrations (MECs) we followed the EUCAST recommendations for conidia forming molds (EUCAST E.DEF 9.4 March 2022)^73^ and yeasts (EUCAST E.DEF 7.4 October 2023).^74^ Advanced RPMI 1640 (1×, with nonessential amino acids and 110 mg/L sodium pyruvate, without l-Glutamine) (Thermo Fisher Scientific Inc., Darmstadt, Germany) medium supplemented with glucose in a final concentration of 2% (Merck KGaA, Darmstadt, Germany) and MOPS (Carl Roth GmbH + Co. KG, Karlsruhe, Germany) at a final concentration of 0.165 mol/L, pH 7.0 (for pH stability) was used for cultivation of fungi. Paenidepsin A was dissolved in MeOH, while reference compounds were solubilized in DMSO (Merck KGaA, Darmstadt, Germany; stock concentrations 10 mg/mL), aliquoted and stored at −80 °C.

Inoculum suspensions were prepared from fresh, mature (2- to 5 day-old) *A. fumigatus* subcultured on Sabouraud-Dextrose agar plates (Thermo Fisher Diagnostics, Wesel, Germany) and incubated at 30 °C. The colonies were covered with 5 mL sterile water supplemented with 0.1% Tween 20 (Carl Roth GmbH + Co. KG, Karlsruhe, Germany) and carefully rubbed with a sterile cotton swab. After transfer to a sterile tube, the suspension was vortexed shortly (15 s) and examined for the presence of hyphae or clumps. The inoculum was filtered (pore diameter = 10 μm) and adjusted to a concentration equivalent to McFarland 0.5 ((2–5) × 10^6^ cfu/mL).

100 μL medium was added to each well of a polystyrene 96-well microtiter plate with flat-bottom wells (Greiner Bio-One, Kremsmuenster, Austria) in which a serial dilution of the antifungal agents was prepared with starting concentrations ranging from 16 μg/mL to 1024 μg/mL.

Each well was then inoculated with 100 μL conidial medium suspension (diluted 1:10 in medium to adjust the final inoculum in the plate to 1 × 10^5^ to 2.5 × 10^5^ cfu/mL). Growth and negative controls were included for reference. The plate was incubated at 30 °C without agitation. The MEC, defined as the lowest concentration causing a substantial reduction in fungal growth and/or abnormal, branched and short hyphae (e.g., rosettes described for micafungin) in comparison to the long, unbranched hyphal growth in the growth control, was determined after 17 h postincubation. MICs, defined as the lowest concentration required to completely inhibit visual growth, were evaluated after 48 h.

For the viability count, 10 μL of inoculum suspension was diluted in 2 mL of sterile distilled water supplemented with 0.1% Tween 20 and vortexed. 100 μL of that mixture was spread on a Sabouraud-Dextrose agar plate and incubated (24–48 h). 100–250 colonies are expected from an acceptable test suspension.

*C. albicans* was grown on Sabouraud-Dextrose agar plates and incubated at 37 °C for 18–24 h (until colonies were >1 mm). Approximately five colonies were picked and suspended in 3 mL sterile water. After vortexing, this solution was adjusted to McFarland 0.5 and diluted 1:10 (1–5 × 10^5^ cfu/mL). All other steps were performed as described above. MICs were determined by visual inspection after 24 h of incubation and defined as the lowest concentration of the drug that caused significant growth reduction to meet EUCAST criteria. Ten μL of the inoculum suspension were plated on Sabouraud-Dextrose or Mueller–Hinton agar (Thermo Fisher Diagnostics, Wesel, Germany), incubated at 37 °C overnight and checked for purity.

Drug-induced morphological changes were examined using wet mount microscopy. Phase contrast images were acquired on a AxioObserver Z1 microscope equipped with a Colibri 5/7 LED, a Plan-Apochromat 40*x*/0.65 Ph 2 M27 objective, and a Axiocam 820 mono camera using the Zen Blue 2.0 software (Carl Zeiss AG, Oberkochen, Germany) and analyzed using ImageJ win64 software.^75^ To illustrate the micromorphological differences, tiny, loose pieces of hyphae were obtained from 96-well plates using a sterile pipet tip and placed directly onto a clean glass slide. A small drop of sterile water (5–10 μL) was added to replace residual medium. After this wash step, new water was added and the coverslip was added carefully to avoid air bubbles and to prevent crushing of the hyphae. Samples were kept moist at all times.

Antibacterial activity was evaluated against *S. aureus* HG001,^76^ and *P. aeruginosa* PAO1 as follows.^77^ MICs were determined by standard broth microdilution according to CLSI guidelines^78^ in polystyrene microtiter plates with U-bottom (Greiner Bio-One, Kremsmuenster, Austria) using cation-adjusted Mueller–Hinton broth (Thermo Fisher Diagnostics, Wesel, Germany). MICs were determined as the lowest concentrations where no visible growth was observed.

### Agar Overlay Assay

Briefly, *Paenibacillus*sp.P3_m182_1 and *P. apiarius* MW14 were grown overnight and then used to inoculate TSB medium for production of antibacterial metabolites (1:100 dilution). After 4 days of incubation, increasing amounts of these cultures (from 0.5 μL up to 12 μL) were spotted on the surface of a Petri dish containing 15 mL of TSA and incubated overnight at 30 °C, before being covered with 10 mL soft Iso-Sensitest agar (Oxoid Iso-Sensitest broth 23.4 g/L, Carl Roth, 0.8% agar) containing an inoculum of either *S. aureus* ATCC 29213 or *S. aureus* HG 001 (100 μL overnight culture). The plates were incubated at 30 °C and the inhibition zones were evaluated after 24 h.

## Supplementary Material





## Data Availability

The assembled genome sequence of *Paenibacillus* sp. strain P3_m182_1 was deposited in GenBank under accession number JBPULM000000000 (BioSample: SAMN49993483, BioProject: PRJNA1292205). The version described in this paper is version JBPULM010000000. The GNPS molecular networking job is accessible via the link https://gnps.ucsd.edu/ProteoSAFe/status.jsp?task=2782acc9d8f648e2ab3e8556d4b799e0. HR-ESI-LC-MS/MS data were deposited in MassIVE (MSV000098314). The *pdn* BGC data was deposited in the MiBIG database under accession number BGC0003181. NMR spectra of (**1**) are included in the Supporting Information.

## Notes

Analysis of the specificity-conferring residues in the *pdn* and closely related BGCs. Production curve of **1** by *P. apiarius* MW-14 in TSB. HR-ESI-LC-MS/MS spectra of **1**–**7**; NMR spectra and table of **1**, light microscopy images of bioactivity results, phylogenetic trees, Python script and further bioinformatic analyses (PDF)
Raw NMR data (FIDs) (ZIP)
