# Supplementary material for: Paenidepsins are a Family of Lipopeptides from Paenibacillus
Source: J Nat Prod. 2026 Mar 3;89(3):966–79. doi: 10.1021/acs.jnatprod.5c01579 (PMC13036774; doi:10.1021/acs.jnatprod.5c01579)
Supplement: Supplementary file 1 [file np5c01579_si_001.pdf]

Supporting Information

Paenidepsins are a Family of Lipopeptides from *Paenibacillus*

Daniel Torres-Püschel[1], Lukas Zimmer[1,2], Stefan Kehraus[1], Aia Ali Abdelrahman[3], Tanja Schneider[3,4], Anna Müller[3], Aurélien Carlier[5], Max Crüsemann[1,2*]

1 Institute of Pharmaceutical Biology, University of Bonn, 53115 Bonn, Germany

2 Institute of Pharmaceutical Biology, Goethe-University Frankfurt, 60438 Frankfurt, Germany

3 Institute for Pharmaceutical Microbiology, University of Bonn, University Hospital Bonn, 53115 Bonn, Germany

4 German Center for Infection Research (DZIF), partner site Bonn-Cologne, 53127 Bonn, Germany.

5 LIPME, Université de Toulouse, INRAE, CNRS, Castanet-Tolosan 31326, France

*Corresponding author: Email: cruesemann@em.uni-frankfurt.de

Table of Contents.

Supplementary Tables **Page S5**

Table S1. ^{1}H and ^{13}C NMR Spectroscopic Data for paenidepsin A (**1**), in Methanol-d_4 (^{1}H: 600 MHz; ^{13}C: 150 MHz).

Table S2. Comparison of peptide sequences of paenidepsins and KB425796A-K.

Table S3. Antimicrobial activity (MICs in µg/ml) of Paenidepsin A (**1**).

Table S4. Analysis of the specificity-conferring residues in the PdnD adenylation domains.

Table S5. Analysis of the specificity-conferring residues in the adenylation domains of BGCs producing hydroxyproline-containing compounds.

Table S6. Analysis of the specificity-conferring residues in the adenylation domains of BGCs producing citrulline-containing compounds.

Table S7. Deduced proteins encoded in *Paenibacillus* sp. P3_m182_1's *pdn* BGC and their putative functions.

Table S8. Primers used in this study.

Table S9. Deduced proteins encoded in *Paenibacillus apiarius* MW-14's *pdn* BGC and their putative functions.

Table S10. Genome mining for homologous *pdn* BGCs.

Supplementary Figures **Page S17**

Figure S1. MS^2 spectrum and chemical structure of compound **7** (m/z: 736.94).

Figure S2. *pdn* BGC of *Paenibacilllus* sp. P3_m182_1.

Figure S3. Comparative Analysis of Peptide Production in *Paenibacilllus* sp. P3_m182_1 and *Paenibacillus apiarius* MW-14.

Figure S4. Chemical structure and MS^2 spectrum of paenidepsin A (**1**) (*m/z* 1662.89).

Figure S5. ^{1}H NMR spectrum of paenidepsin A (**1**) in methyl alcohol-d_4 (600 MHz).

Figure S6. ^{13}C NMR spectrum of paenidepsin A (**1**) in methyl alcohol-d_4 (150 MHz).

Figure S7. DEPT-135 NMR spectrum of paenidepsin A (**1**) in methyl alcohol-d_4 (150 MHz).

Figure S8. ^{1}H-^{1}H COSY NMR spectrum of paenidepsin A (**1**) in methyl alcohol-d_4 (150 MHz).

Figure S9. ^{1}H-^{13}C HSQC NMR spectrum of paenidepsin A (**1**) in methyl alcohol-d_4 (150 MHz).

Figure S10. ^{1}H-^{13}C HMBC NMR spectrum of paenidepsin A (**1**) in methyl alcohol-d_4 (150 MHz).

Figure S11. ^{1}H-^{1}H ROESY NMR spectrum of paenidepsin A (**1**) in methyl alcohol-d_4 (150 MHz).

Figure S12. 1H-1H TOCSY NMR spectrum of paenidepsin A (**1**) in methyl alcohol-d_4 (600 MHz).

Figure S13. Molecular network of the metabolomes of *Paenibacillus* spp.

Figure S14. Molecular cluster of paenidepsin A (determined by MS^2) from the molecular network.

Figure S15. Proposed chemical structure and MS^2 spectrum of paenidepsin B (**2**) (m/z: 839.95).

Figure S16. Proposed chemical structure and MS^2 spectrum of paenidepsin C (**3**) (*m/z* 824.95).

Figure S17. Proposed chemical structure and MS^2 spectrum of paenidepsin D (**4**) (*m/z* 823.96).

Figure S18. Proposed chemical structure and MS^2 spectrum of paenidepsin E (**5**) (*m/z* 817.94).

Figure S19. Proposed chemical structure and MS^2 spectrum of paenidepsin F (**6**) (*m/z* 809.94).

Figure S20. Results of antibacterial overlay assays of *Paenibacillus* sp. P3_m182_1 and *Paenibacillus apiarius* MW14 against *S. aureus* isolates

Figure S21. Light microscopy images of *A. fumigatus* DSM819 cells treated with paenidepsin A and micafungin alone or in combination.

Figure S22. Comparison of genes and encoded proteins in the *pdn* BGCs.

Figure S23. Phylogenetic tree of HDAOs and PdnC including Luz15.

Figure S24. Gap closure of *Paenibacillus.* sp. P3_m182_1*a pdn* BGC.

Figure S25. Phylogenetic tree using $C_{Starter}$ domains of whole-genome sequences of *Paenibacillus* spp.

Figure S25. Sequence logo plot of predicted peptide sequences from complete *pdn* BGCs.

Figure S27. Emboss Needle Alignment of modules from *pdn* BGC of *P. apiarius* MW-14.

Figure S28. Sliding window analysis of putative recombination spots in different *pdn* BGC encoding *Paenibacillus* strains.

Figure S29. Dot plot analysis of the nucleotide sequence of the NRPS genes in *pdn* BGCs.

Figure S30. Python script's scheme for extracting $C_{Starter}$ amino acid sequences from NRPS and NRPS-like BGCs.

Supplementary references **Page S50**

Supplementary Tables

Table S1. ^{1}H and ^{13}C NMR spectroscopic data for paenidepsin A (**1**), in methanol-d_4 (^{1}H: 600 MHz; ^{13}C: 150 MHz).

Residue	no. C/H	δ_H (*J* in Hz)	δ_C, mult.
**Acyl**	1		n.d.
	2	2.29, 2.91	41.2
	3	5.13, m	72.9
	4	1.58	33.6
	5	1.28-1.32[a]	30.3-30.9[b]
	6	1.28-1.32[a]	30.3-30.9[b]
	7	1.28-1.32[a]	30.3-30.9[b]
	8	1.28-1.32[a]	30.3-30.9[b]
	9	1.28-1.32[a]	30.3-30.9[b]
	10	1.28-1.32[a]	30.3-30.9[b]
	11	1.28-1.32[a]	30.3-30.9[b]
	12	1.28-1.32[a]	30.3-30.9[b]
	13	1.12, 1.32	37.8
	14	1.32	35.7
	15	1.28-1.32[a]	30.3-30.9[b]
	16	0.90	11.8
	17	0.88	19.8
**Asp12**	18		n.d.
	19	4.83	50.3
	20	2.50, 3.11	41.0
	21		177.4
**Val11**	22		n.d.
	23	4.94	58.2
	24	1.91	33.3
	25	0.84, d, 6.60 Hz	19.1
	26	0.87	19.4
**Gly10**	27		n.d.
	28	3.09, 4.08	43.0
**Trp9**	29		173.3
	30	5.05	55.3
	31	3.00, 3.32	29.1
	32		109.8
	33		129.0
	34	7.51, d, 7.70 Hz	119.4
	35	6.99, t, 7.70 Hz	119.6
	36	7.06	122.1
	37	7.32, d, 8.20 Hz	112.2
	38		137.7

	39	7.18, s	125.3
**Gly8**	40		n.d.
	41	3.85, 4.43	43.8
**OHTrp7**	42		n.d.
	43	4.71, dd, 4.10, 10.40 Hz	56.4
	44	3.06, 3.48	27.9
	45		129.0
	46		110.3
	47	7.06	103.5
	48		151.4
	49	6.73, dd, 1.90, 8.20 Hz	112.7
	50	7.24, d, 8.20 Hz	112.9
	51		133.0
	52	7.16, s	125.5
**Cit6**	53		174.4
	54	4.13	55.8
	55	1.57	28.8
	56	1.03, 1.23	27.0
	57	2.90, 2.99	40.4
	58		162.1
**Gly5**	59		n.d.
	60	3.78, 4.12	43.6
**OHPro4**	61		n.d.
	62	4.62, br t, 7.70 Hz	60.0
	63	2.05, 2.20	39.3
	64	4.49, m	70.5
	65	3.77, 3.85	56.8
**Val3**	66		171.5
	67	4.36, d, 10.40 Hz	57.6
	68	1.82	32.0
	69	0.86	19.6
	70	1.02, br d, 6.60 Hz	19.7
**His2**	71		172.8
	72	4.81	55.4
	73	2.93, 3.08	n.d.
	74		n.d.
	75	6.95, br s	117.7
	76	7.61, br s	135.8
**Orn1**	77		n.d.
	78	4.82	52.4
	79	1.77, 2.03	31.2
	80	1.63, 1.83	23.4
	81	2.87, 2.93	40.4

a,b: resonances are overlapped; n.d.: not detected

Table S2. Comparison of peptide sequences of paenidepsins and KB425796A-K.[1,2]

Compound	Peptide sequence											Source organism
	**1**	**2**	**3**	**4**	**5**	**6**	**7**	**8**	**9**	**10**	**11**	**12**	
**Paenidepsin A, D, F**	Orn	His	Val	OHPro	Gly	Cit	OHTrp	Gly	Trp	Gly	Val	Asp	***Paenibacillus apiarius* MW-14**
**Paenidepsin B, C**	Orn	His	Val	OHPro	Gly	Cit	OHTrp	Gly	OHTrp	Gly	Val	Asp	
**Paenidepsin E, G**	Orn	His	Val	OHPro	Gly	Cit	OHTrp	Gly	Trp	Gly	Val	Asp	
**KB425796-A, B, C, F, G, H, I, J**	Orn	His	Val	Pro	Gly	Cit	OHTrp	Gly	Trp	Gly	Val	Asp	***Paenibacillus* sp. 530603**
**KB425796-D, E**	Orn	His	Val	Pro	Gly	Cit	OHTrp	Gly	OHTrp	Gly	Val	Asp	
**KB425796-K**	Orn	His	Val	Pro	Gly	Cit	Trp	Gly	Trp	Gly	Val	Asp	

Orn = ornithine, OHPro = 4-hydroxyproline, Cit = citrulline, OHTrp = 5-hydroxytryptophan.

Table S3. Antimicrobial activity (MICs and MECs in µg/mL) of paenidepsin A (**1**).

	MIC [µg/mL]		
	paenidepsin A	vancomycin	gentamicin
Staphylococcus aureus HG001	>64	1	n.d.
Pseudomonas aeruginosa PAO1	>64	n.d.	0.25

	MIC [µg/mL]				
	paenidepsin A	+ micafungin[1]	fluconazole	amphotericin B	micafungin
Candida albicans I-11301	>64	8	1	0.25	0.25
Candida albicans I-11134	>64	>64	0.5	0.5	0.125
Aspergillus fumigatus DSM 819	>64	>64	1024	0.0625	>32

	MEC [µg/mL]			
	paenidepsin A	fluconazole	amphotericin B	micafungin
Aspergillus fumigatus DSM 819	2	>256	n.d.	0.015625

n.d.: not detected, [1] in presence of 0.05 µg/mL micafungin

Table S4. Analysis of the specificity-conferring residues in the PdnD adenylation domains.

Module	Substrate recognition sequence	Corresponding amino acid in paenidepsin A	Predicted amino acid	PARAS Score
PdnD-m1	DVGEIGSID	Orn	Orn	0.857
PdnD-m2	DSTQVAEVV	His	His	0.180
PdnD-m3	DAFFIGGTF	Val	Val	0.696
PdnD-m4	DAQWIAHVA	4-OHPro	Pro	0.415
PdnD-m5	DITQLGMVW	Gly	Gly	0.637
PdnD-m6	DVENIGCVD	Cit	Orn	0.234
PdnD-m7	DATQVGEVV	5-OHTrp	Trp	0.281
PdnD-m8	DITQLGMVW	Gly	Gly	0.637
PdnD-m9	DATQVGEVV	Trp	Trp	0.281
PdnD-m10	DIAQLGMVW	Gly	Gly	0.826
PdnD-m11	DAFFIGGTF	Val	Val	0.565
PdnD-m12	DLTKVGHIG	Asp	Asp	0.823

Table S5. Analysis of the specificity-conferring residues in the adenylation domains of BGCs producing hydroxyproline-containing compounds.

Organism	BGC/Compound name	Module	Substrate recognition sequence	Corresponding amino acid in compound	Predicted amino acid	PARAS Score
Streptomyces canus ATCC 12646	BGC0001406/telomycin[3]	Tlo21-m7	DVQYAAHVV	*trans*-3-OHPro	Pro	0.996
		Tlo22-m11	DVQFVSQVM	*cis*-3-OHPro	Pro	0.819
Streptomyces griseoviridis NRRL 2427	BGC0000459/etamycin (viridogrisein)[4]	SgvD3-m1	DPLYVALVV	D-4-OHPro	(4*R*)-OHPro	0.620
Glarea lozoyensis ATCC 74030	BGC0001035/pneumocandin[5]	GloA-m3	DVSSATTVC	L-4-OHPro	(4*R*)-OHPro	0.889
		GloA-m6	DNTMITAMS	3-OH-4-Me-Pro (Pneumocandin A0)/3-OHPro (Pneumocandin B0)	(3*S*,4*S*)-3-OH-4-Me-Pro	0.966
Aspergillus nidulans NRRL 8112	BGC0001371.4/echinocandin B[6]	AniA-m3	DVSSATTVC	L-4-OHPro	(4*R*)-OHPro	0.966
		AniA-m6	DNTMITAMS	3-OH-4-Me-L-Pro	(3*S*,4*S*)-3-OH-4-Me-Pro	0.978

Table S6. Analysis of the specificity-conferring residues in the adenylation domains of BGCs producing citrulline-containing compounds.

Organism	BGC/Compound name	Module	Substrate recognition sequence	Corresponding amino acid in compound	Predicted amino acid	PARAS Score
Streptomyces scabiei 87.22	BGC0002367/rotihibins[7]	RthA-m1	DVWHSGSVT	D-Cit	Cit	0.615
Xenorhabdus bovienii SS-2004	BGC0002135/bovienimides[8]	XBJ1_2367-m3	DISNIGAIT	D-Cit	Trp	0.183
Streptomyces fungicidicus	BGC0000341/enduracidin A[9]	EndB-m7	DMEADGAVD	L-Cit	Cit	0.633

Table S7. Deduced proteins encoded in *Paenibacillus apiarius* MW-14's *pdn* BGC and their putative functions.

Protein	Amino Acids	Functional annotation	Sequence Similarity (protein, origin)	Similarity/Identity	NCBI Accession number
PdnA	539	Transcriptional activator	HTH-type transcriptional activator Btr, *Bacillus subtilis subsp. subtilis* strain 168	37.9/19.8	P40408.1[1]
PdnB	571	ABC transporter ATP-binding protein	YwjA, *Bacillus subtilis* strain 168	55.3/37.7	Q2LVL0.1[1]
PdnC	351	Trp hydroxylase	PrnB, *Pseudomonas fluorescens*	38.2/21.5	P95481[1]
PdnD	13735	NRPS (C-A-PCP-C-A-PCP-C-A-PCP-C-A-PCP-C-A-PCP-C-A-PCP-C-A-PCP-E-C-A-PCP-C-A-PCP-E-C-A-PCP-C-A-PCP-C-A-PCP-TE)	MCE5168246.1. NRPS, *Paenibacillus profundus* YoMME	93.8/89.6	WP_2336 95545.1
PdnE	299	Chitosanase	Csn, *Niallia circulans* (*Bacillus circulans*)	80.1/70.1	P33673[1]

[1]: from UniProtKB/Swiss-Prot database.

Table S8. Primers used in this study.

Name	Sequence (5' → 3')	Description
Gap1_ for	TACATGATTCCGTCGTAC	PCR-based closure of gap 1
Gap1_rev	AGATGTGCTTGATTCGTC	PCR-based closure of gap 1
Gap2_for	TGACGAATCAAGCACATC	PCR-based closure of gap 2
Gap2_rev	CTGCTGTCTATCTGAAGC	PCR-based closure of gap 2
Gap3_for	CTAACACCTAACGGCAAG	PCR-based closure of gap 3
Gap3_for	TAGCCCTGTATCATTCTG	PCR-based closure of gap 3
Gap4_for	GAATGATACAGGGCTACG	PCR-based closure of gap 4
Gap4_rev	CCTGTTGTCTATCTGAGG	PCR-based closure of gap 4

Table S9. Deduced proteins encoded in the *Paenibacillus* sp. P3_m182_1's *pdn* BGC and their putative functions.

**Protein**	**Amino Acids**	**Functional annotation**	**Sequence Similarity (protein, origin)**	**Similarity/ Identity**	**NCBI Accession number**
PdnA	248	RNA polymerase, σ-24 subunit	WYU59297.1. RNA polymerase sigma factor, *Paenibacillus* sp. FSL H8-0537	98.8/97.6	WP_341282625.1
PdnB	415	Major metabolite export protein	WYU59298.1 MFS transporter, *Paenibacillus* sp. FSL H8-0537	98.3/98.1	WP_341282626.1
PdnF	322	Precursor import protein	WYU59299.1. DMT family transporter, *Paenibacillus* sp. FSL H8-0537	99.1/99.1	WP_341282627.1
PdnG	194	Unknown	WYU59300.1. Hypothetical protein MHB80_13645, *Paenibacillus* sp. FSL H8-0537	97.4/96.4	WP_341282628.1
PdnD1	7877	NRPS (C-A-PCP-C-A-PCP-C-A-PCP-C-A-PCP-C-A-PCP-C-A-PCP-C-A-PCP-E)	WYU59301.1. NRPS, *Paenibacillus* sp. FSL H8-0537	55.8/55.2	WP_341282629.1
PdnD2	5956	NRPS (C-A-PCP-C-A-PCP-E-C-A-PCP-C-A-PCP-C-A-PCP-TE)	WYU59301.1. NRPS, *Paenibacillus* sp. FSL H8-0537	42.2/41.7	WP_341282629.1
PdnH	299	Unknown	YunF, *Bacillus subtilis* strain 168	64.7/53.7	O32135[1]
PdnI	148	Transcriptional regulator	WYU59303.1. MarR family transcriptional regulator, *Paenibacillus* sp. FSL H8-0537	98.7/97.3	WP_341282631.1

[1]: from UniProtKB/Swiss-Prot database.

Table S10. Genome mining for homologous *pdn* BGCs.

**Strain**	**Genbank accession no.**	**No. of predicted amino acids**
Paenibacillus alvei NRRL B-23219	JAMDMI010000001.1	12
Paenibacillus alvei NRRL B-23222	JAMDMG010000001.1	12
Paenibacillus alvei B-LR1	LS992241.1	12
Paenibacillus alvei DSM 29	AMBZ01000003.1	8
Paenibacillus apiarius MW-14	JAFFHZ010000001.1	12
Paenibacillus melissococcoides J10TS2	AP031286.1	10
Paenibacillus melissococcoides J1TS6	AP031298.1	10
Paenibacillus melissococcoides J22TS4	AP031304.1	10
Paenibacillus melissococcoides J46TS7	AP031310.1	10
Paenibacillus melissococcoides J6TS7	AP031316.1	10
Paenibacillus mucilaginosus K02	CP003422.2	12
Paenibacillus mucilaginosus 3016	CP003235.1	9
Paenibacillus mucilaginosus KN-18	CP035456.1	12
Paenibacillus mucilaginosus KNP414	CP002869.1	12
Paenibacillus mucilaginosus PM12	CP073316.1	12
Paenibacillus profundus YoMME	JAJNBZ010000001.1	12
Paenibacillus thiaminolyticus Mbale	JAALJM010000006.1	12
Paenibacillus thiaminolyticus Mbale2	CP094446.1	12
Paenibacillus thiaminolyticus SY20	CP106992.1	12
Paenibacillus thiaminolyticus PATH554	CP114031.1	12
Paenibacillus thiaminolyticus NRRL B-4156	CP041405.1	12
Paenibacillus suaedae chi10	JAVYAA010000002.1	12
Paenibacillus sp. FSL H8-0537	CP150290.1	12
Paenibacillus sp. KS-LC4	CP145905.1	12
Paenibacillus sp. MER 180	JAMAVT010000008.1	12
Paenibacillus alvei NRRL NRS-811	JAMDLX010000027.1	5 - incomplete
Paenibacillus alvei NRRL B-04300	JAMDNA010000047.1	5 - incomplete
Paenibacillus alvei NRRL B-04186	JAMDNK010000039.1	5 - incomplete
Paenibacillus alvei NRS-811	JARLKX010000045.1	5 - incomplete
Paenibacillus alvei NRRL B-04185	JAMDNL010000034.1	5 - incomplete
Paenibacillus alvei A6-6i-x	ATMS01000038.1	6 - incomplete
Paenibacillus alvei 32	JAOQAR020000004.1	8 - complete?
Paenibacillus alvei G25-117	LDHX01000012.1	4 - incomplete
Paenibacillus alvei TS-15	ATMT01000053.1	4 - incomplete
Paenibacillus apiarius NRS-1439	JARLKH010000052.1	7 - incomplete
Paenibacillus apiarius NRS-1578	JARLKE010000059.1	7 - incomplete
Paenibacillus apiarius NRRL B-04188	JAMDNI010000062.1	3 - incomplete
Paenibacillus apiarius NRRL B-04299	JAMDNB010000043.1	7 - incomplete
Paenibacillus apiarius NRRL B-23460	NZ_NDGJ01000005.1	4 - incomplete
	NZ_NDGJ01000026.1	4 - incomplete
Paenibacillus apiarius NRRL B-24729	JAMDMB010000003.1	7 - incomplete
	JAMDMB010000086.1	4 - incomplete
Paenibacillus apiarius NRRL B-03678	JAMDNS010000038.1	3 - incomplete
Paenibacillus apiarius NRRL B-04187	JAMDNJ010000008.1	7 - incomplete

Paenibacillus apiarius NRRL NRS-1439	JAMDLV010000014.1	3 - incomplete
Paenibacillus apiarius NRRL NRS-1438	JAMDLW010000050.1	7 - incomplete
Paenibacillus dendritisformis J6TS7	NZ_BORY01000111.1	5 - incomplete
Paenibacillus melissococcoides J11TS3	AP031292.1	7 - incomplete
Paenibacillus mucilaginosus G78	JAKQYK010000023.1	7 - incomplete
Paenibacillus sp. MER TA 81-3	JAMAWB010000001.1	7 - incomplete
Paenibacillus sp. S-12	NZ_JASIUF010000015.1	7- incomplete
Paenibacillaceae sp. P-4	JBEFZI010000016.1	7 - incomplete
Paenibacillus sp. OSY-SE	NZ_ALKF01000145.1	4 - incomplete
Paenibacillus sp. NAIST15-1	NZ_BBYF01000013.1	7 - incomplete
	NZ_BBYF01000017.1	3 - incomplete
Paenibacillus sp. Leaf72	LMLV01000023.1	incomplete
Paenibacillus thiaminolyticus BO5	QYZD01000072.1	3 - incomplete
Paenibacillus thiaminolyticus Mbale3	JALHBQ010000001.1	5 - incomplete
Paenibacillus thiaminolyticus NBRC 15656	NZ_BIMI01000136.1	3 - incomplete
Paenibacillus thiaminolyticus NRRL B-14609	NZ_JAMDMQ010000024.1	4 - incomplete
Paenibacillus thiaminolyticus NCTC11027	NZ_UGRZ01000002.1	4 - incomplete
	NZ_UGRZ01000003.1	7 - incomplete
Paenibacillus thiaminolyticus NRRL B-04156	JAMDNM010000058.1	6 - incomplete
	JAMDNM010000012.1	4 - incomplete
Paenibacillus thiaminolyticus NRRL B-24730	JAMDMA010000079.1	6 - incomplete
	JAMDMA010000077.1	4 - incomplete
Paenibacillus thiaminolyticus NRRL B-14625	JAMDML010000022.1	6 - incomplete
	JAMDML010000012.1	4 - incomplete
Paenibacillus thiaminolyticus NRRL B-14613	JAMDMM010000002.1	6 - incomplete
	JAMDMM010000017.1	4 - incomplete
Paenibacillus thiaminolyticus NRRL B-14612	JAMDMN010000072.1	6 - incomplete
	JAMDMN010000034.1	4 - incomplete
Paenibacillus thiaminolyticus NRRL B-14611	JAMDMO010000022.1	6 - incomplete
	JAMDMO010000020.1	4 - incomplete
Paenibacillus thiaminolyticus NRRL B-14607	JAMDMS010000037.1	6 - incomplete
	JAMDMS010000020.1	4 - incomplete
Paenibacillus thiaminolyticus NRRL B-14610	JAMDMP010000007.1	6 - incomplete
	JAMDMP010000018.1	4 - incomplete
Paenibacillus thiaminolyticus NRRL B-14609	JAMDMQ010000026.1	6 - incomplete
	JAMDMQ010000024.1	4 - incomplete
Paenibacillus thiaminolyticus NRRL B-14608	JAMDMR010000012.1	6 - incomplete
	JAMDMR010000009.1	4 - incomplete
Paenibacillus thiaminolyticus NRRL B-14605	JAMDMT010000060.1	6 - incomplete
	JAMDMT010000041.1	4 - incomplete
Paenibacillus thiaminolyticus NRRL B-14604	JAMDMU010000113.1	6 - incomplete
	JAMDMU010000060.1	4 - incomplete
Paenibacillus thiaminolyticus NRS-1591	JARLKD010000029.1	6 - incomplete
	JARLKD010000030.1	4 - incomplete
Paenibacillus thiaminolyticus NRS-1444	JARLKG010000014.1	6 - incomplete
	JARLKG010000015.1	4 - incomplete

Supplementary Figures

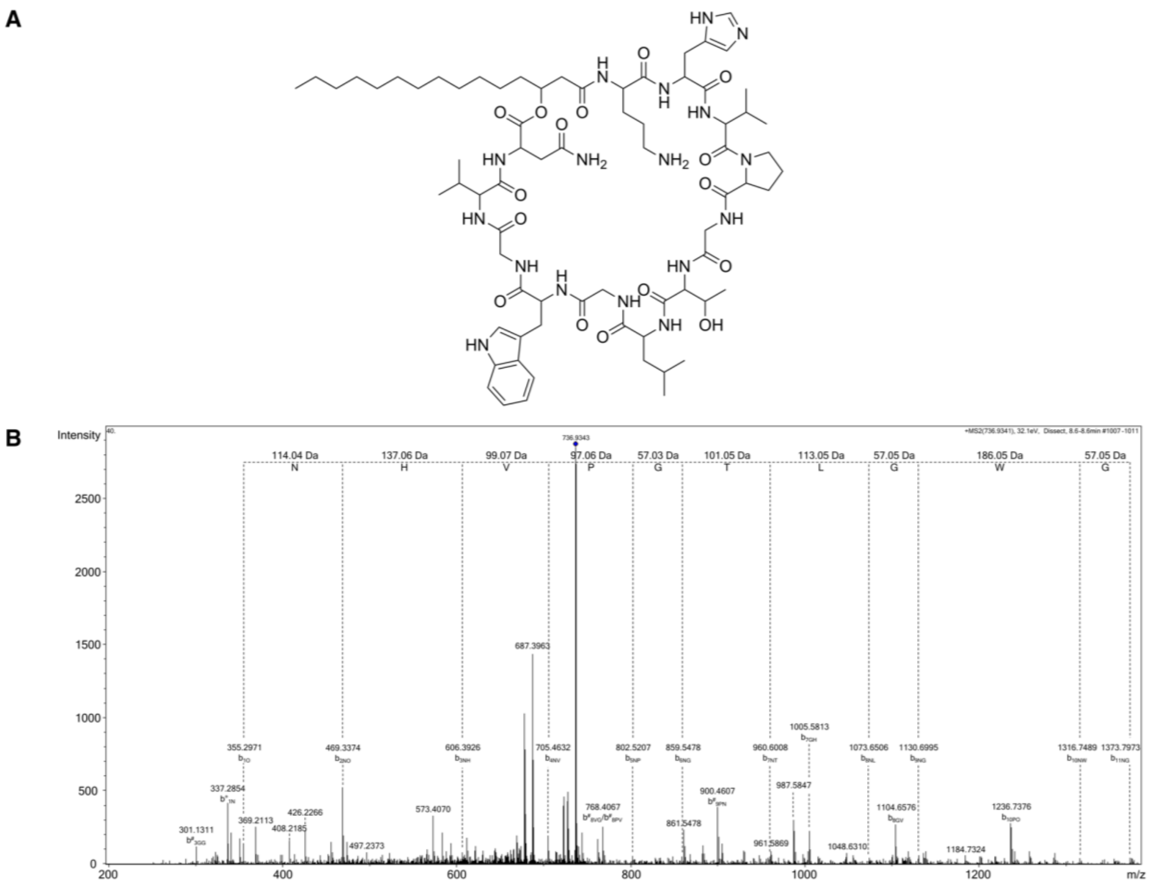

Figure S1. (A) Proposed chemical structure of **7** using the one-letter amino acid code. O = ornithine, H = histidine, V = valine, P' = 4-hydroxyproline, G = glycine, C' = citrulline, W' = 5-hydroxytryptophan, W = tryptophan, D = aspartic acid. FA = fatty acid (3-hydroxy-pentadecanoyl moiety). (B) Fragmentation pattern of **7** (*m*/z 736.93). Fragmentation annotations follow the nomenclature proposed by Ngoka.[10] b° = b-ion with loss of water, $b^{\#}$ = b-ion with loss of the acyl chain.

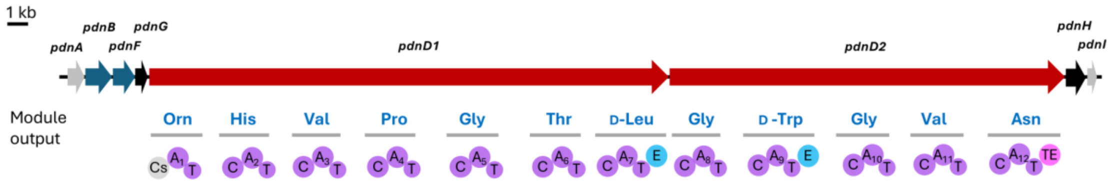

Figure S2. Organization of the *pdn* BGC from *Paenibacillllus* sp. P3_m182_1 (gray = regulator proteins, blue = transporter protein, black = hypothetical protein, red = NRPS).

A

	Paenibacillus apiarius MW-14			*Paenibacillus sp.* P3_m182_1		
Time point (days)	Monoculture	Coculture with *B. subtilis*	Coculture with *E. rubrum*	Monoculture	Coculture with *B. subtilis*	Coculture with *E. rubrum*
**Pellet extracts (AUCs in µV*sec)**						
0	269759	255433	351567	20940	22872	20579
4	547216	348022	502242	71514	57408	ND
7	525825	462034	463098	28174	54483	ND
10	303357	307862	241100	32256	11848	ND
14	127231	110516	197893	ND	40095	ND
**Supernatant extracts (AUCs in µV*sec)**						
0	ND	ND	ND	ND	ND	ND
4	9151	10018	ND	ND	ND	ND
7	ND	ND	ND	ND	ND	ND
10	ND	ND	ND	ND	ND	ND
14	5137	ND	ND	ND	ND	ND

ND: non-detectable

B

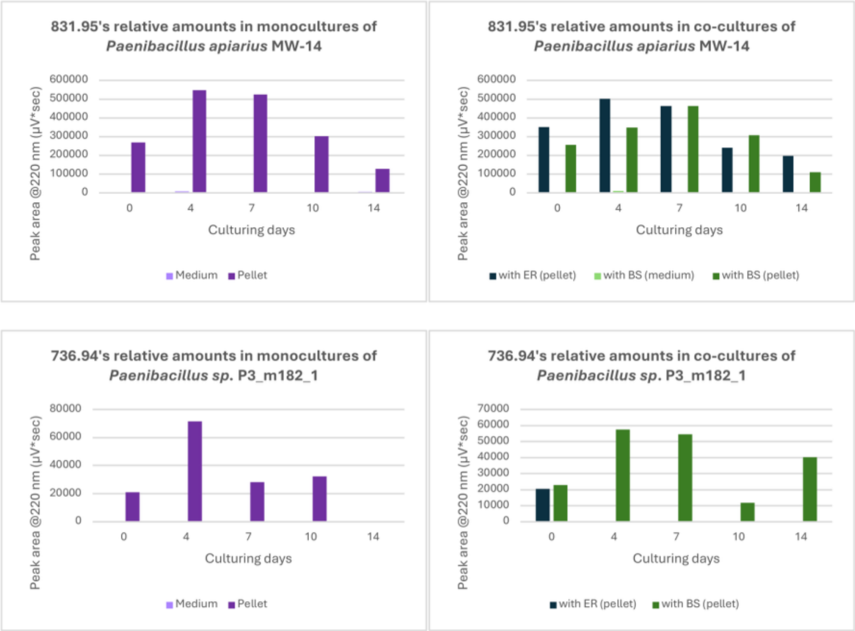

Figure S3. Comparative analysis of peptide production in *Paenibacilllus* sp. P3_m182_1 and *Paenibacillus apiarius* MW-14. (A) Summary table of AUC values corresponding to the bar graphs. (B) Bar graphs depicting AUC values (µV*sec) at 220 nm for primary peptide signals (m/z 736.94 for strain *Paenibacillus* sp. P3_m182_1 and m/z 831.95 for *P. apiarius* MW1-14) across various culture conditions and time points. Cultures were grown as monocultures and co-cultures with *B. subtilis* JH642 + *sfp* (BS) or with *E. rubrum* DSMZ 62631 (ER). Co-cultures were initiated by introducing *B. subtilis* or *E. rubrum* after 4 days of *Paenibacillus* spp. growth, designated as “day 0”. Samples were extracted with *n*-butanol at 1 h, and on days 4, 7, 10 and 14 post-inoculation. Both supernatant and pellet extracts were analyzed.

A

B

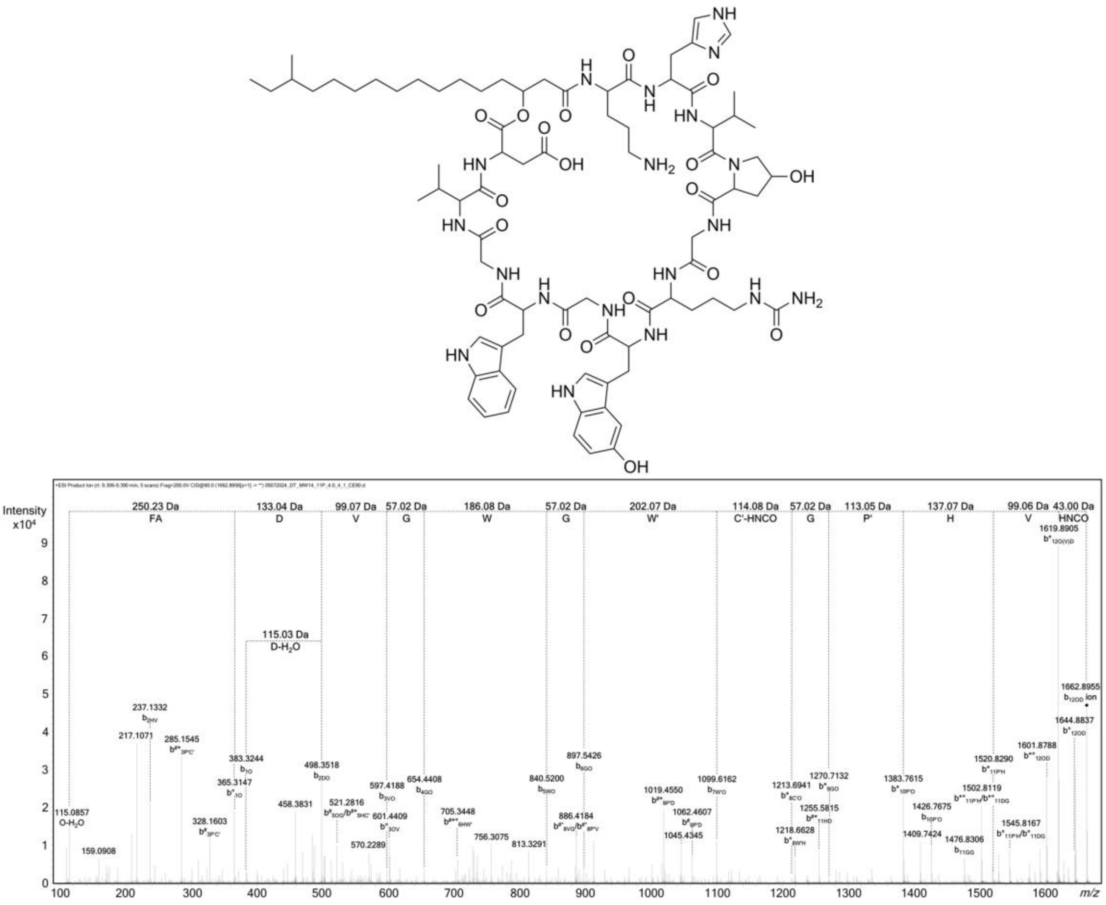

Figure S4. (A) Chemical structure of paenidepsin A (**1**) using the one-letter amino acid code. O = ornithine, H = histidine, V = valine, P' = 4-hydroxyproline, G = glycine, C' = citrulline, W' = 5-hydroxytryptophan, W = tryptophan, D = aspartic acid. FA = fatty acid (3-hydroxy-14-methylhexadecanoyl moiety). (B) Fragmentation pattern of **1** (*m*/z 1662.89). Fragmentation annotations follow the nomenclature proposed by Ngoka.[10] b° = b-ion with loss of water, b* = b-ion with loss of isocyanic acid (HNCO). $b^{\#}$ = b-ion with loss of the acyl chain.

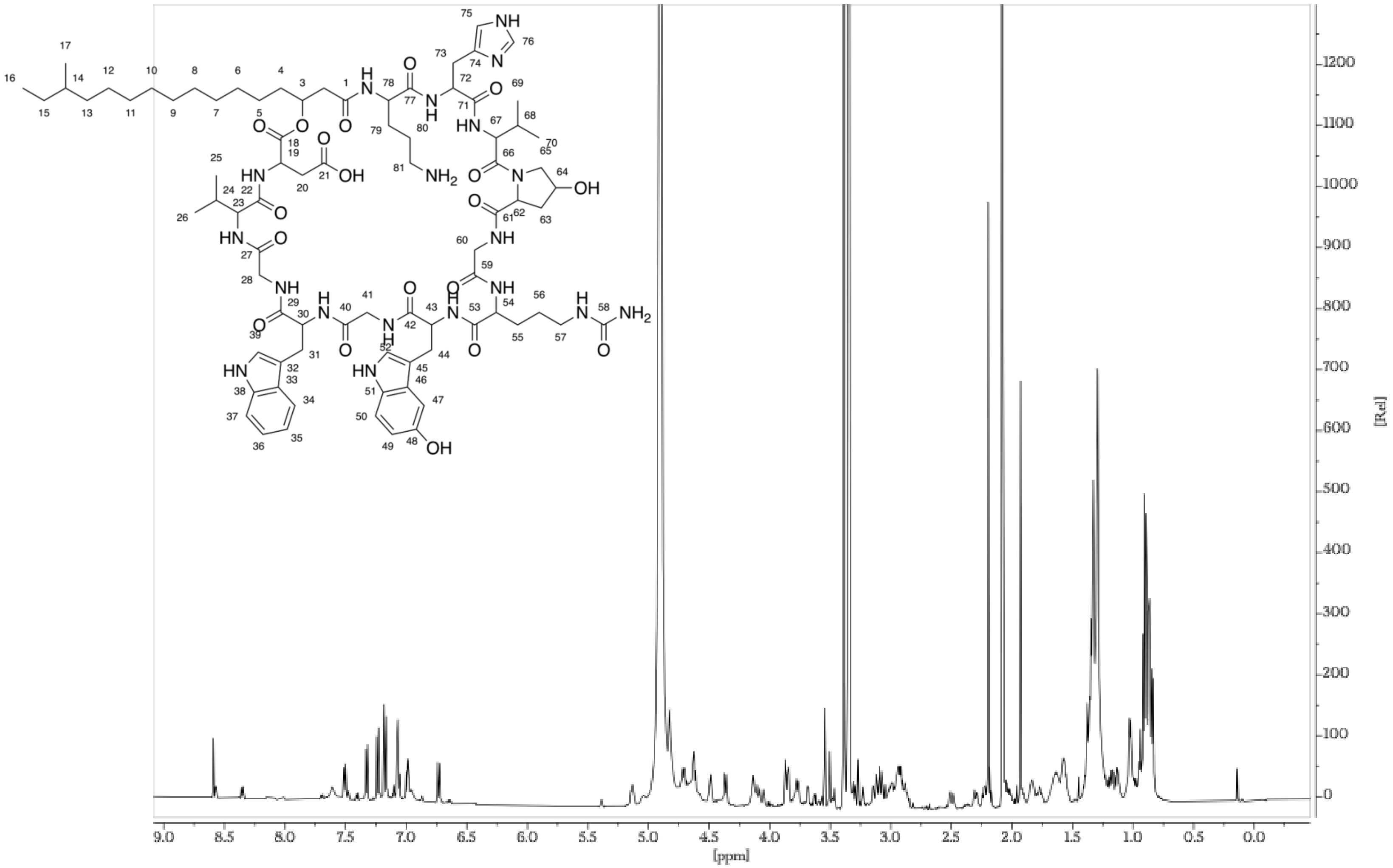

Figure S5. ^{1}H NMR spectrum of paenidepsin A (**1**) in methyl alcohol-d_4 (600 MHz).

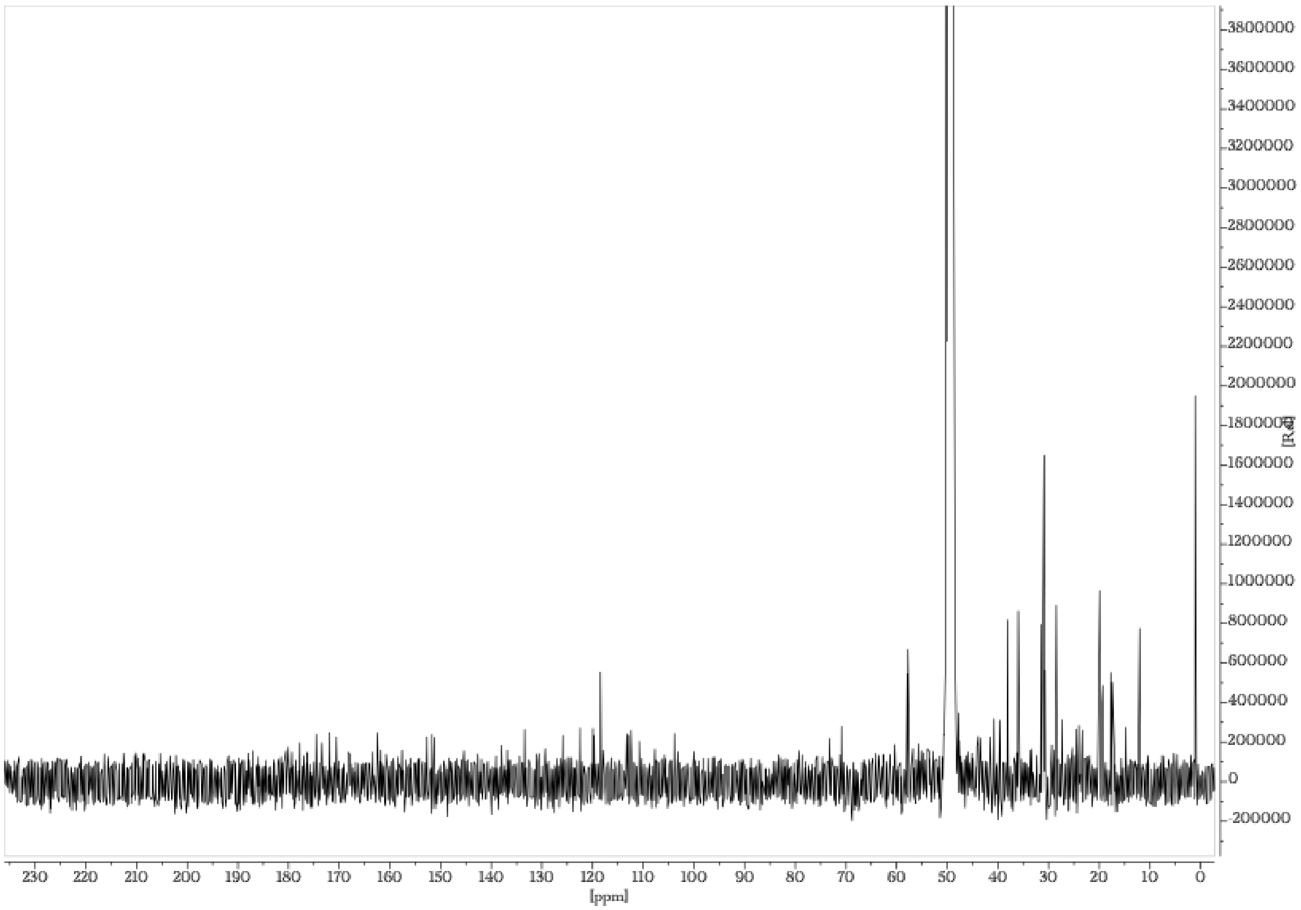

Figure S6. ^{13}C NMR spectrum of paenidepsin A (**1**) in methyl alcohol-d_4 (150 MHz).

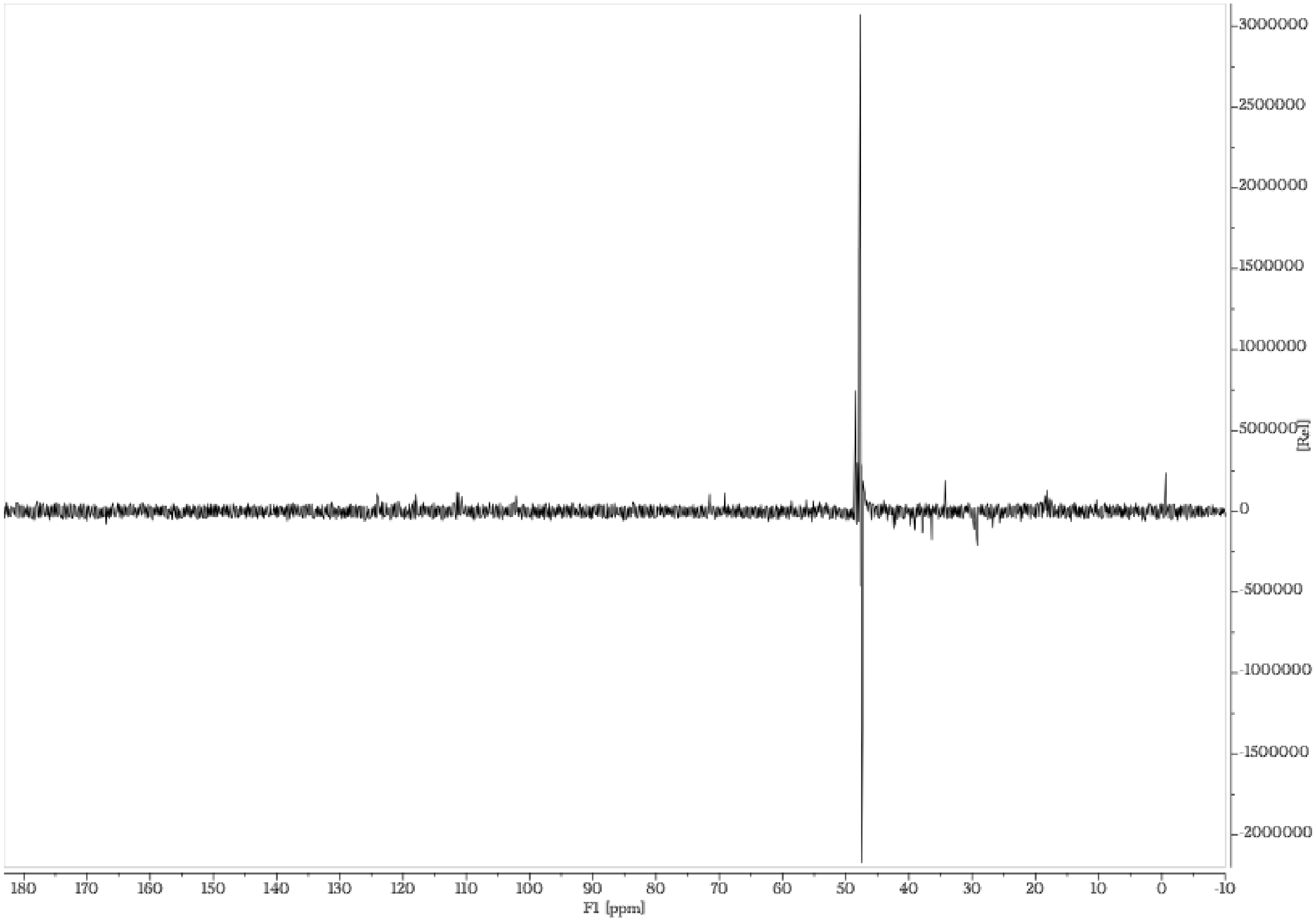

Figure S7. DEPT-135 NMR spectrum of paenidepsin A (**1**) in methyl alcohol-d_4 (150 MHz).

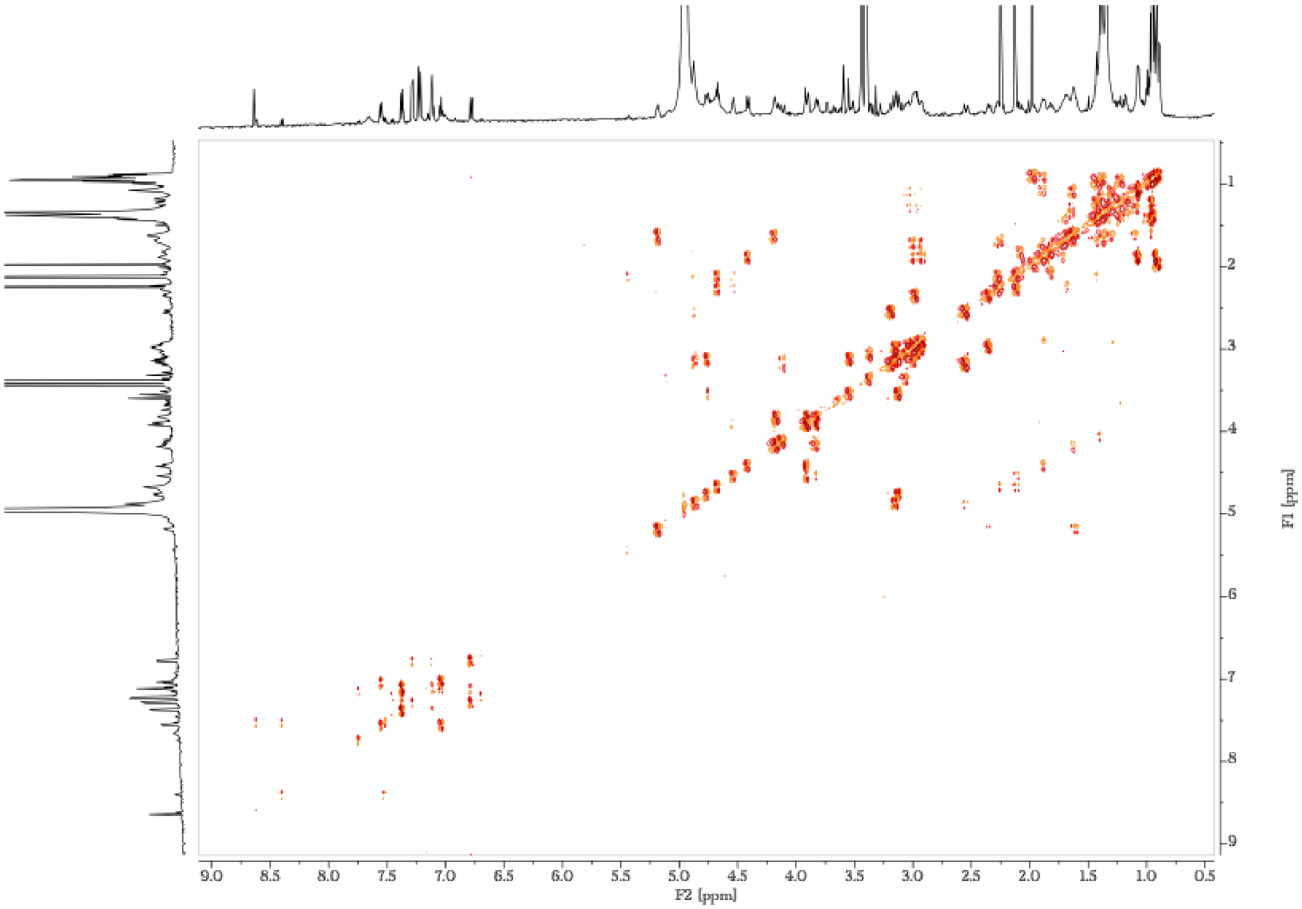

Figure S8. ^{1}H-^{1}H COSY NMR spectrum of paenidepsin A (**1**) in methyl alcohol-d_4 (600 MHz).

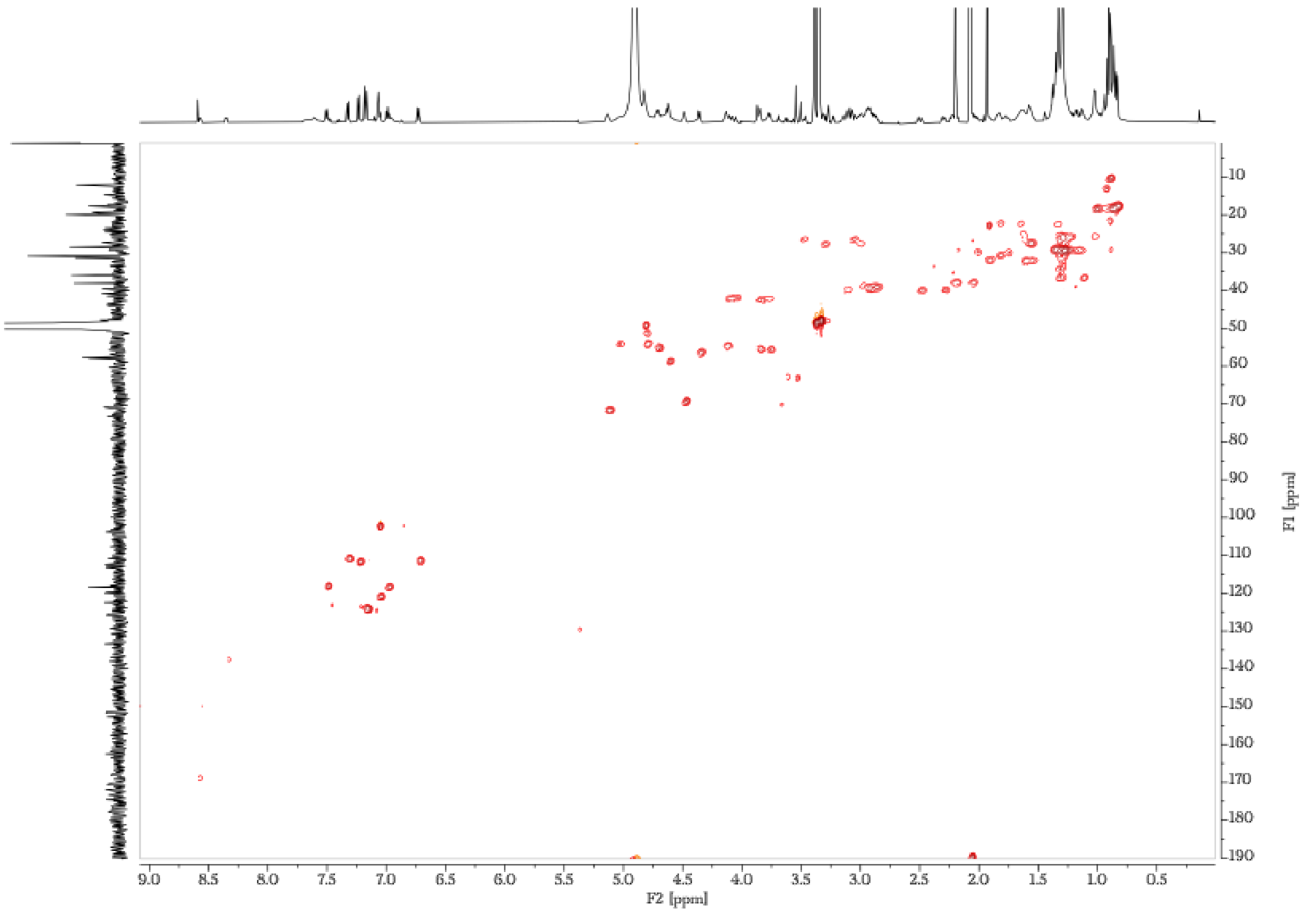

Figure S9. ^{1}H-^{13}C HSQC NMR spectrum of paenidepsin A (**1**) in methyl alcohol-d_4 (600 MHz).

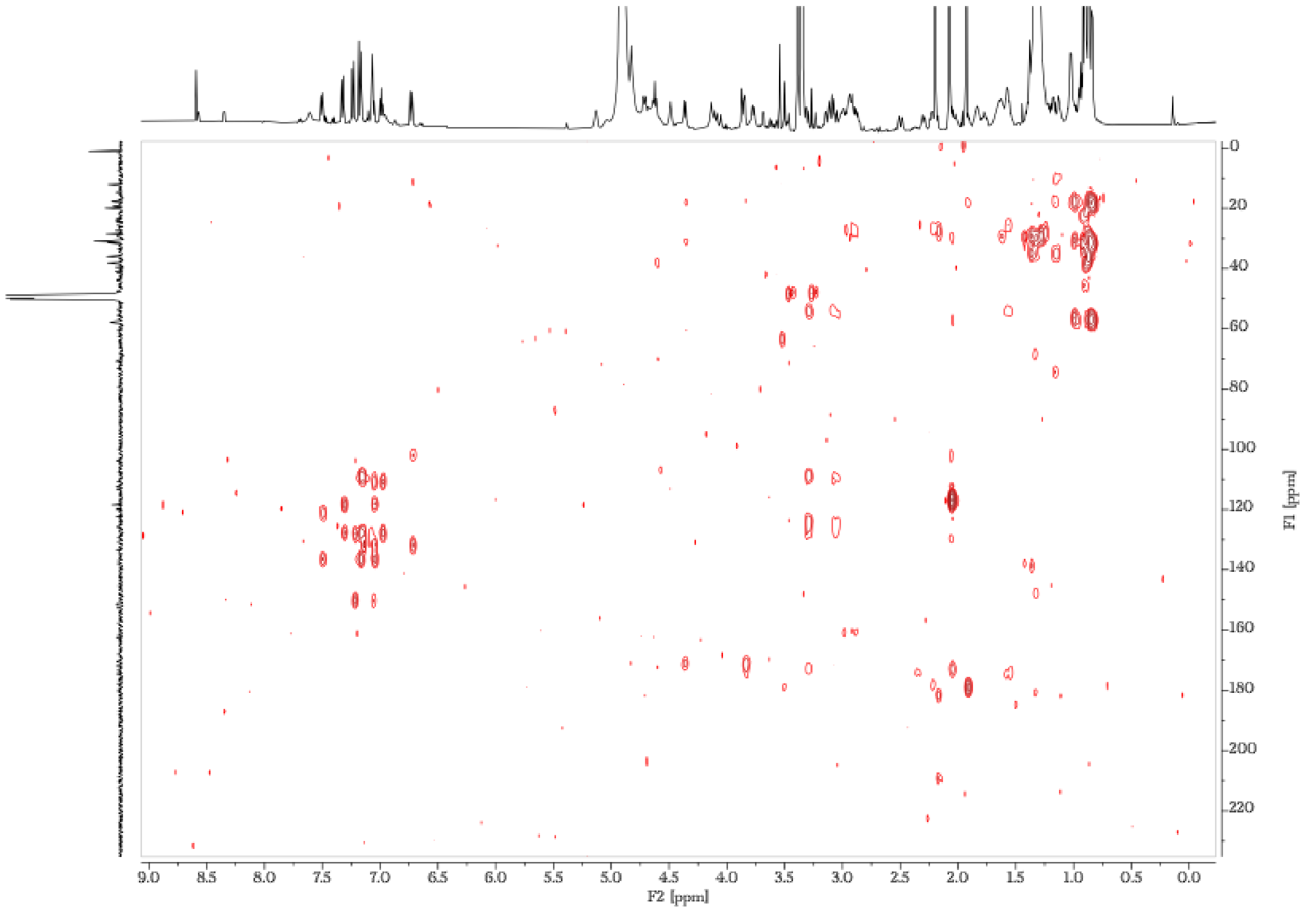

Figure S10. ^{1}H-^{13}C HMBC NMR spectrum of paenidepsin A (**1**) in methyl alcohol-d$_4$ (700 MHz).

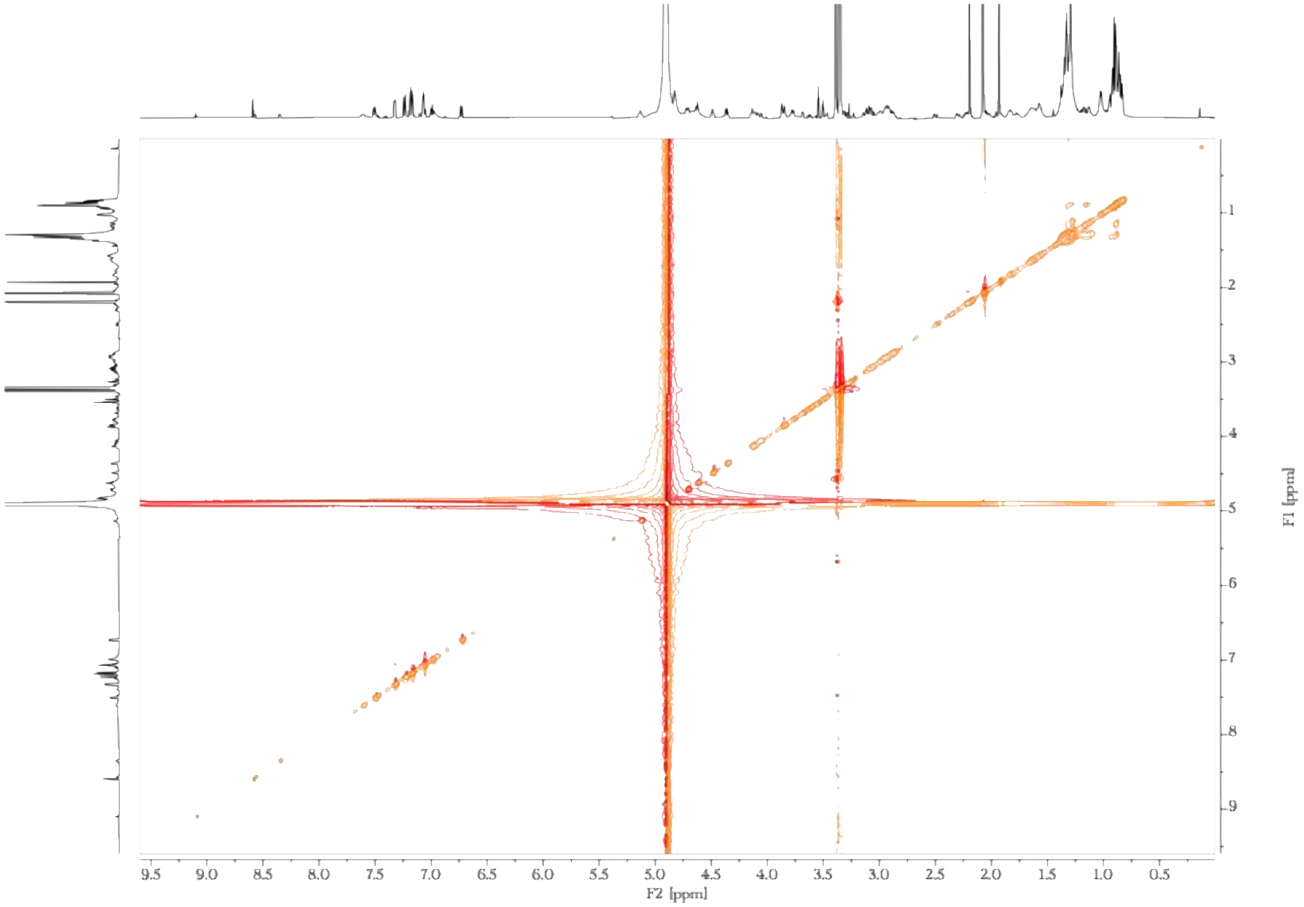

Figure S11. ^{1}H-^{1}H ROESY NMR spectrum of paenidepsin A (**1**) in methyl alcohol-d$_4$ (600 MHz).

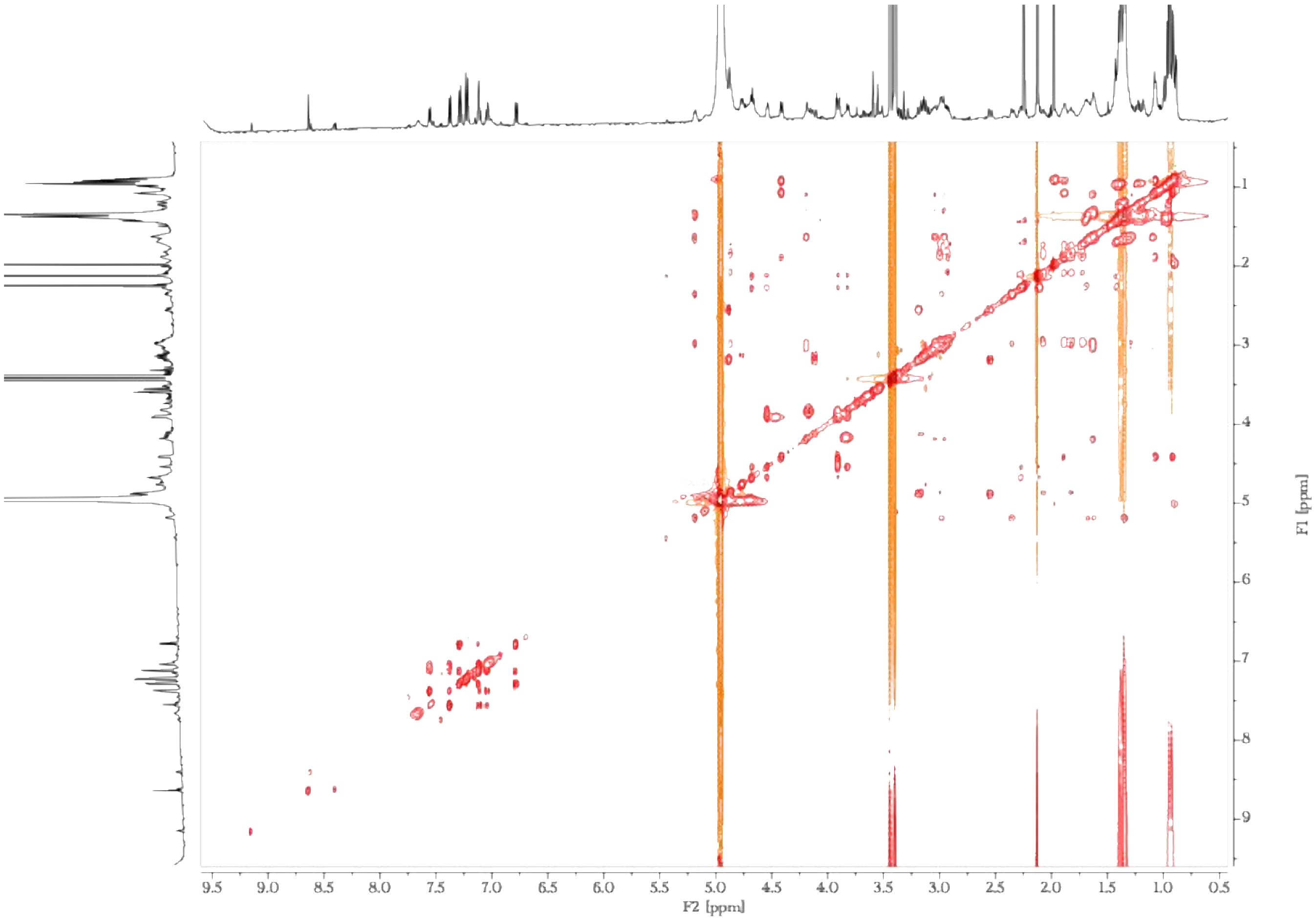

Figure S12. ^{1}H-^{1}H TOCSY NMR spectrum of paenidepsin A (**1**) in methyl alcohol-d$_4$ (600 MHz).

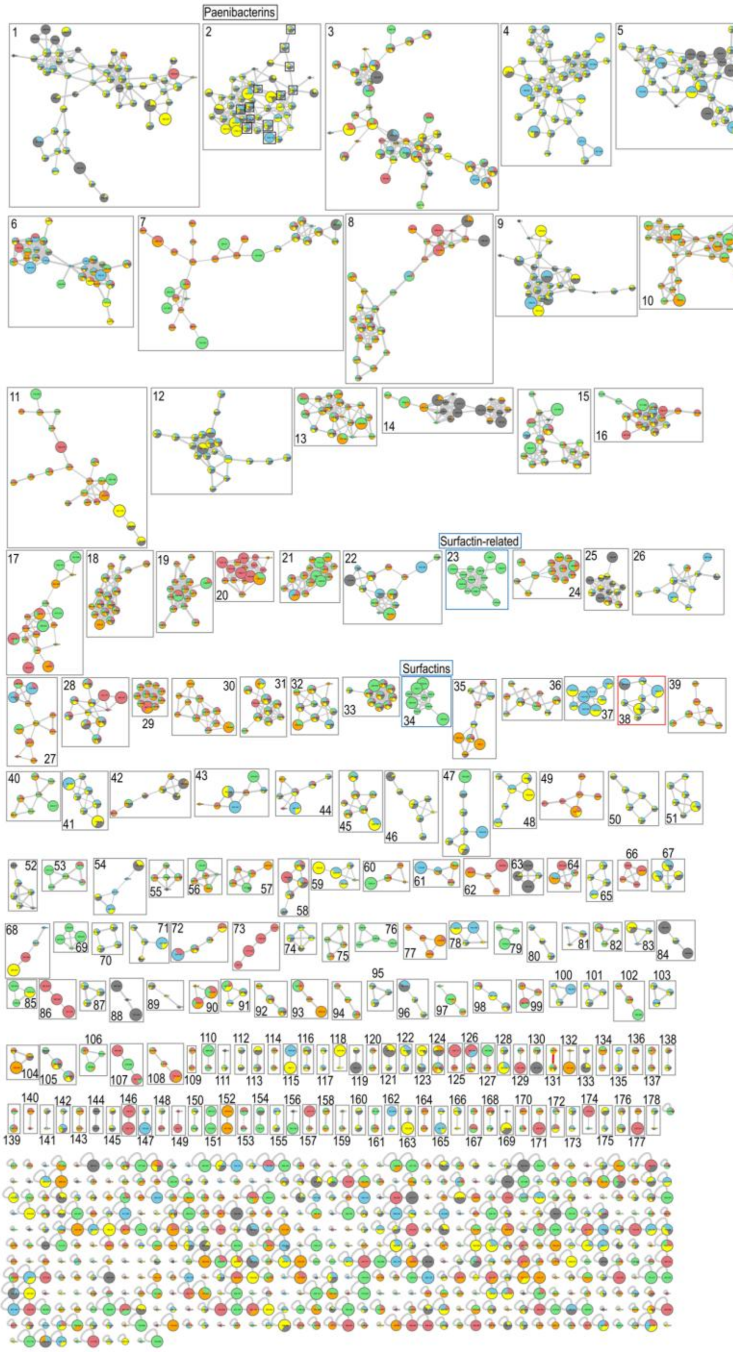

Figure S13. Molecular network of *Paenibacillus* spp. *n*-butanolic extracts from *Paenibacillus* strains. *Paenibacillus apiarius* MW-14 as a monoculture (blue), as a coculture with *Bacillus subtilis* JH642 + *sfp* (yellow), or with *Eurotium rubrum* DSMZ 62631 (gray), from *Paenibacillus* sp. P3_m182_1 as a monoculture (red), as a coculture with *B. subtilis* JH642 + *sfp* (green) or with *E. rubrum* DSMZ 62631 (orange). The cluster containing paenidepsin A congeners (38) is highlighted by a red box. All 178 clusters containing at least two nodes are numbered.

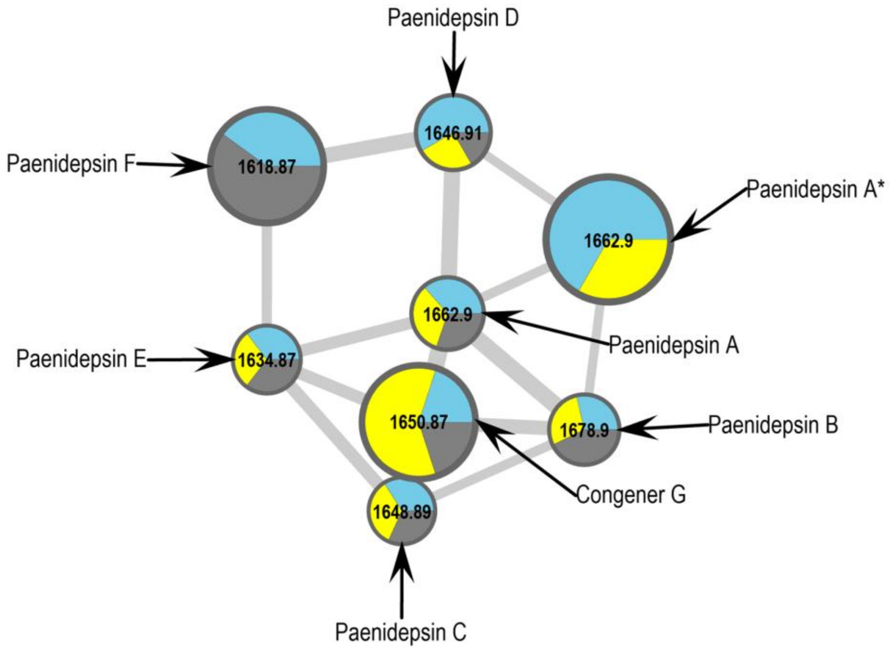

Figure S14. Molecular cluster of paenidepsin A (determined by MS^2) from the molecular network (Figure S13). The data pool includes extracts of *Paenibacillus apiarius* MW-14 cultivated as a monoculture in TSB medium and extracts of the same strain cocultured with whether *Bacillus subtilis* JH642 + *sfp* or *Eurotium rubrum* DSMZ 62631. Nodes display their parent mass. Their size represents the number of spectra obtained. The width of the edges corresponds to the similarity of the fragmentation spectra of the connected nodes, while node color displays their origin (blue: produced by *P. apiarius* MW-14, yellow: produced by *P. apiarius* MW-14 cocultured with *B. subtilis* JH642 + *sfp*, gray: produced by *P. apiarius* MW-14 cocultured with *E. rubrum* DSMZ 62631). Identified paenidepsin A congeners (Figure 1) are indicated by arrows and named. *: Node represents an $[M + H]^+$ adduct, while the other nodes represent an $[M + 2H]^{2+}$ adduct.

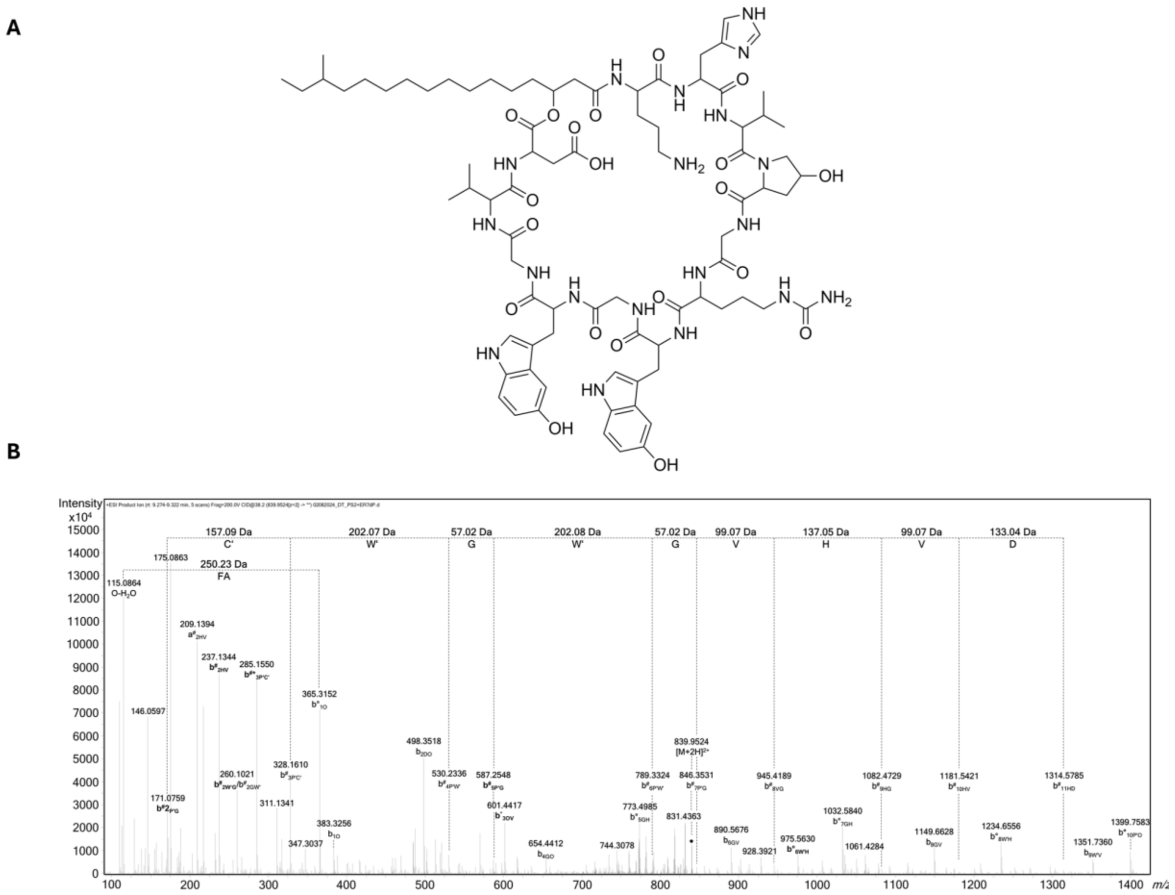

Figure S15. (A) Proposed chemical structure of paenidepsin B (**2**) using the one-letter amino acid code. O = ornithine, H = histidine, V = valine, P’ = 4-hydroxyproline, G = glycine, C’ = citrulline, W’ = 5-hydroxytryptophan, W = tryptophan, D = aspartic acid. FA = fatty acid (3-hydroxy-14-methylhexadecanoyl moiety). (B) Fragmentation pattern of **2** (*m*/z 839.9524). Fragmentation annotations follow the nomenclature proposed by Ngoka.[10] $a^{\#}$ = a-ion with loss of the acyl chain, b° = b-ion with loss of water, b* = b-ion with loss of isocyanic acid (HNCO). $b^{\#}$ = b-ion with loss of the acyl chain.

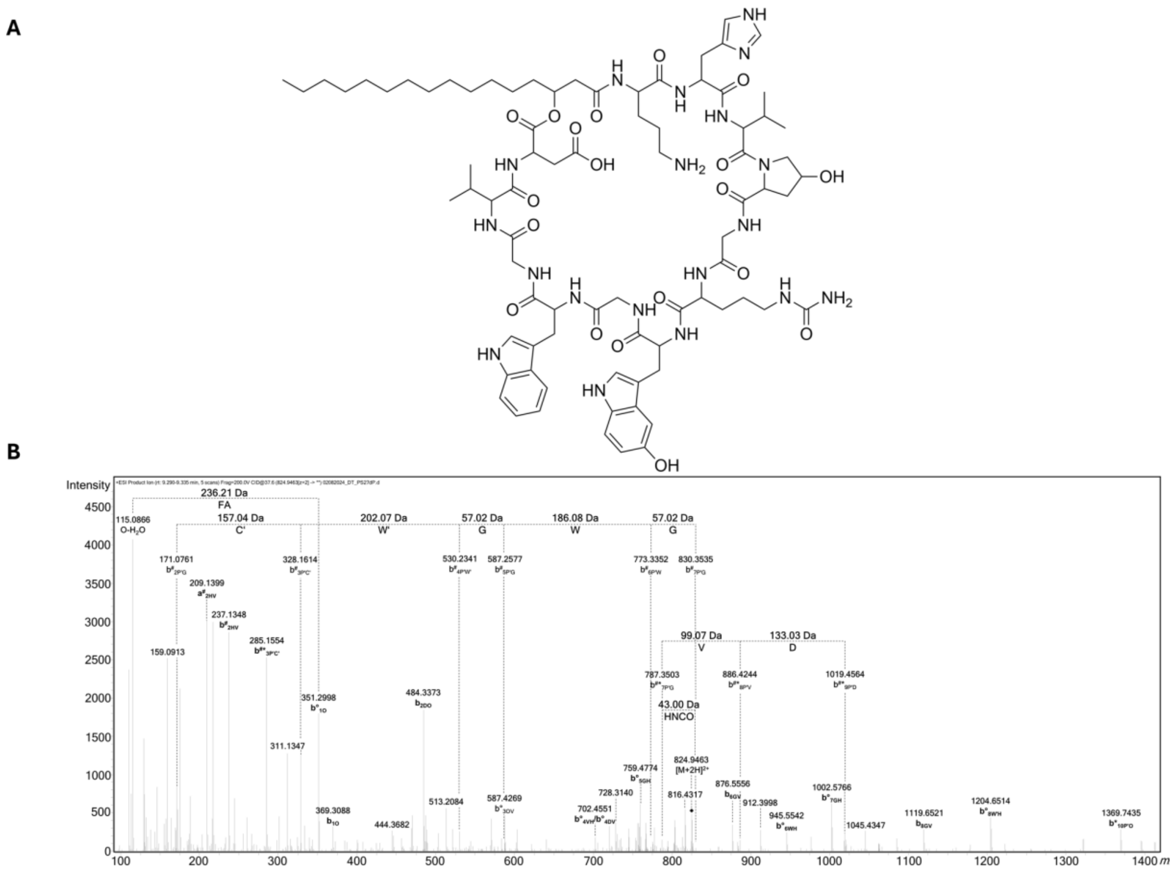

Figure S16. (A) Proposed chemical structure of paenidepsin C (**3**) using the one-letter amino acid code. O = ornithine, H = histidine, V = valine, P' = 4-hydroxyproline, G = glycine, C' = citrulline, W' = 5-hydroxytryptophan, W = tryptophan, D = aspartic acid. FA = fatty acid (3-hydroxy-hexadecanoyl moiety). (B) Fragmentation pattern of **3** (*m*/z 824.9463). Fragmentation annotations follow the nomenclature proposed by Ngoka.[10] $a^{\#}$= a-ion with loss of the acyl chain. b° = b-ion with loss of water, b* = b-ion with loss of isocyanic acid (HNCO). $b^{\#}$ = b-ion with loss of the acyl chain.

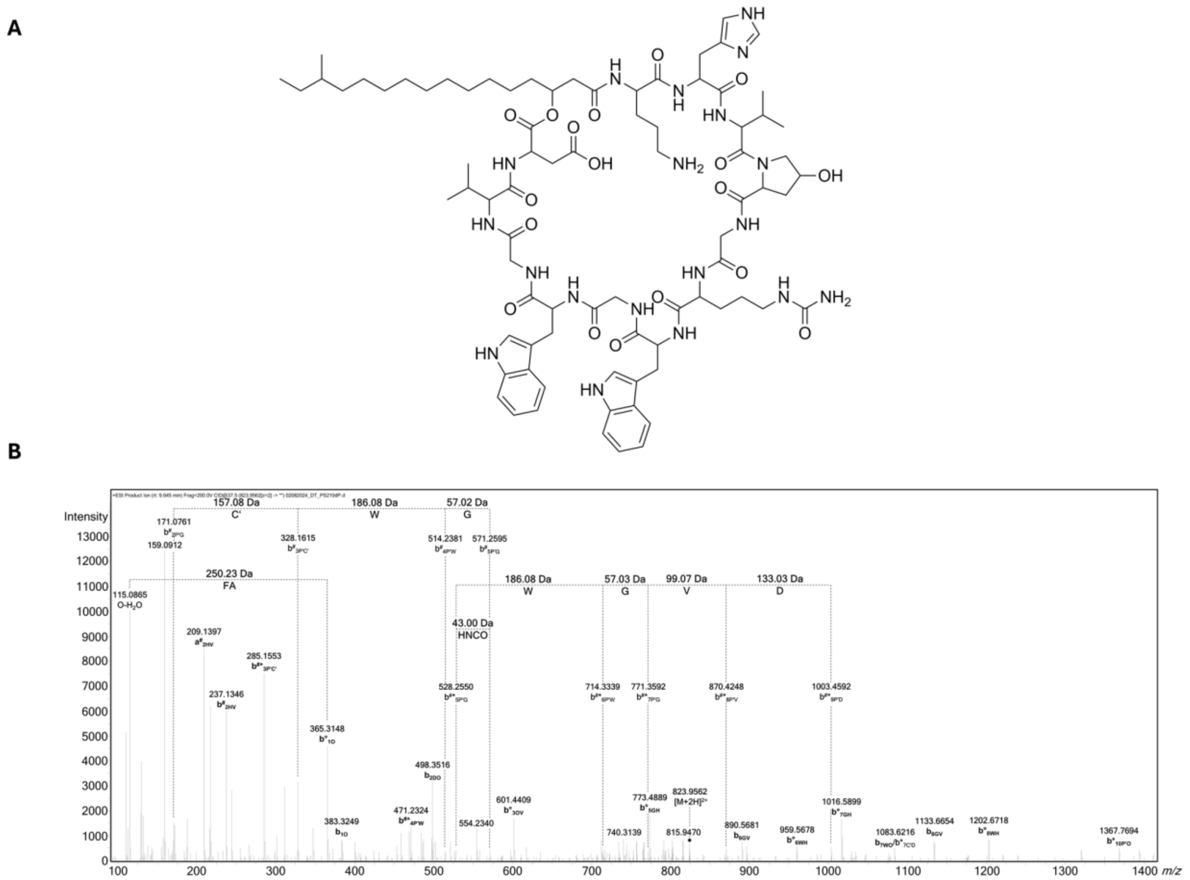

Figure S17. (A) Proposed chemical structure of paenidepsin D (**4**) using the one-letter amino acid code. O = ornithine, H = histidine, V = valine, P' = 4-hydroxyproline, G = glycine, C' = citrulline, W = tryptophan, D = aspartic acid. FA = fatty acid (3-hydroxy-14-methylhexadecanoyl moiety). (B) Fragmentation pattern of **4** (*m*/z 823.9562). Fragmentation annotations follow the nomenclature proposed by Ngoka.[10] $a^{\#}$= a-ion with loss of the acyl chain. b° = b-ion with loss of water, b* = b-ion with loss of isocyanic acid (HNCO). $b^{\#}$ = b-ion with loss of the acyl chain.

A

B

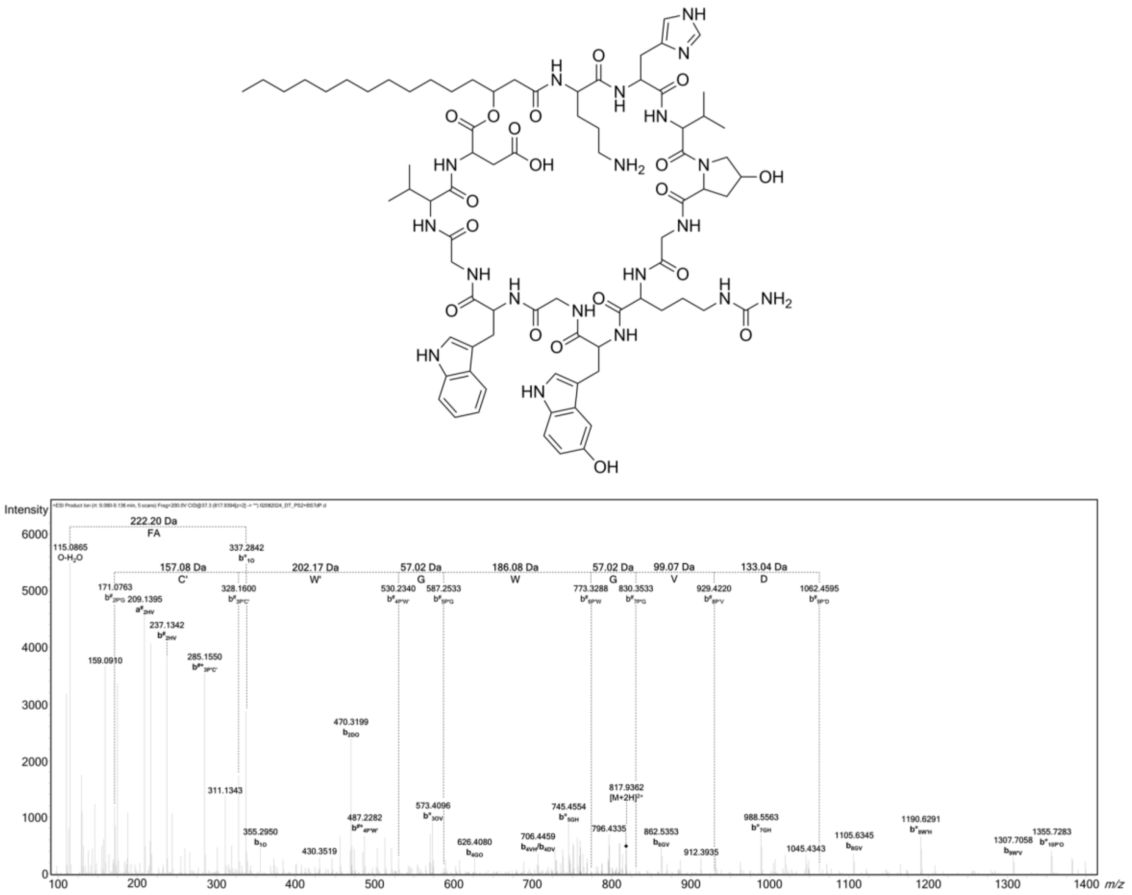

Figure S18. (A) Proposed chemical structure of paenidepsin E (**5**) using the one-letter amino acid code. O = ornithine, H = histidine, V = valine, P' = 4-hydroxyproline, G = glycine, C' = citrulline, W' = 5-hydroxytryptophan, W = tryptophan, D = aspartic acid. FA = fatty acid (3-hydroxy-pentadecanoyl moiety). (B) Fragmentation pattern of **5** (*m*/z 817.9394). Fragmentation annotations follow the nomenclature proposed by Ngoka.[10] $a^{\#}$= a-ion with loss of the acyl chain. b° = b-ion with loss of water, b* = b-ion with loss of isocyanic acid (HNCO). $b^{\#}$ = b-ion with loss of the acyl chain.

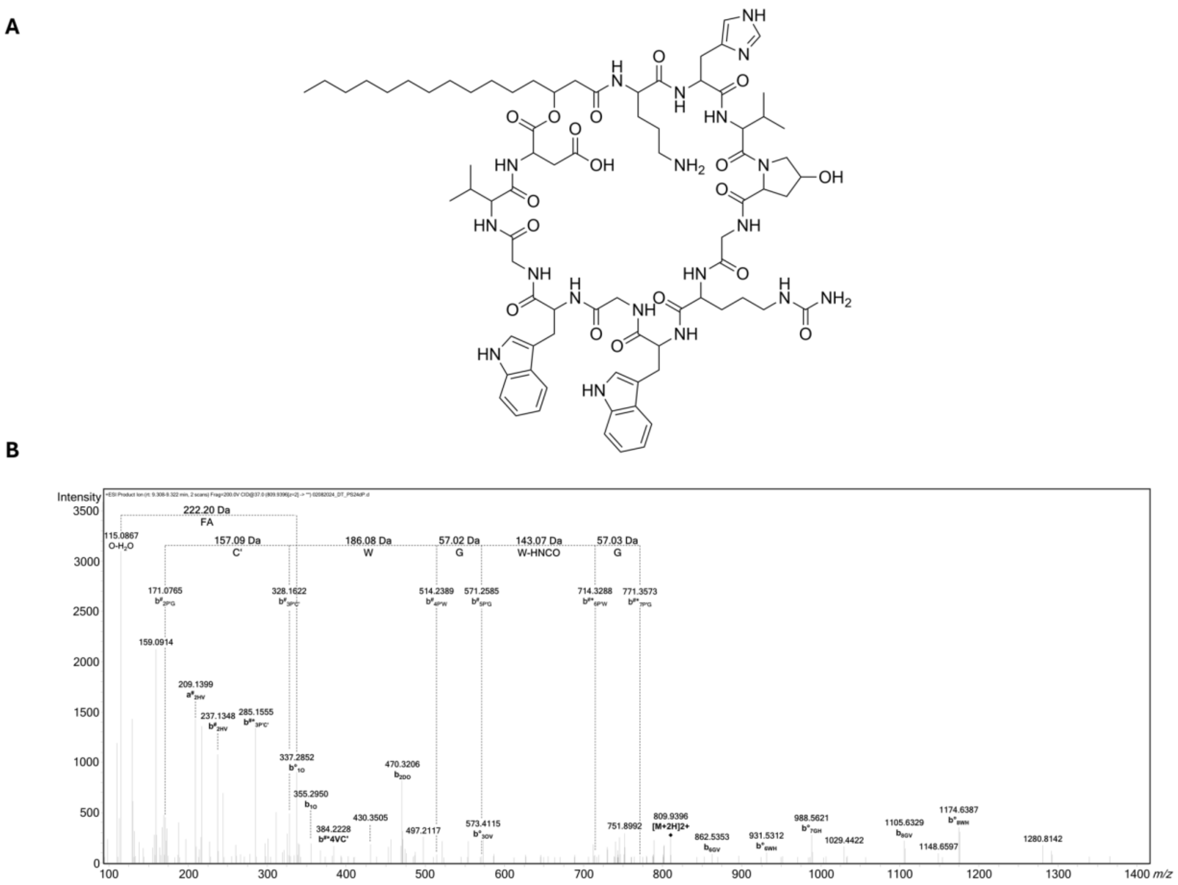

Figure S19. (A) Proposed chemical structure of paenidepsin F (**6**) using the one-letter amino acid code. O = ornithine, H = histidine, V = valine, P' = 4-hydroxyproline, G = glycine, C' = citrulline, W = tryptophan, D = aspartic acid. FA = fatty acid (3-hydroxy-pentadecanoyl moiety). (B) Fragmentation pattern of **6** (*m*/z 809.9396). Fragmentation annotations follow the nomenclature proposed by Ngoka.[10] $a^{\#}$= a-ion with loss of the acyl chain. b° = b-ion with loss of water, b* = b-ion with loss of isocyanic acid (HNCO). $b^{\#}$ = b-ion with loss of the acyl chain.

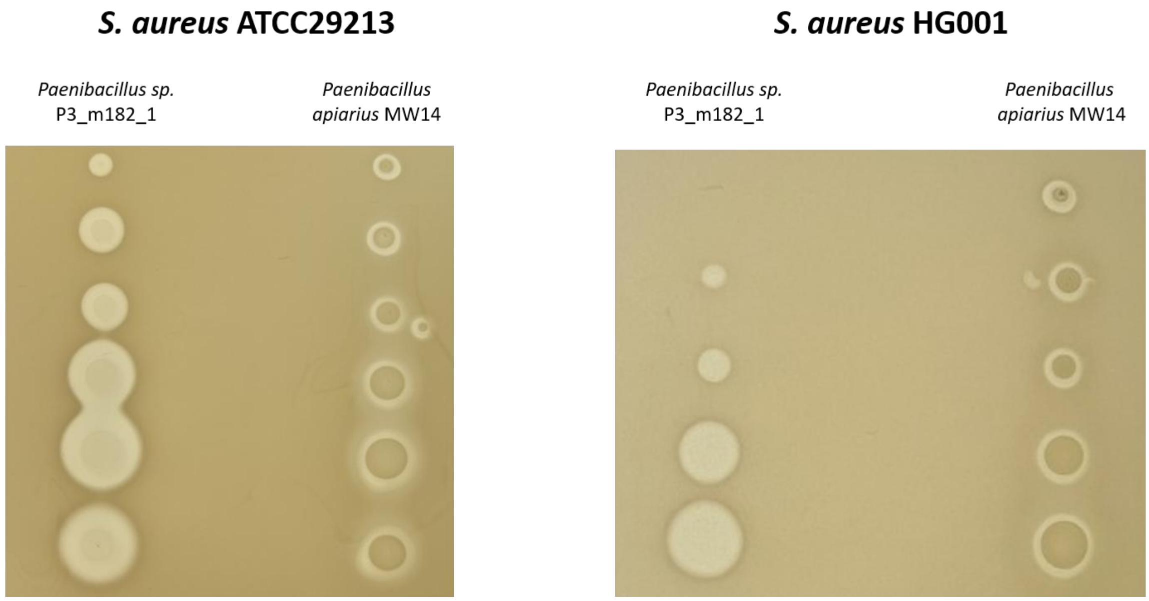

Figure S20. Results of antibacterial overlay assays of *Paenibacillus* sp. P3_m182_1 and *Paenibacillus apiarius* MW14 against *Staphylococcus aureus* ATCC 29213 (left) and *Staphylococcus aureus* HG 001 (right). The spots (up to down) correspond to increasing amounts of *Paenibacillus* cultures (from 0.5 µL up to 12 µL) applied to the Petri dishes.

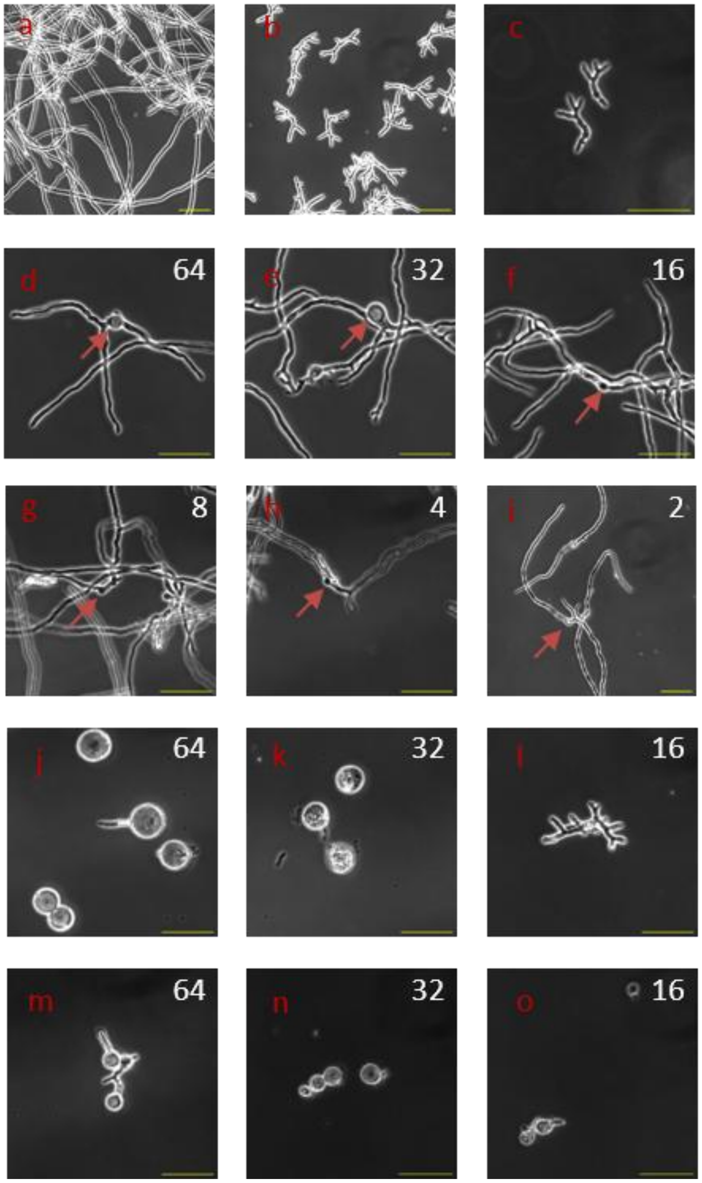

Figure S21. Microscopic images of morphological changes in *A. fumigatus* DSM819 hyphal structures after treatment with paenidepsin A alone or in combination with micafungin. Images of (**a**) control cells, and cells treated with (**b**) micafungin 0.015625 µg/mL, (**c**) cells treated with MeOH and micafungin 0.015625 µg/mL and (**d-i**) paenidepsin A 64 µg/mL – 2 µg/mL. While paenidepsin A alone induced irregular, bulbous swellings along the hyphal elements, treatment in combination with micafungin (0.015625 µg/mL, **j-l** or 0.15625 µg/mL **m-o**, paenidepsin A concentrations 64-16 µg/mL) resulted in spherical, blastospore-like structures and filamentous architecture was largely abolished after incubation for 17 h. Scale bar 50 µm. Paenidepsin A concentrations in µg/mL are given in white.

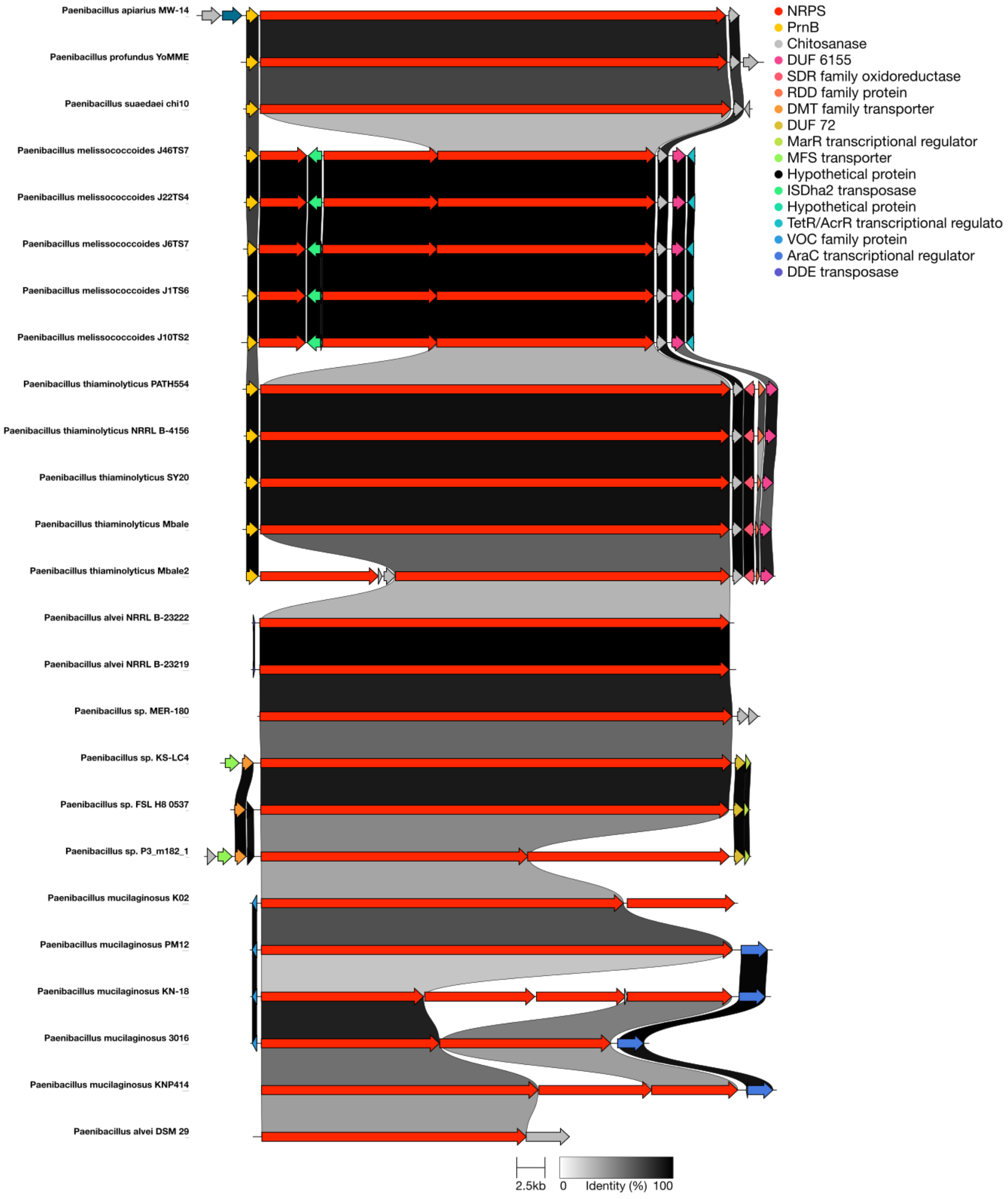

Figure S22. Comparison of genes and encoded proteins in the non-fragmented *pdn* BGCs.

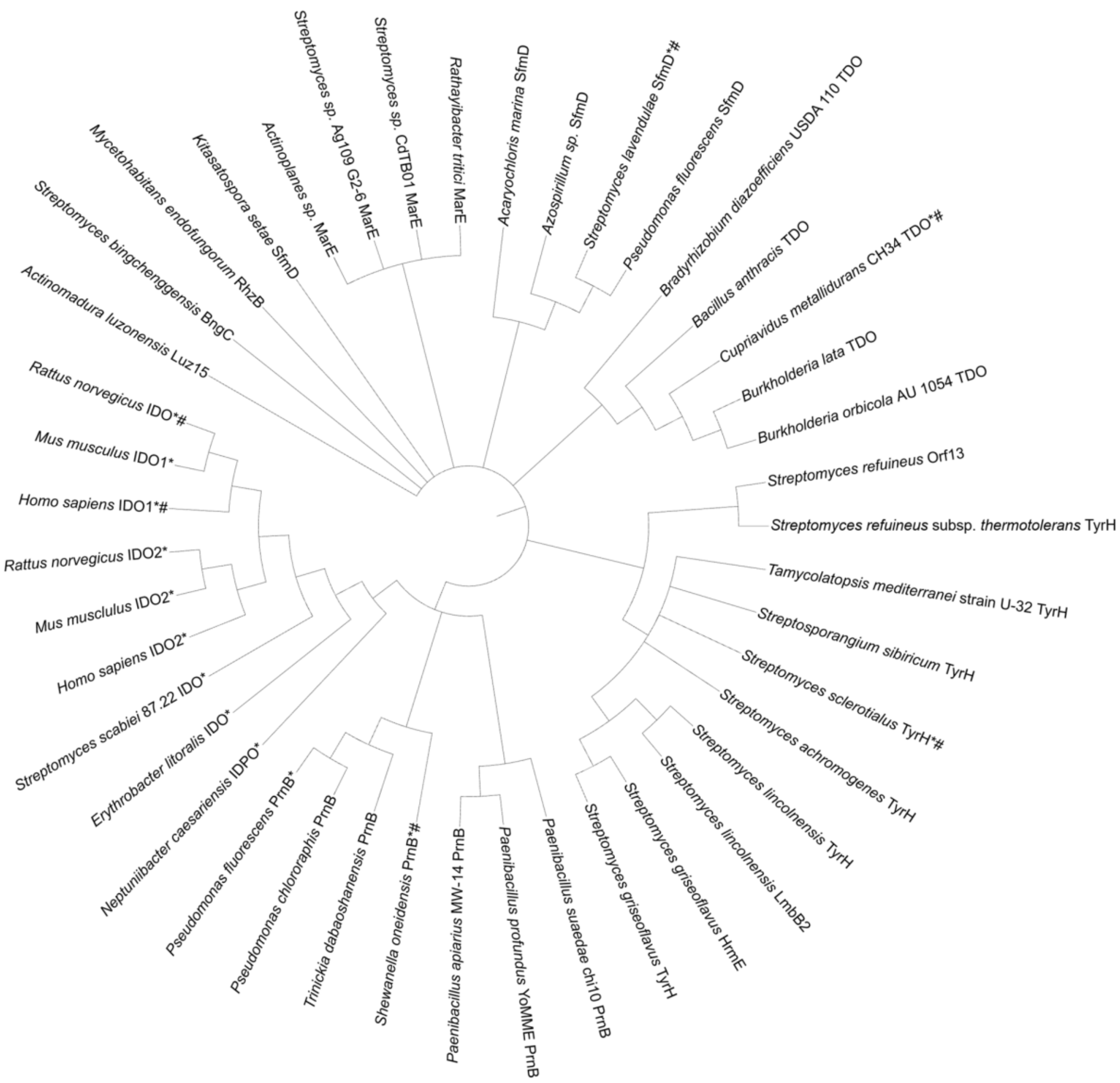

Figure S23. Phylogenetic tree of HDAOs and PdnC including Luz15. *: Proteins with reported bioactivity. #: Proteins with experimentally determined crystal structures. Branch lengths are proportional to evolutionary distance.[11]

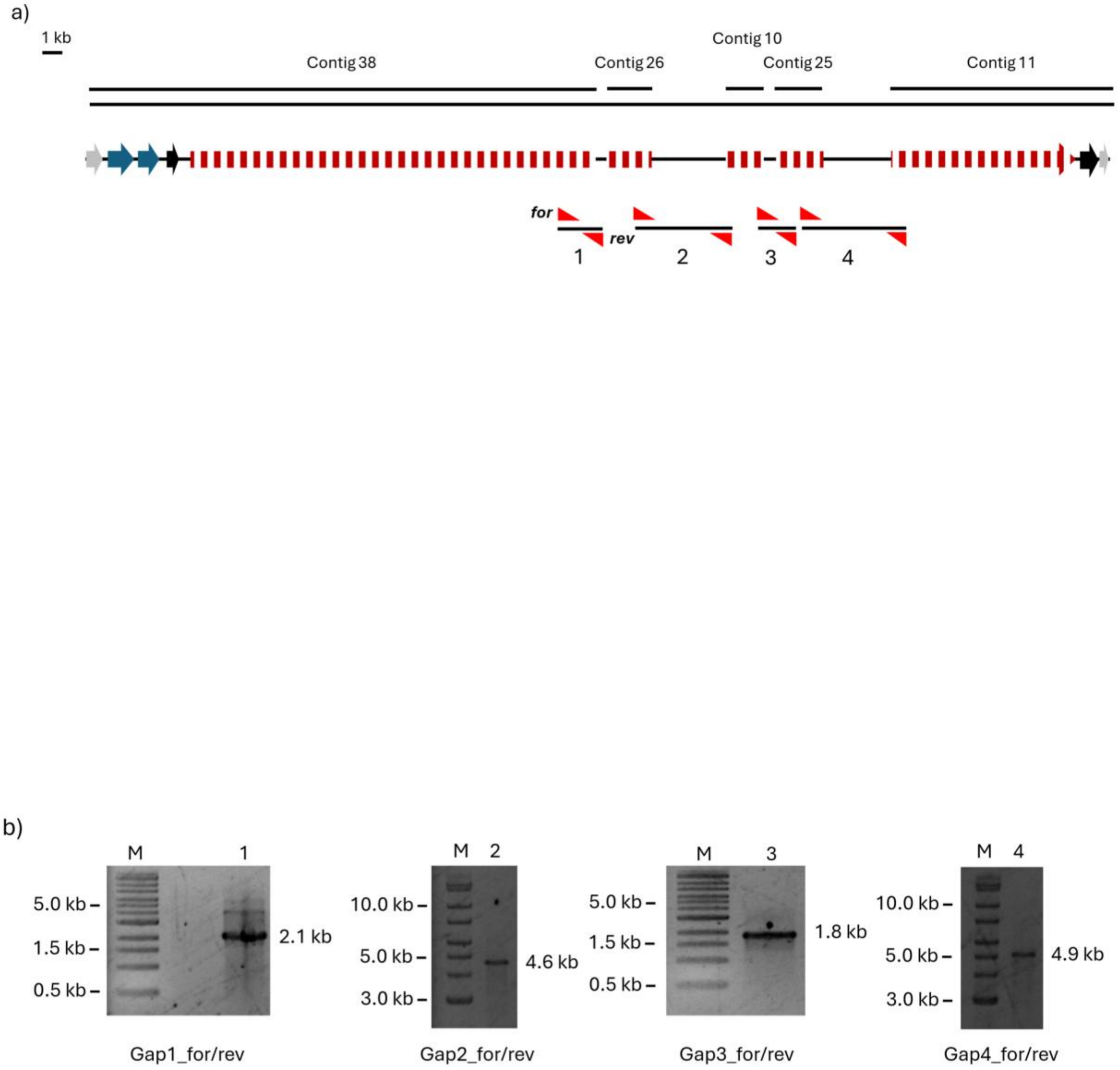

Figure S24. Gap closure of *P.* sp. P3_m182_1 *pdn* BGC. A) PCR-based strategy for closing the four potential gaps (1-4) within the gene cluster. Binding sites of the respective forward (f) and reverse (r) primers for each gap are indicated (red triangles). b) GoTaq-PCRs with the four primer pairs designed for gap closure (Gap1_for/rev to Gap4_for/rev). These experiments were independently repeated two times with similar results.

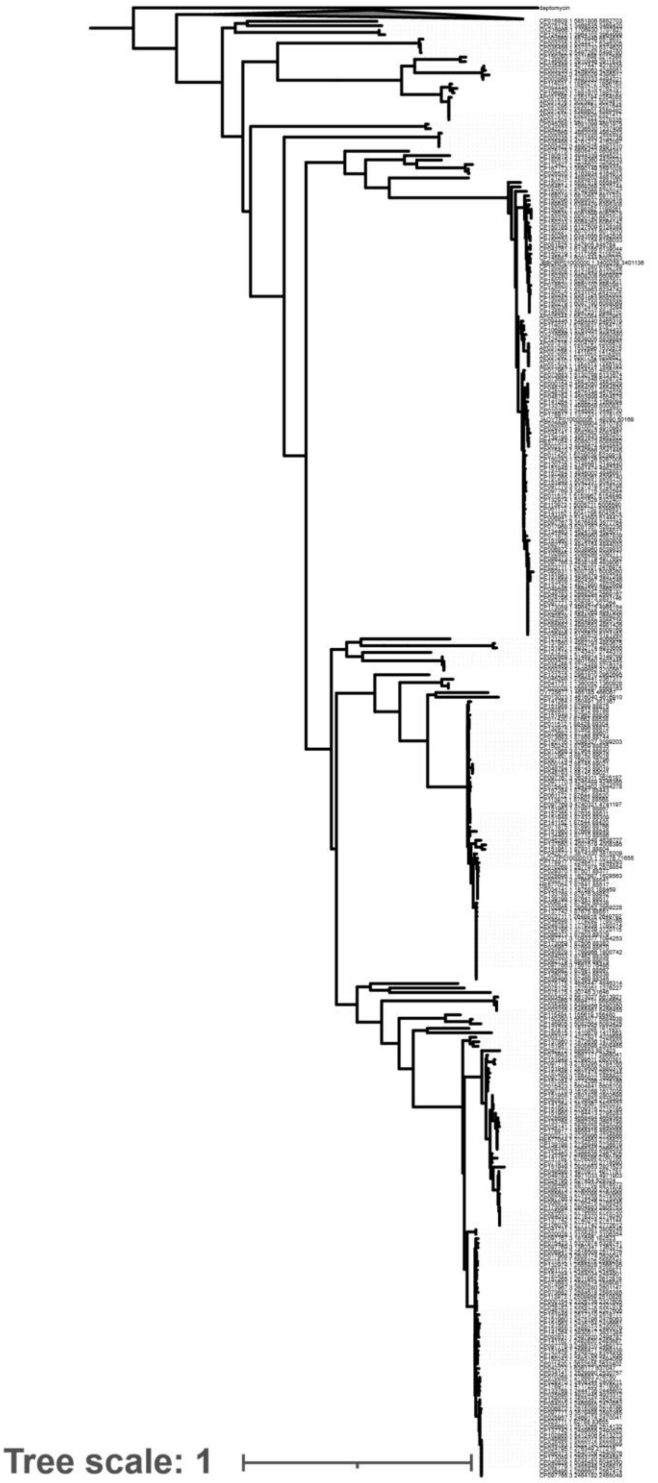

Figure S25. Phylogenetic tree using $C_{Starter}$ domains of whole-genome sequences of *Paenibacillus* spp.

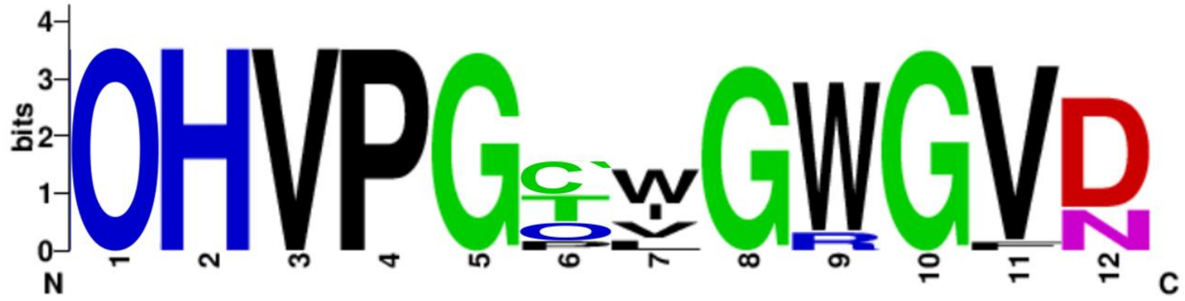

Figure S26. Sequence logo plot[12] of the predicted peptide sequences from all complete *pdn* BGCs detected by genome mining. One letter code abbreviation of amino acids (O: Ornithine, C': Citrulline). Letter size corresponds to prevalence in the predicted lipopeptide sequences.

```
M7+8          1 gcgcattatccgctgtcctcggcccaaaaacggctgtatgtcctgcagcagctggagggcgcagggctgagctacaacatgccggtggcgctgcggctcg   100
                   .||||.|||.|||||||||||||||||||||||||.||||||||||||||||.||.||.|.||||||||||||||||||||||||||||||.|||.|
M9+10         1 ---tattacccgttgtcctcggcccaaaaacggctgtacgtcctgcagcagctggaaggagccgagctgagctacaacatgccggtggcgctgcagctgg    97

M7+8        101 aaggagcgctggatcgggcgcggctggaagcggcgctgcaagcgctcatcgcccgtcatgaagcgctgcgcacttcgttcgccgtagtggacggcgaacc   200
                ||||||||||||||||||||||||||||||||||||||||||||||||||||||||||||||.||||||||||||||||.|||||.|||||||.|||.||
M9+10        98 aaggagcgctggatcgggcgcggctggaagcggcgctgcaagcgctcatcgcccgtcatgaatcgctgcgcacttcgtttgccgttgtggacgacgagcc   197

M7+8        201 ggtgcagcgggtgcaggaggagtggaccttctgcgtgtcctatgaggaagcggaggaaggggaagccgaggagcgtactcgtgcattcctccgcccgttt   300
                |||.||.||||||..||||.||..||..|||...||.||.|||||||||||||||||||||||||||||||||||.|.||||||||||||||.||||||
M9+10       198 ggtacaacgggtggcggagcaggtgatattcgaagtttcttatgaggaagcggaggaaggggaagccgaggagcgcattcgtgcattcctccgtccgttt   297

M7+8        301 gaccttgacgaggccccgctgctgcgtacaaccgtcgtgcgtctcggagaagcgcggcacttgctgctgttcgatatgcaccatattatctcggatggca   400
                |||||||||||||||||||||||||||||||||||||||||||||||||||||||||||||||||||||||||||||||||||||||.||||||.||||
M9+10       298 gaccttgacgaggccccgctgctgcgtacaaccgtcgtgcgtctcggagaagcgcggcacttgctgctgttcgatatgcaccatattgtctcggacggca   397

M7+8        401 cgtcgatcagcattttagtagacgaatttgtgaagctgtacgcgggcgaagcgttagagccgctgcagctgcagtacaaagattatgcggtatggcagcg   500
                ||||||||||||||||||||||||||||||||||||||||.||||||||||||||||||||||||||||||||||||||||||||||||.|||||||||||
M9+10       398 cgtcgatcagcattttagtagacgaatttgtgaagctgtatgcgggcgaagcgttagagccgctgcagctgcagtacaaagattatgcagtatggcagcg   497

M7+8        501 ggaacactatgccgacagccgggcatacgagcagttggaagcgtactggatggaccagtttgcgggcgaactgccggtgctgagcctgccggccgatcat   600
                |||||||||.||||||||||||||||||||||||||||||||||||||||||||||||||||||||||||||||||||||||||||||||||||||||
M9+10       498 ggaacactacgccgacagccgggcatacgagcagttggaagcgtactggatggaccagtttgcgggcgaactgccggtgctgagcctgccggccgatcat   597

M7+8        601 ccgcgtccggccgtacgcagcttcgagggcggccgggtggacgttgagctggacggcgagcttgcggcggccgtgtatgaactggcccgcacgagcggcg   700
                |||||||||||||||||||||||||||||||||||||||||||||||||||||||||||||||||||||||||||..||||.|||||||||||||||||||
M9+10       598 ccgcgtccggccgtacgcagcttcgagggcggccgggtggacgttgagctggacggcgagcttgcggcggccgtgcgtgagctggcccgcacgagcggcg   697

M7+8        701 ctacggtgtacatggtgctgctagccgcctacagcacgctgctggcgcggcttggcgggcaagaagaagtcattgtaggttcgccggtggcgggacggcc   800
                ||||||||||.|||||||||||.||||||||||||||||||||||||||||||||||||||||||||||||||||||.|||||||||||||||||||||||
M9+10       698 ctacggtgtatatggtgctgctggccgcctacagcacgctgctggcgcggcttggcgggcaagaagaagtcattgtcggttcgccggtggcgggacggcc   797

M7+8        801 gcaggcggagcttgaaggcatgctggggatgttcgtgaacaccttggcgctgcggacctacccggccggggagaagagctttgccgcctatttgcaggaa   900
                ||||||.|||||.||.||||||||||||||||||||||.|||||.|||||.|||||||||||||||||||||||||||||||||||.|||||||||||||||
M9+10       798 gcaggcagagctggagggcatgctggggatgttcgtaaacacattggcactgcggacctacccggccggggagaagagctttgctgcctatttgcaggaa   897

M7+8        901 gtgaagcagatggcgcttggcgccttcgagcacggggactatccattcgaggaactggtggaacgggtggcgcggcagcgcgacacgagccgcaacccga  1000
                |||||||||||||||||||||||||||||||||||||.||||||||||||||||||||||||||||||||||||||||||||||||||||||||||||||
M9+10       898 gtgaagcagatggcgcttggcgccttcgagcacgggggctatccattcgaggaactggtggaacgggtggcgcggcagcgcgacacgagccgcaacccga   997

M7+8       1001 tcttcgatgcgatgcttgtgctgcagaacatggatcaggcggagcttgagctccccgagcttcagctcacctcgtatccgttcgattccaacgtggccaa  1100
                ||||||||||||||||||||||||||||||||||||||||||||||||||||||||||||||||||||||||||||||||||||||||||||||||||||
M9+10       998 tcttcgatgcgatgcttgtgctgcagaacatggatcaggcggagcttgagctccccgagcttcagctcacctcgtatccgttcgattccaacgtggccaa  1097

M7+8       1101 gttcgatctgacgctgtcggtgagcgaacaggagaacggcatgcgctgctcgtgggaattcgctgccgtgttatttgaacgggcgacgatagagcgttgg  1200
                ||||||||||||||||||||||||||||||||||||||||||||||||||||||||||||||||||||||||||||||||||||||||||||||||||||
M9+10      1098 gttcgatctgacgctgtcggtgagcgaacaggagaacggcatgcgctgctcgtgggaattcgctgccgtgttatttgaacgggcgacgatagagcgttgg  1197

M7+8       1201 gccggtcactttgcggagcttctgcgccagattacccgggatccgcaggtgacgctgggaagcgtcagcttgctgacggctgcggaacaggaacaattgt  1300
                |||||||||||||||||||||||||||||||||||||||||||||||||||||||||||||||||||||||||||||||||||||||||||..|.|
M9+10      1198 gccggtcactttgcggagcttctgcgccagattacccgggatccgcaggtgacgctgggaagcgtcagcttgctgacggctgcggaacaggctcga----  1293

M7+8       1301 tgacgcaattccatgagttggggcagttcaatgata---gcgggacaaccgccgcgttgctgcgggacatgacgctgcacgcgctgttcgagcagcaggc  1397
                                 ||||.|||||||||.||.|   ||||      .||.|.|..||.||||||||..|||||||||||||||||||||||||||||
M9+10      1294 -----------------ttggcgcagttcaacgacaccggcgg------tgcagaggcgccgcgggacaccacgctgcacgcgctgttcgagcagcaggc  1370

M7+8       1398 ggcgaagacgccggagcggttggccgttgtatgcggcgacgattccctgacctaccgggaactgaacgaacgggcgaatcggcttgccagagtcgtgctc  1497
                ||||||||||||||||||.|||..|||||||||||||||||||.|||||||||||||||||.|||||||||||||||||.|||||||||||||.|||.|||
M9+10      1371 ggcgaagacgccggagcgcttgagcgttgtatgcggcgacgatgccctgacctaccgggagctgaacgaacgggcgaaccggcttgccagagccgtcctc  1470

M7+8       1498 cgctacggcgccgggcccgaaagccttgttgcggttcagtgcgaacgttccgtggatatggctattgcgctgctcgccgtactcaaggctggagctgcct  1597
                ||.|||||||||||||||||||||||||||||||.||.|||||||.||.||.|||||||||||.|||||||||||||||||.||||||||.||||||||||
M9+10      1471 cggtacggcgccgggcccgaaagccttgttgcagtgcagtgcgagcgatctgtggatatggcgattgcgctgctcgccgtgctcaaggccggagctgcct  1570

M7+8       1598 atctgccgatatcgccgcaagagccggcagagcgggtgcagttcctgctggaggacagcggcgcgaccgtactgctgtctgcctcggcacaggaggcttc  1697
                |||||||||||||||||||.|||||||||||||||.|||||||||||||||||||||||||||||||||.|.|||||||||.||.||||||||||||||.|
M9+10      1571 atctgccgatatcgccgcaggagccggcagagcggttgcagttcctgctggaggacagcggcgcgaccttgctgctgtcttccacggcacaggaggctgc  1670

M7+8       1698 ctgtccggtgctaggcttggatgatggccgaatcgagct-ggaatcgccggaaaatttggcgggctatgcgaaaccaaataatctggcgtatgtgatcta  1796
                |||.||||||.|..||||||||.|||...|.||| .|.| ||||.||||||||.||.|.|||.|||.||..||.||.||..|.|||||||||||||||||
M9+10      1671 ctgcccggtgatgcgcttggataatgattgtatc-cgttcggaaacgccggaagatctcgcgagctctgtaaatcccaaacacctggcgtatgtgatcta  1769

M7+8       1797 tacctccggaacttccggtcagccgaaaggcgtcatggtggagcatggaagcatcgttcatacgcttcagtggaaggcagaagcctacgacttcgacggc  1896
                .|||||||||||.||.|||||||||||||||||.||||||||||||.|.|||||||||||||||||||||||||..|||..||||||||||||||||||
M9+10      1770 cacctccggaacctcgggtcagccgaaaggcgttatggtggagcatcgcagcatcgttcatacgcttcagtggaaaacagcggcctacgacttcgacggc  1869

M7+8       1897 ggacgggtgctgcatgccagcccgttcatattcgatgcgttcatcacgcacttctttggccccctcgttgcgggcgccaccgtcgtgatgctgcgcgatg  1996
                ||.|||||||||||||||||||||||||.|.|||||||||||||||||||||||||||||||||||||.||||||||||||||||||||||||||||||||
M9+10      1870 gggcgggtgctgcatgccagcccgttcgtgttcgatgcgttcatcacgcacttctttggccccctcgctgcgggcgccaccgtcgtgatgctgcgcgatg  1969

M7+8       1997 aagagctcaaagatccggaggtcattatccgcacgctcgtcgagcagaagattacccacgcgcaattcccaacgggtctgctcgcggcgctgctggagat  2096
                |||||||.|..|||||||.||.||||||||||||||||.|.|||||||||||||||||||||||||||.|||.|.||||||||||||||||||.|||||||||||
M9+10      1970 aagagctaagcgatccggcggccattatccgcacgcttgccgagcagaagattacccacgcgcagttctcgacgggtctgctcgcggcactgctggagat  2069

M7+8       2097 gacccggcaggaacagctggccaccgtccggagcgtggtcaccggcggggaaaaaatcagtgcggcgctgattgaaaagatgcttgtttacagccagatt  2196
                ||||||||.||||||||||||||||||||||||||||||||||||||||||||.||||||||.||||||||.||.|||||||||||.|.|.||.|.|||||.
M9+10      2070 gacccggccggaacagctggccaccgtccggagcgtggtcaccggcggggagaaaatcagcgcggcgctaatcgaaaagatgctggctcaccgtcagatc  2169

M7+8       2197 gagtttgtcagcgagtatggaccgacggaaaacagcgtggtgacgaccgctttgccggtgaccgatccgggtcagtctcatacgctcggcaggtccattg  2296
                ||||||||||||||.||||||||.|||||.|||||||||||||||||||||||||||||||||||||||||||.||||.||||||||||||||||||||||||||||
M9+10      2170 gagtttgtcagcgaatatggaccaacggagaacagcgtggtgacgaccgctttgccggtgaccgatacggggcagtctcatacgctcggcaggtccattg  2269
```

```
M7+8       2297 gccagacgaaagttcatgtgctggcagcggatggacaactgcagccgatcggcgtcccgggcgagctgtgcatcagcggcccgggattggcccgggggta   2396
                ||||.|||||||||||||||||||||||||||||||||||||||||||||||||.|||||||||||||||||||||||||||||||||||||||||||||
M9+10      2270 gccaaacgaaagttcatgtgctggcagcggatggacaactgcagccgatcggcgtaccgggcgagctgtgcatcagcggcccgggattggcccgggggta   2369

M7+8       2397 tctgaatcagccggagctgacggccgaacggttcgtcgccagtccgttcgcgccagatgagcggctctatcggaccggggacttggcgcgctggctgccg   2496
                ||||||||||||||||||||||||||||||||||||||||||||||||||||||||||||||||||||||||||||||||||||||||||||||||||||
M9+10      2370 tctgaatcagccggagctgacggccgaacggttcgtcgccagtccgttcgcgccagatgagcggctctatcggaccggggacttggcgcgctggctgccg   2469

M7+8       2497 gatggcaatctcgaatatatgggccggatggacggacaagtgaagatccgggggtaccggatcgaaccgggcgagatcgaagcccagctgctgcagttga   2596
                ||||||||||||||.|||||||||||||||.|||||||||||||||||||||||||||||||||||||||||||||||||.||||||||||||||||||||
M9+10      2470 gatggcaatctcgagtatatgggccggatagacggacaagtgaagatccgggggtaccggatcgaaccgggcgagatcgaagcacagctgctgcagttga   2569

M7+8       2597 ccggggtaagcaaagccgtcgtggccgcgctgccggatgcttcaggcagtctccagctgtgtgcctatgttgttggcggcgagttggattttcctcagct   2696
                |||||||||||||||||||||||||||||||||||||||||||||.||||||||||||||||||||||||||.|....|||||||.|||||.|||||.|.||
M9+10      2570 ccggggtaagcaaagccgtcgtggccgcgctgccggatgcttcgggcagtctccagctgtgtgcctatgtcgcccccggcgagctggatcttcctgatct   2669

M7+8       2697 gcgcagcttgctgtcctctgcgctgccggcgtatatggttccggcttccttcgtgaagctggaccaattgccgctgactgccaacggcaaaatcgatttc   2796
                ||||||||||||||||||||.||||||||||||||||||||||.|.||||||||||||||||||||||||||||||||||||||||||||||||||||||
M9+10       2670 gcgcagcttgctgtcctctgtgctgccggcgtatatggttccgtcctccttcgtgaagctggaccaattgccgctgactgccaacggcaaaatcgatttc   2769

M7+8       2797 aaagcgttgccgaagccggatgcagctctggcccatgcgccgtatgccgctccccgcacgccggtggagcaggcgctggtgacggtatggcagcaggtgc   2896
                ||||||||||||||||||||||||||||||||||||||||||||||||||||||||||||||||||||||||||||||||||||||||||||||||||||
M9+10      2770 aaagcgttgccgaagccggatgcagctctggcccatgcgccgtatgccgctccccgcacgccggtggagcaggcgctggtgacggtatggcagcaggtgc   2869

M7+8       2897 tcggtgtgccgcaggtcggcattcatgacagcttcttcgacctgggaggcgactcgatcaaggcgattcaagcagcgtcccggctgctgccggccggtta   2996
                ||||||||||||||||||||||||||||||||||||||||||||||||||||||||||||||||||||||||||||||||||||||||||||||||||||
M9+10      2870 tcggtgtgccgcaggtcggcattcatgacagcttcttcgacctgggaggcgactcgatcaaggcgattcaagcagcgtcccggctgctgccggccggtta   2969

M7+8       2997 taagctggacatgaaggatctgttccgttatccggctgcggcccagctcagcgctcatatcgccccggttagccgtattgcggaacaggggggagattaac   3096
                ||||||||||||||||||||||||||||||||||||||||||||||||||||||||||||||||||||||||||||||||||||||||||||||||||||
M9+10      2970 taagctggacatgaaggatctgttccgttatccggctgcggcccagctcagcgctcatatcgccccggttagccgtattgcggaacaggggggagattaac   3069

M7+8       3097 ggaccggtgccgctcacgccgattcagcactggtttgccgagcgggatctggccgatgcgcatcacttcaaccaagccgtgatgctgtaccgggaagaac   3196
                ||||||||||||||||||||||||||||||||||||||||||||||||||||||||||||||||||||||||||||||||||||||||||||||||||||
M9+10      3070 ggaccggtgccgctcacgccgattcagcactggtttgccgagcgggatctggccgatgcgcatcacttcaaccaagccgtgatgctgtaccgggaagaac   3169

M7+8       3197 ggtttgacgtgcaagcgctgcgcctggcgatgaagcggatcacgattcatcacgacgcgctgcgcatggtgtttgtgccttcagcagatgggggttatgc   3296
                ||||||||||||||||||||||||||||||||||||||||||||||||||||||||||||||||||||||||||||||||||||||||||||||||||||
M9+10      3170 ggtttgacgtgcaagcgctgcgcctggcgatgaagcggatcacgattcatcacgacgcgctgcgcatggtgtttgtgccttcagcagatgggggttatgc   3269

M7+8       3297 cgcttggaaccgggccgtgaatgaaggggacctgtacggattggaagtattcgacttgcgggaagagcaggcgtggagccggttagtcgaagcgaaggct   3396
                ||||||||||||||||||||||||||||||||||||||||||||||||||||||||||||||||||||||||||||||||||||||||||||||||||||
M9+10      3270 cgcttggaaccgggccgtgaatgaaggggacctgtacggattggaagtattcgacttgcgggaagagcaggcgtggagccggttagtcgaagcgaaggct   3369

M7+8       3397 cgcgagctgcaggcaggtatctccttagatacaggtccgctcgtgcagctgggtttattccagtgcggtgacggcgatcatctgctcatcgtcattcacc   3496
                ||||||||||||||||||||||||||||||||||||||||||||||||||||||||||||||||||||||||||||||||||||||||||||||||||||
M9+10      3370 cgcgagctgcaggcaggtatctccttagatacaggtccgctcgtgcagctgggtttattccagtgcggtgacggcgatcatctgctcatcgtcattcacc   3469

M7+8       3497 atctcgtcattgatggcgtctcttggcggatcttgctggaagacctcgctgcagcgtacgaacaggccgcgcaagggcaggacatccggctgccgcagaa   3596
                ||||||||||||||||||||||.|||||||||||||||||.|||||||||||||||||||||||||||||||||||||||||||||||||||||||||||
M9+10      3470 atctcgtcattgatggcgtctcctggcggatcttgctggaggacctcgctgcagcgtacgaacaggccgcgcaagggcaggacatccggctgccgcagaa   3569

M7+8       3597 aaccgattcgttccaagtctggtcacagcagctggcagcttatgcctcaagccaggctatggaggcggaacgtgaatattggagccagatggcga--gtc   3694
                ||||||||||||||||..|||.|.||||||||||||||||||||||||||||||.||||||||||.|.||||||||..||||||..||..||||||  ||.
M9+10      3570 aaccgattcgttccaagcttgggcgcagcagctggcagcttatgcctcaagcccggctatggagaccgaacgtgggtattggaagcaactggcgaatgtt   3669

M7+8       3695 ggg--aacaagtgccattacctaaggataacgatcagggcggagacacggtcgaaaacagcgcaatgttgacgctgcaatggtcccggcgcgagacgcag   3792
                .||  ||||    ||||||||.|||||||||||||||||||||||||||||||||||||||||||||||||||||||||||||||||||||||||||||
M9+10      3670 aggctaaca----ccattaccgaaggataacgatcagggcggagacacggtcgaaaacagcgcaatgttgacgctgcaatggtcccggcgcgagacgcag   3765

M7+8       3793 cggctgttaaccgaagcgcaccgggcatatgggacagagattaacgatctgctgctgaccgcgcttgggatggcgattcaggagtggaccggaagcgagc   3892
                ||||||||||||||||||||||||||||||||||||||||||||||||||||||||||||||||||||||||||||||||||||||||||||||||||||
M9+10      3766 cggctgttaaccgaagcgcaccgggcatatgggacagagattaacgatctgctgctgaccgcgcttgggatggcgattcaggagtggaccggaagcgagc   3865

M7+8       3893 aggtggccgtcatgctggaagggcacggcagggaatccatcctgccgaatgtggacgtcagccggacggtaggctggttcacgagtgcatatccggtcat   3992
                ||||||||||||||||||||||||||||||||||||||||||||||||.|||||||.||||||||||||||||||||||||||||||||.||.||||||||
M9+10      3866 aggtggccgtcatgctggaagggcacggcagggaatccatcctgccggatgtggatgtcagccggacggtaggctggttcacgagtgcgtacccggtcat   3965

M7+8       3993 gctggatatgaaagcgaacctggatctgccgcggcgcatcaagctggtcaaggaaacgctgcgaagcatcccgcacaaaggcgttggttacggtattttg   4092
                |||.||.|||||..||.||.||||.||||||||||||||||||||||||||||||||||||||||.||||||||||||||||||.|.||.|||||.||||||
M9+10      3966 gcttgacatgaagtcggacttggacctgccgcggcgcatcaagctggtcaaggaaacgctgcggagcatcccgcacaaaggcatcggctacggcattttg   4065

M7+8       4093 cgctatttgg---cttcgcctaa------tattcaagcggcc----gcacctgaaataacgtttaactatttggggcagttcgaccaggatttgcagcgc   4179
                ||.|||.|||   |.||||.|.|      |||    .|||.|     |||||||||||..|||||||||||||||||||||||||||||||||||||||||
M9+10      4066 cgttatctggcctcgtcgcgtgaggacggtat----ccggacgttggcacctgaaatttcgtttaactatttggggcagttcgaccaggatttgcagcgc   4161

M7+8       4180 agcgcgcttcagatctcgccgcttgcgtccggggatgcggtaagccgcaggcaaaaaaagggcggccacgctcgatttcagcggcatgattgccggaggag   4279
                |||.|||||||||||||||||||||||.|||||||||||||||||||||||||||||||||||||||||||||||||||||||||||||||||||||.
M9+10      4162 agctcgcttcagatctcgccgcttgcgtctggggatgcggtaagccgcaggcaaaaaaagggcggccacgctcgatttcagcggcatgattgccggaggaa   4261

M7+8       4280 cgttgagcctgaacctgagctacagcatggagcagtaccgcaagaaaaccatgcagcagttggtcaagcgactaaagcatcatttgcaggaagtgattcg   4379
                |||||||||||||.||||||||||||...|||||||||||||||||||||||||.||||||||.|.||||.||.|||||||||||||||||||||||||||
M9+10      4262 cgttgagcctgaatctgagctacagcgccgagcagtaccgcaagaaaaccatgcggcagttggcccagcggctgaagcatcatttgcaggaagtgattcg   4361

M7+8       4380 tcactgcgtggcgaaagaaagcgcggaactgacgccaagcgatgtgctgcagcagggcatgacgattgaggagctggaagcgctggttgagcggaatcgg   4479
                ||||||||||||||||||||.||.||.|||||||||||||||.|||||||.|||||.||||||.|||||||||||||||||||||||||||.|.||||
M9+10      4362 tcactgcgtggcgaaagaaagggcagagctgacgccaagcgatctgctgcatcaggggatgacgcttgaggagctggaagcgctggttgagcgtactcgg   4461
```

```
M7+8      4480 attgtcggggacgtcgagaatgtatatgccttgacgcctatgcagcaggggatgctgttccatagccgcctggatcctcagtcgggcgcttacgtgaacc  4579
               ..|.|.||||||.|||||||.|||||||||.|||||||.|||||||||||||||||||||||||||||||||||||||||||||||||||||||||||||
M9+10     4462 cctcttggggacatcgagaacgtatatgccctgacgccaatgcagcaggggatgctgttccatagccgcctggatcctcagtcgggcgcttacgtgaacc  4561

M7+8      4580 aaatgctcatttcgctgcagggaaaactagatccaaccgcgttggaacgcagctggaatatggtcatccagcggcatgcggtattgcggacgagtgtcga  4679
               ||||||.|||||||||||||||||..||..|||.|.|||||||.||||||||||||||||||||||||||||||||||||||||||||||||||||||||
M9+10     4562 aaatgcacatttcgctgcagggaaggctgaatctagccgcgtttgaacgcagctggaatacggtcattcagcggcatgcggtactgcgtacgagcgtaga  4661

M7+8      4680 cagcggttggcgggatcagccgctgcaagtagtctatcgtagccgtagcttgaggattgcctattcagacatatcgatgaagagtacggcggagcaggag  4779
               |||||||||||||||.|||||||||||.||.||||..||..||||.|.||||||||||||||||.|.||..|.|||||||||||.||.||.||||||||.
M9+10     4662 cagcggttggcgggaccagccgctgcaggtggtcttccgccgccgcaccttgaggattgcctatgcggatctgtcgatgaagagcacagccgagcaggaa  4761

M7+8      4780 ctggaattggccttcctcaaggaagaagaccgtaaaaaaggctttgcggtagagtcggacagcttgatgcgtgtggcggtcgtgcgtacggggccagaga  4879
               ||||||.||||||||.|||.|||.||||||||||||||.|||||||.|.|.|||||||.||||.|.|||||.|||..|||.||.||||||||||.|||||
M9+10     4762 ctggaactggccttcatcatggatgaagaccgtaaaaagggctttgtgttggagtcggccagcctaatgcgcgtgaaggtggtacgtacggggcaagaga  4861

M7+8      4880 cgcaccaactgttgtggagcttccaccatattctcatggatggatggtgtctgccgctggtggtcaaggaagtcctcgaggtatacacggccttgtgcaa  4979
               ||.||||.|||.|||||||.||||||||||||||.||||||||||||||.|||||||||||||||||||||||.||.||.||||||||||||||||||||
M9+10     4862 cgaaccagctgctgtggagtttccaccatattctgatggatggatggtgcctgccgctggtggtcaaggaagttctggaagtatacacggccttgtgcaa  4961

M7+8      4980 ggaagaggagccgcaattgccgcgcgtgggcgagtatagcgagtatattcgctggctggccgagcaggacgggcaagcggcctcgaactattgggcggac  5079
               ||||||||||||||||||||||||||||.|||||||.||||||||||||||||||.|||||||||||||.|||||||||||.||||||||||||||||||
M9+10     4962 ggaagaggagccgcaattgccgcgcgtgagcgagtacagcgagtatattcgctggttggccgagcaggatgggcaagcggcgtcgaactattgggcggac  5061

M7+8      5080 atgcttgcgggcttcgaacaagcggccgagcttccaagacggcagcgccaaacacagggatatgaagcaaggcgcgtgacctgctcactggatcggaagc  5179
               ||||||||||||||||||||||||||||||||.||.|||||||||||||||.||||||||||||||||.|.|||.|||||||||||.||||||.||||||
M9+10     5062 atgcttgcgggcttcgaacaagcggccgagctcccgagacggcagcgccaagcacagggatatgaagcgatgcgtgtgacctgctcgctggaccggaagc  5161

M7+8      5180 ggaccgaacggatcagccaagcagcgcgagagcagggagtgacggtgaataccttgctgcagacggcatggggactgctgctgcacaaatacagcgggac  5279
               ||||||||||||||||||||||||||||||||||||||||||||||||||||||||||||||||||||||||||||||||||||||||||||||||||||
M9+10     5162 ggaccgaacggatcagccaagcagcgcgagagcagggagtgacggtgaataccttgctgcagacggcatggggactgctgctgcacaaatacagcgggac  5261

M7+8      5280 agacgatgcggtattcggcagcgtggtgtctggcagaccggcgagccttcctggcgtggaggggatgatcgggctgttcatcaatacgattccgattcgg  5379
               ||||||||||||||||||||||||||||||||||||||||||||||||||||||||||||||||||||||||||||||||||||||||||||||||||||
M9+10     5262 agacgatgcggtattcggcagcgtggtgtctggcagaccggcgagccttcctggcgtggaggggatgatcgggctgttcatcaatacgattccgattcgg  5361

M7+8      5380 gtgaagtgcgaggctggagaaacggtagcccgcgtgctgcagcagatccaagagcaagcgctggcgtcccaagcctgcgactattatccgctgcatgaaa  5479
               ||||||||||||||||||||||||||||||||||||||||||||||||||||||||||||||||||||||||||||||||||||||||||||||||||||
M9+10     5362 gtgaagtgcgaggctggagaaacggtagcccgcgtgctgcagcagatccaagagcaagcgctggcgtcccaagcctgcgactattatccgctgcatgaaa  5461

M7+8      5480 tccaggcccaaagcgcaaggcaccgggagttgttcaaccacattctggtgttcgaaaactacccggtggaggagcaagcagattcactggaagatgcggc  5579
               |||||||||||||||||||||||||||||.|||||||||||||.|||||||||||.|||||||||||||||||||||||.|.||||||||.||||.||||
M9+10     5462 tccaggcccaaagcgcaaggcaccgggagctgttcaaccacatcctggtgttcgagaactacccggtggaggagcaagcgggttcactgggagatacggc  5561

M7+8      5580 gggactaaagattaccggagttcaagcggaagaacggaccaactatgacttgaatgtgatgattttgcccggggaagagatgacgcttcattttgactac  5679
               .|||||.|||||||||||||||||||||||||||||||||||||||||.||||||||||||||||||||||||.||||||||||.||.||||||||||||
M9+10     5562 cggactgaagattaccggagttcaagcggaagaacggaccaactatgatttgaatgtgatgattttgcccggagaagagatgaccctccattttgactac  5661

M7+8      5680 aatgcccaggtgtatgaacgagatggaatggagcgtctgcaaaggcatctgctgcaaatcgtggagcagattgcagcggactcgaacattccggtgcagg  5779
               |||||.||||||||||||||||||||.||||||||||||||||||||||||.|||.|||||||||||||||.||||||||.|||||||||||||||||.|
M9+10     5662 aatgcgcaggtgtatgaacgagatgggatggagcgtctgcaaaggcatctgatgcgaatcgtggagcagatcgcagcggattcgaacattccggtgcaag  5761

M7+8      5780 agctggaactgctgacaacggacgagagagagcagttactcgtgcaattcaacgatacggtagcggaa-------attcc--aagggg--agcgtccgtg  5868
               |||||||||||||||||||.|.|||||||||||||.|.||||||||.|||||||||||       |||       |.|||  |||.||  |||.||
M9+10     5762 agctggaactgctgacaacagccgagagagagcagctgctcgtgcagttcaacgatac-------gaaggttatgactcctgaagaggcaagcatc----  5850

M7+8      5869 cattccttgttcgagcagcaggcggcgaaaacgccggatcgtccggcggtcgtgtgcgcagaacagcggctgacctaccgggagctggaggaacaggcca  5968
               |.||||.|||||||||||||||||||||||||||||.|||.||||||.||||||||||||||||||||.|||||||||.||||||||||||||||.|||
M9+10     5851 ccttccctgttcgagcagcaggcggcgaaaacgccgaatcatccggcagtcgtgtgcgcagaacagcgtctgacctaccaggagctggaggaacagacca  5950

M7+8      5969 accgtatcgcccaatggctgcgggcgcacggcgtgacggcggaagagcgtgtaggcgtcctgatgaaccgttcgccgcagctcatcgccgggctgctggg  6068
               ||||||||||.||||||||||.||||||||||||||||||||||||||||||||||||||||||||||||||||||||||||||||||||||||||||||
M9+10     5951 accgtatcgcgcaatggctgctggcgcacggcgtgacggcggaagagcgtgtaggcgtcctgatgaaccgttcgccgcagctcatcgccgggctgctggg  6050

M7+8      6069 gattctgaaagccggtgccgcttacgtgccgatggaccccccttctgcctgtggagcgcatcgaggggatgatccgggatgcaggaatgcaggttatgctg  6168
               ||||.|||||||||||||||||||.||||||||.||.||..||||||||..||||||.||.||||.|||||||||||||.|.||.||||||||.||||||
M9+10     6051 gattttgaaagccggtgccgcttatgtgccgatcgatccaattctgcctaaggagcgtatggaggcgatgatccgggattccgggatgcaggtaatgctg  6150

M7+8      6169 gcggattcggctcagcttgggat-tgtgg---------caggcctgcaggagacagcactgggccatgtattgtgtctggacgatgcgttgacggatgga  6258
               .|||||.|.||          || |||||         |.|.|.|..|.||||||.|.|||.|.||||.|||.|||.|.|||||
M9+10     6151 acggatgctgc----------atatgtggatacattatccgacttcaaagagacatcgctgtgtcatgcattatgtgtcgacga----------------  6224

M7+8      6259 gcggatagagcggctgggcagccggagcaagcagacacggcaagcctcggcggccat--actatctcgttctccttcgccaaggagctcgcaggatattc  6356
                                                                    ||||  |     |||.|.|||                  ||.|||||
M9+10     6225 -----------------------------------------------------ccatgaa-----tcgctgtcc------------------gggtattc  6248

M7+8      6357 ttccgagcctgttggcgtcgaggtagagccatccggcagtgcgtatgtcatctacacctccggaacgaccggcacgccgaaaggagtagtcgtggagcac  6456
               ||||||||||||||||||.||||.||.|||.||||||||.||.|||||||||||.||||||||.||||||||||||||||||||.|||||||||||||||
M9+10     6249 ttccgagcctgttggcgtggagataaagccttccggcagcgcatatgtcatctatacctccgggacgaccggcacgccgaaaggggtagtcgtggagcac  6348

M7+8      6457 cggaacgtggtgaatttcatctcggggatgattcgcgagcttcctttcgggacgcatgcttcgatgctgtgcgtaacgacggtatccttcgacatcttcg  6556
               ||||||||.||.|||||||||||.||.|||||.|||||||||||.|||||||||||||.|||||||||||||.|.||||||||.||||||||||||||||
M9+10     6349 cggaacgtagtcaatttcatctcaggaatgatccgcgagcttccgttcgggacgcatgattcgatgctgtgcatcacgacggtgtccttcgacatcttcg  6448

M7+8      6557 ccacggagagctgggtgccgctcagctgcggcatgcagatcgtcctcgcaagcgaggaagcgcagcaggatccggtcgtgctggcggaactgttggctca  6656
               ||.|||||||||||||.||||||||||||||||||||||||||||||||||||||.|||||||||||||||||||.||||||||||||||||||||||||
M9+10     6449 ccgcggagagctgggtaccgctcagctgcggcatgcagatcgtcctcgcaagcgaagaagcgcagcaggatccggccgtgctggcggaactgttggctca  6548

M7+8      6657 gcacccggtgcagatgatgcagacgacgccttcgcggctcatgctgctgctcggcgatgcgcggagcgcggcgcagctgcggaacatggagatgctgctg  6756
               .|||||||||||||||..|||||.||||||||||||.|||||||||||||||||||||||||||||.|||||||||||||||||||||||||.|||||||
M9+10     6549 acacccggtgcagatggcgcagatgacgccttcgcgtctcatgctgctgctcggcgatgcgcggagtgcggcgcagctgcggaacatggagacgctgctg  6648
```

```
M7+8       6757 gtcggcggggaagccttcccttccaccctctatgggcagctgagggaacatacggatgcggcgctgtacaacatgtacgggccgacggaaacgaccgtct   6856
                |||||.|||||||||||||||||||||||.|||...|||||||||||||||||.|||||||...||||||||||||||||||||||||||||||||||||
M9+10      6649 gtcggaggggaagccttcccttccaccctgtataaacagctgagggaacatacagatgcggaattgtacaacatgtacgggccgacggaaacgaccgtct   6748

M7+8       6857 ggtccacgttcgaccgcctggaaggggaagaacggatcggcatcggacggccgatggcgaacacgcaggtgtatgtggtgaacgacgcgctgcagccgca   6956
                |||||||.|||||||||.|.||||||||.||||||||.|.|||||||||.||||||||||||||||||||||||||||||||.||..|||||||||||||
M9+10      6749 ggtccacattcgaccgcttagaaggggacgaacggattgccatcggacgtccgatggcgaacacgcaggtgtatgtggtgaatgagtcgctgcagccgca   6848

M7+8       6957 gcccgttggcgtagccggtgaactgtgcatcggcggcgcggggtcgcccgcggatattgggcgcgtccgcagctgacggccgagaaatttgtggacagc   7056
                |||..|||||||.||.||.||.|||||||||||||||||.||||||||||||||||||||||||||||||||||||||||||||||||||||||||||||
M9+10      6849 gccgattggcgtggcaggcgagctgtgcatcggcggcgctggggtcgcccgcggatattgggcgcgtccgcagctgacggccgagaaatttgtggacagc   6948

M7+8       7057 ccgtttgtttccggggagcggctgtaccggaccggcgatttggcgcgctggctgccggacggccggctggagcatctggggcggatcgaccatcaggtaa   7156
                |||||||||||||||||||||||||||||||||||||||||||||||||||||.||.|||||||||||||||||||||||||||||||||||||||||||
M9+10      6949 ccgtttgtttccggggagcggctgtaccggaccggcgatttggcgcgctggctacctgacggccggctggagcatctggggcggatcgaccatcaggtaa   7048

M7+8       7157 agatccgggggtaccggattgaactcggtgaaatcgaagcccggttgctccacattccgggtgtaagcgaagccgtcgtgactgcagcagaaatgggtgc   7256
                ||||||||||||||||||||||||||||||||||||||||||||.||||.|||||||||||||||||||||||||||||||||||.||.|||||||.||.
M9+10      7049 agatccgggggtaccggattgaactcggtgaaatcgaagcccggctgctgcacattccgggtgtaagcgaagccgtcgtgactgccgcggaaatggatga   7148

M7+8       7257 gcaaacgcaggaattgtgcgcttatgtgacaggcgagcgtccgcttgcggcggccgagctgcgtgccgccctgactgcctcgctgccgtcgtacatgctg   7356
                .||.|||||.||||||.|||||||||||||||||||.||||.||||||||||||||||||||||||||||.||.|.||.||||||||||||||||||.||
M9+10      7149 ccatacgcacgaattgcgcgcttatgtgacaggcgatcgtctgcttgcggcggccgagctgcgtgccgccttggccgcatcgctgccgtcgtacatgatg   7248

M7+8       7357 ccgacgcacttcatgcagctggaagaactgccgctgacgccgaacggcaaggtggaccgccgggcgctgccgcagccttcgggaagcggagcggcagccg   7456
                |||.||||.|||||||||||||||||||||||||||||.|||||||||||||||||.||||||||.|||||||.|||.||||||||||||||||||||||
M9+10      7249 ccggcgcatttcatgcagctggaagaactgccgctgacaccgaacggcaaggtggatcgccgggcactgccgaagccgtcgggaagcggagcggcagccg   7348

M7+8       7457 gatatacagcgccgcgaaccgaagcagaggcgaagctggcgctaatgtggcaggaggtgctgggcgcctcgcgcgtaggcatccacgacaacttcttcga   7556
                ||||||||||||||||||||||.|.||||||||||||||||||..|||||||||||||||||||||||||||||||||||||||||||.|||||||||||
M9+10      7349 gatatacagcgccgcgaaccgatgtagaggcgaagctggcgctgttgtggcaggaggtgctgggcgcctcgcgcgtaggcatccacgataacttcttcga   7448

M7+8       7557 gctgggcggccattccttgaaggcgatgacgctcgtatcgcggattcaccaggcgctgggagtagagctgccgctgaggcagctgttcctctcgccgacg   7656
                ||||||||||||||||||||||||||||||||||||||||..||||||.|||||.||||||||||||||||||||.||.|||||||||||||||||||||
M9+10      7449 gctgggcggccattccttgaaggcgatgacgctcgtatcgaagattcatcaggcactgggagtagagctgccgctaagacagctgttcctctcgccgacg   7548

M7+8       7657 gttgaagggctggcggctgcgctggacgcg   7686
                ||.||||||||..|||||||||||.||||.
M9+10      7549 gtcgaagggctaacggctgcgctgaacgct   7578
```

Figure S27. Emboss Needle Alignment of modules 7+8 and 9+10 from the *pdn* BGC of *P. apiarius* MW-14. The two modules exhibit 91.2% sequence identity (2.5% gaps).

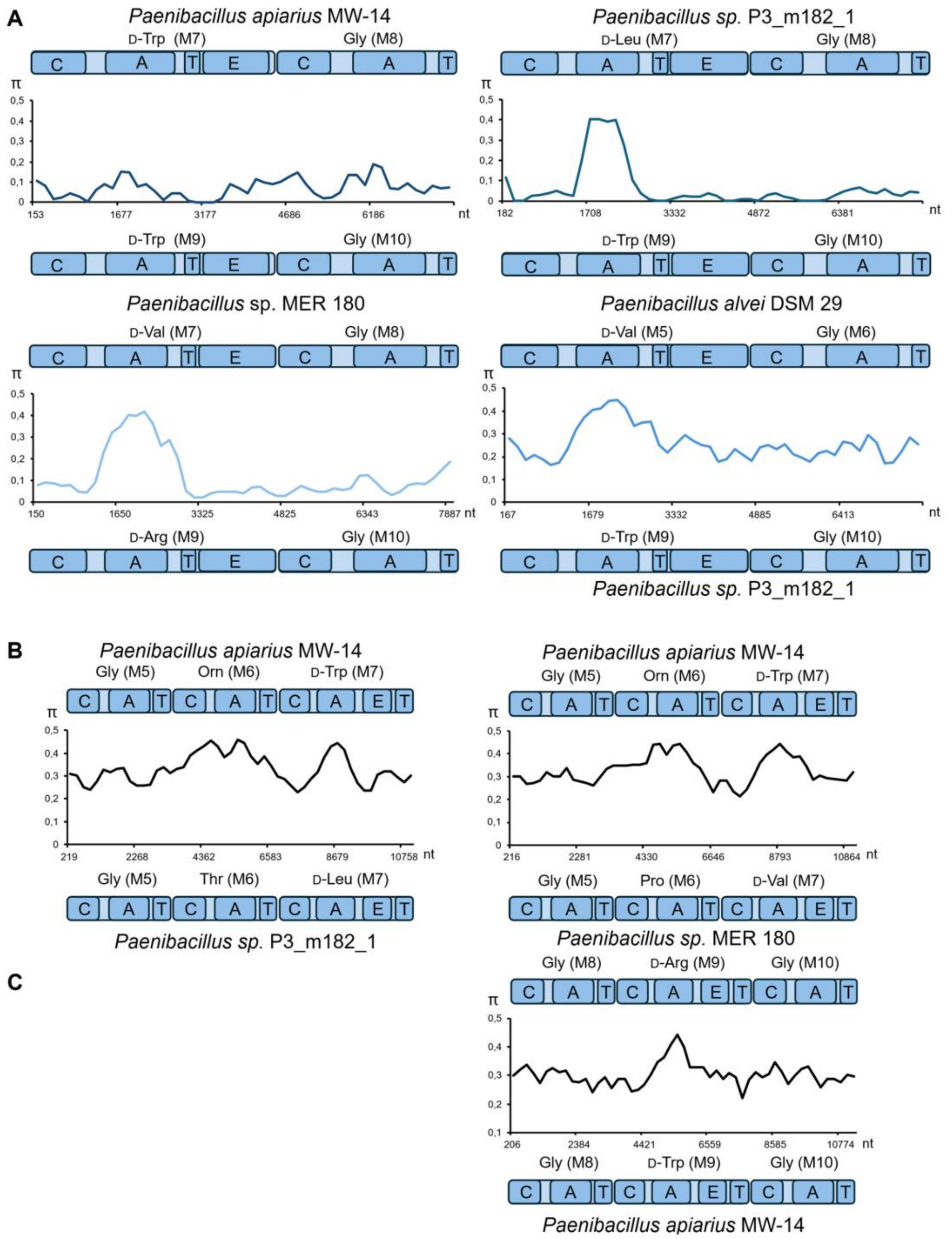

Figure S28. Sliding window analysis to identify regions of high sequence divergence, possibly originating from recombination in different *pdn* BGC encoding *Paenibacillus* strains. π values (average number of nucleotide differences per site between two sequences) were computed using the sliding window mode in DnaSP (width, 300 nt; step, 150 nt). (A) Sliding window analysis focusing on position 7 of the paenidepsins. (B) focusing on position 6 and (C) focusing on position 9.

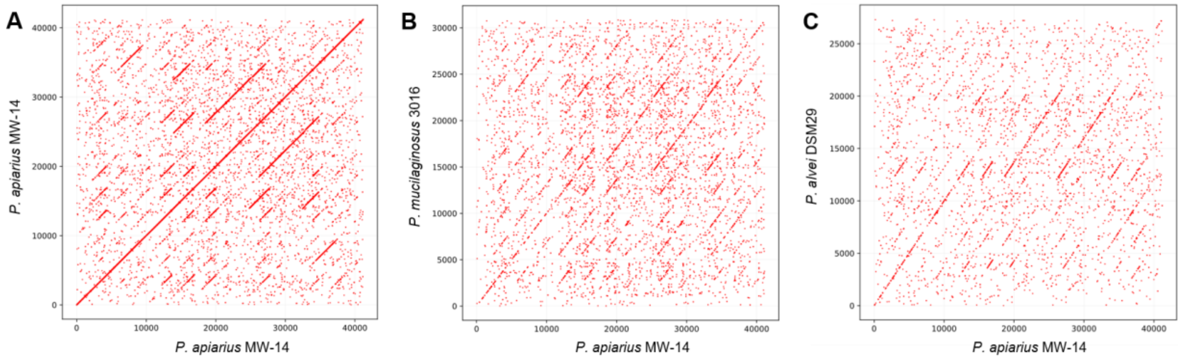

Figure S29. Dot plot analysis of the nucleotide sequence of the NRPS genes in *pdn* BGCs to detect loss or deletion of modules. (A) Dot plot analysis of *P. apiarius* MW-14 *pdn* BGC. (B) Dot plot analysis of *P. apiarius* MW-14 *pdn* BGC against the *pdn* BGC of *P. mucilaginosus* 3016 that contains 9 modules instead of 12. (C) Dot plot analysis of *P. apiarius* MW-14 *pdn* BGC against the *pdn* BGC of *P. alvei* DSM 29 that contains 8 modules instead of 12. Sequence deletion indicated by a right shift of the main diagonal is apparent for B and C. Window size of 10 and mismatch limit of 0.

```python
from Bio import SeqIO
from Bio.SeqFeature import FeatureLocation

# Input and output file names
input_gbk = "Example.gbk"  # Adjust filename
output_fasta = "condensation_starter_extracted.fasta"

# List to store extracted sequences
cs_sequences = []

# Parsing GenBank file
with open(input_gbk, "r") as handle:
    for record in SeqIO.parse(handle, "genbank"):
        for feature in record.features:
            if feature.type == "region":  # Check if feature is a biosynthetic cluster
                qualifiers_str = str(feature.qualifiers)

                # Check if region is an NRPS or NRPS-like cluster
                if "NRPS" in qualifiers_str or "NRPS-like" in qualifiers_str:
                    cluster_start = feature.location.start
                    cluster_end = feature.location.end
                    amp_binding_count = 0

                    # Count number of AMP-binding domains within this cluster
                    for sub_feature in record.features:
                        if sub_feature.type == "aSDomain" and "AMP-binding" in str(sub_feature.qualifiers):
                            if cluster_start <= sub_feature.location.start <= cluster_end:
                                amp_binding_count += 1

                    # If cluster has at least 5 AMP-binding domains, search for Condensation_Starter
                    if amp_binding_count >= 5:
                        for sub_feature in record.features:
                            if sub_feature.type == "aSDomain" and "Condensation_Starter" in str(sub_feature.qualifiers):
                                # Ensure the Condensation_Starter domain is within the cluster
                                if cluster_start <= sub_feature.location.start <= cluster_end:
                                    if isinstance(sub_feature.location, FeatureLocation):
                                        # Extract the Condensation_Starter sequence
                                        cs_sequence = sub_feature.extract(record).seq.translate()
                                        start = sub_feature.location.start
                                        end = sub_feature.location.end

                                        # Store extracted sequence
                                        header = f">{record.id}_{start}_{end}"
                                        cs_sequences.append(f"{header}\n{cs_sequence}\n\n")

# Save extracted sequences to FASTA file
if cs_sequences:
    with open(output_fasta, "w") as fasta_out:
        fasta_out.writelines(cs_sequences)
    print(f"{len(cs_sequences)} Condensation_Starter sequences saved in '{output_fasta}'!")
else:
    print("No Condensation_Starter sequences found.")
```

Figure S30. Python script's scheme for extracting $C_{Starter}$ amino acid sequences from NRPS and NRPS-like BGCs.

Supplementary references

(1) Kai, H.; Yamashita, M.; Takase, S.; Hashimoto, M.; Muramatsu, H.; Nakamura, I.; Yoshikawa, K.; Ezaki, M.; Nitta, K.; Watanabe, M.; Inamura, N.; Fujie, A. KB425796-A, a Novel Antifungal Antibiotic Produced by Paenibacillus Sp. 530603. *J. Antibiot. (Tokyo)* **2013**, *66* (8), 465–471. DOI: 10.1038/ja.2013.63.

(2) Kai, H.; Yamashita, M.; Takase, S.; Hashimoto, M.; Muramatsu, H.; Nakamura, I.; Yoshikawa, K.; Kanasaki, R.; Ezaki, M.; Nitta, K.; Watanabe, M.; Inamura, N.; Fujie, A. Identification of Ten KB425796-A Congeners from Paenibacillus Sp. 530603 Using an Antifungal Assay against Aspergillus Fumigatus in Combination with Micafungin. *J. Antibiot. (Tokyo)* **2013**, *66* (8), 473–478. DOI: 10.1038/ja.2013.64.

(3) Fu, C.; Keller, L.; Bauer, A.; Brönstrup, M.; Froidbise, A.; Hammann, P.; Herrmann, J.; Mondesert, G.; Kurz, M.; Schiell, M.; Schummer, D.; Toti, L.; Wink, J.; Müller, R. Biosynthetic Studies of Telomycin Reveal New Lipopeptides with Enhanced Activity. *J. Am. Chem. Soc.* **2015**, *137* (24), 7692–7705. DOI: 10.1021/jacs.5b01794.

(4) Xie, Y.; Wang, B.; Liu, J.; Zhou, J.; Ma, J.; Huang, H.; Ju, J. Identification of the Biosynthetic Gene Cluster and Regulatory Cascade for the Synergistic Antibacterial Antibiotics Griseoviridin and Viridogrisein in *Streptomyces Griseoviridis*. *ChemBioChem* **2012**, *13* (18), 2745–2757. DOI: 10.1002/cbic.201200584.

(5) Houwaart, S.; Youssar, L.; Hüttel, W. Pneumocandin Biosynthesis: Involvement of a *Trans* -Selective Proline Hydroxylase. *ChemBioChem* **2014**, *15* (16), 2365–2369. DOI: 10.1002/cbic.201402175.

(6) Mattay, J.; Houwaart, S.; Hüttel, W. Cryptic Production of *Trans* -3-Hydroxyproline in Echinocandin B Biosynthesis. *Appl. Environ. Microbiol.* **2018**, *84* (7), e02370-17. DOI: 10.1128/AEM.02370-17.

(7) Planckaert, S.; Deflandre, B.; de Vries, A.-M.; Ameye, M.; Martins, J. C.; Audenaert, K.; Rigali, S.; Devreese, B. Identification of Novel Rotihibin Analogues in *Streptomyces Scabies* , Including Discovery of Its Biosynthetic Gene Cluster. *Microbiol. Spectr.* **2021**, *9* (1), e0057121. DOI: 10.1128/Spectrum.00571-21.

(8) Li, J.-H.; Cho, W.; Hamchand, R.; Oh, J.; Crawford, J. M. A Conserved Nonribosomal Peptide Synthetase in *Xenorhabdus Bovienii* Produces Citrulline-Functionalized Lipopeptides. *J. Nat. Prod.* **2021**, *84* (10), 2692–2699. DOI: 10.1021/acs.jnatprod.1c00573.

(9) Yin, X.; Zabriskie, T. M. The Enduracidin Biosynthetic Gene Cluster from Streptomyces Fungicidicus. *Microbiology (N. Y.)* **2006**, *152* (10), 2969–2983. DOI: 10.1099/mic.0.29043-0.

(10) Ngoka, L. C. M.; Gross, M. L. A Nomenclature System for Labeling Cyclic Peptide Fragments. *J. Am. Soc. Mass Spectrom.* **1999**, *10* (4), 360–363. DOI: 10.1016/S1044-0305(99)00006-9.

(11) Shin, I.; Wang, Y.; Liu, A. A New Regime of Heme-Dependent Aromatic Oxygenase Superfamily. *Proc. Natl. Acad. Sci. U. S. A.* **2021**, *118* (43), e2106561118. DOI: 10.1073/pnas.2106561118.

(12) Crooks, G. E.; Hon, G.; Chandonia, J.-M.; Brenner, S. E. WebLogo: A Sequence Logo Generator. *Genome Res.* **2004**, *14* (6), 1188–1190. DOI: 10.1101/gr.849004.
