# Supplementary material for: Paenidepsins are a Family of Lipopeptides from Paenibacillus
Source: J Nat Prod. 2026 Mar 3;89(3):966–79. doi: 10.1021/acs.jnatprod.5c01579 (PMC13036774; doi:10.1021/acs.jnatprod.5c01579)
Supplement: Supplementary file 2 [file np5c01579_si_002.zip › 1_paenidepsin_A/1_paenidepsin_A.rtf]

Spectrometer Manufacturer: Bruker BioSpin GmbHAcquisition Software: TopSpin 3.5, 3.6.5, and 3.7.0Processing Program: MestreNova 14.2.1Operating Frequencies:1H: 600 MHz; 700 MHz13C: 150 MHz (for 600 MHz instruments); 176 MHz (for 700 MHz instruments)1H_1H_COSY: 600 MHz (1H)1H_1H_ROESY: 600 MHz (1H)1H_1H_TOCSY: 600 MHz (1H)1H_13C_HMBC: 700 MHz (1H) / 176 MHz (13C)1H_13C_HSQC: 600 MHz / 150 MHz (13C)13C_DEPT-135: 150 MHz (13C)
