# Supplementary material for: Paenidepsins are a Family of Lipopeptides from Paenibacillus
Source: J Nat Prod. 2026 Mar 3;89(3):966–79. doi: 10.1021/acs.jnatprod.5c01579 (PMC13036774; doi:10.1021/acs.jnatprod.5c01579)
Supplement: Supplementary file 2 [file np5c01579_si_002.zip › 1_paenidepsin_A/13C/30_used_for_paper/30/pdata/1/email_MW14sLP23-7-24_30_1.pdf]

No parameters

ppm
